# Supplementary material for: Separation of benzene and toluene associated with vapochromic behaviors by hybrid[4]arene-based co-crystals
Source: Nat Commun. 2024 Feb 10;15:1260. doi: 10.1038/s41467-024-45592-6 (PMC10858960; doi:10.1038/s41467-024-45592-6)
Supplement: Supplementary file 1 — Supplementary Information [file 41467_2024_45592_MOESM1_ESM.pdf]

Supplementary Information

Separation of benzene and toluene associated with vapochromic behaviors by hybrid[4]arene-based co-crystals

Jingyu Chen,[1] Wenjie Zhang,[1] Wenzhi Yang,[1] Fengcheng Xi,[1] Hongyi He,[1] Minghao Liang,[1] Qian Dong,[1] Jiawang Hou,[1] Mengbin Wang,[2]* Guocan Yu,[3]* and Jiong Zhou[1]*

[1] Department of Chemistry, College of Sciences, Northeastern University, Shenyang 110819, P. R. China.

[2] ZJU-Hangzhou Global Scientific and Technological Innovation Center, Zhejiang University, Hangzhou 311215, P. R. China.

[3] Key Laboratory of Bioorganic Phosphorus Chemistry & Chemical Biology, Department of Chemistry, Tsinghua University, Beijing 100084, P. R. China.

* Corresponding author, E-mail: 21737055@zju.edu.cn; guocanyu@mail.tsinghua.edu.cn; zhoujiong@mail.neu.edu.cn

Table of Contents

1. Supplementary Methods 3
2. Supplementary Discussion 4
   - 2.1 Synthesis of hybrid[4]arene 4
   - 2.2 Crystallography data 10
   - 2.3 Characterization of the complexation of 1,2,4,5-tetracyanobenzene (TCNB) with hybrid[4]arene (H) in solution 13
   - 2.4 Non-covalent interaction analysis in single crystal structure of H-TCNB@CH_2Cl_2 17
   - 2.5 Characterization of H-TCNBα 21
   - 2.6 Single-component adsorption experiments 24
   - 2.7 Non-covalent interaction analysis in single crystal structure of H-TCNB@Bz 33
   - 2.8 Selectivity experiments of H-TCNBα for the mixture vapors of Bz and Cy 37
   - 2.9 Selectivity experiments of H-TCNBα for the mixture vapors of Tol and Py 39
   - 2.10 Purity experiments of liquid Cy or Py by H-TCNBα 43
   - 2.11 Recyclability of H-TCNBα 51
   - 2.12 Vapochromic behaviors experiments based on co-crystals of DON-TCNBα and DOB-TCNBα 64
3. Supplementary Reference 66

1. Supplementary Methods

Starting materials and reagents including TCNB, Bz, Cy, Tol and Py were purchased from commercial suppliers and used without further purification unless stated otherwise. Compound H was prepared according to a previous reported literature.[1] ^{1}H and ^{13}C NMR spectra were recorded at 298 K on a Bruker AVANCEIII 400-MHz instrument at room temperature. Chemical shifts were referenced to tetramethylsilane. Powder X-ray diffraction (PXRD) measurements were collected on a PANalytical B.V. Empyrean powder diffractometer operating at 40 kV/30 mA using the Cu Kα line (λ = 1.5418 Å), and data were measured over the range 5° to 40° in 5°/min steps over 7 min. Single-crystal X-ray diffraction data were collected by a Bruker D8 Venture diffractometer equipped with a PHOTON 100 CMOS detector, using Ga-Kα radiation (λ = 1.34139 Å) and Mo-Kα radiation (λ = 0.71073 Å). The structures were solved with SHELXT program using Direct Methods or Intrinsic Phasing; refined by full-matrix least-squares on $|F|^2$ by SHELXL; and interfaced through the program OLex2. Thermogravimetric analysis(TGA) and differential scanning calorimetry (DSC) experiment were carried out using a simultaneous thermal analyzer 449 F3 analyzer (NETZSCH Instruments) with an automated vertical overhead thermobalance. The samples were heated at 10 °C/min using N_2 as the protective gas. Fluorescent titration experiments were performed on a Shimadzu RF-5301PC spectrometer. UV-vis spectra in solution were collected on a Shimadzu UV-2550 spectrometer. Solid-state UV-visible spectra were measured by a reflectance mode on a PerkinElmer Lambda950 spectrometer from 200 to 800 nm with BaSO4 as a reference. Energy-minimized structure and electrostatic potential surface (EPS) were calculated by density functional theory (DFT) using the B3LYP hybrid function combined with 6-31G(d,p) basis set under Gaussian G09. Using single crystal superstructures as input files, independent gradient model (IGM)5 analyses were carried out by Multiwfn 3.6 program6 through function 20 (visual study of weak interaction) and visualized using VMD software. Gas Chromatography (GC) Analysis: GC measurements were carried out using an Agilent 7890B instrument configured with an FID detector and a DB-624 column (30 m × 0.53 mm × 3.0 μm). Samples were analyzed using headspace injections and were performed by incubating the sample at 100 °C for 10 min followed by sampling 1 mL of the headspace. The following GC method was used: the oven was programmed from 50 °C, and ramped in 10 °C min^{-1} increments to 150 °C with 15 min hold; the total run time was 25 min; the injection temperature was 250 °C; the detector temperature was 280 °C with nitrogen, air, and make-up flow rates of 35, 350, and 35 mL min^{-1}, respectively.

2. Supplementary Discussion

2.1. Synthesis of hybrid[4]arene

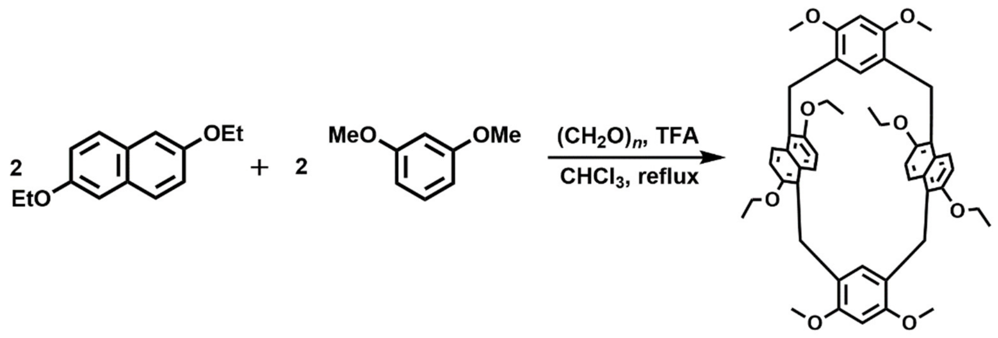

Synthesis of hybrid[4]arene (H): To the solution of 2,6-diethoxylnaphthalene (2.16 g, 10.0 mmol) and 1,3-dimethoxybenzene (1.38 g, 20.0 mmol) in $CHCl_3$ (200 mL), paraformaldehyde (0.600 g, 30.0 mmol) and TFA (10.0 mL) were added. The mixture was refluxed for 35 min. After the reaction was finished, a saturated aqueous solution of Na_2CO_3 was added to neutralize TFA. The organic phase was separated and the crude product was purified by column chromatography (petroleum ether/CH_2Cl_2, *v*/*v* 2:1) to get hybrid[4]arene (H) as a white solid (3.78 g, 50%), mp: 240-241 °C. The ^{1}H NMR spectrum of H is shown in Supplementary Figure 1. ^{1}H NMR (500 MHz, CD_2Cl_2, 293 K) δ (ppm): 7.24 (d, J = 10 Hz, 4H), 6.85 (d, J = 10 Hz, 4H), 6.63 (s, 2H), 5.71 (s, 2H), 4.51 (s, 2H), 4.47 (s, 2H), 3.99 (s, 12H), 3.95–3.93 (m, 4H), 3.91–3.88 (m, 4H), 3.70 (s, 2H), 3.66 (s, 2H), 1.24 (t, J = 15 Hz, 12H). The ^{13}C NMR spectrum of H is shown in Supplementary Figure 2. ^{13}C NMR (125 MHz, CD_2Cl_2, 293 K) δ (ppm): 156.32, 152.75, 130.02, 128.78, 124.21, 122.72, 120.73, 116.87, 95.17, 66.53, 66.53, 56.18, 54.26, 54.04, 53.83, 53.61, 53.39, 24.11, 15.50. Electrospray ionization mass spectrum of H is shown in Supplementary Figure 3: *m*/*z* 779.34 $[M + Na]^+$, 795.30 $[M + K]^+$. Electrospray ionization high resolution mass spectrum of H is shown in Supplementary Figure 4: *m*/*z* calcd for 779.3555 $[M + Na]^+$ $C_{48}H_{52}O_8Na^+$; found 779.3557; error –0.3 ppm.

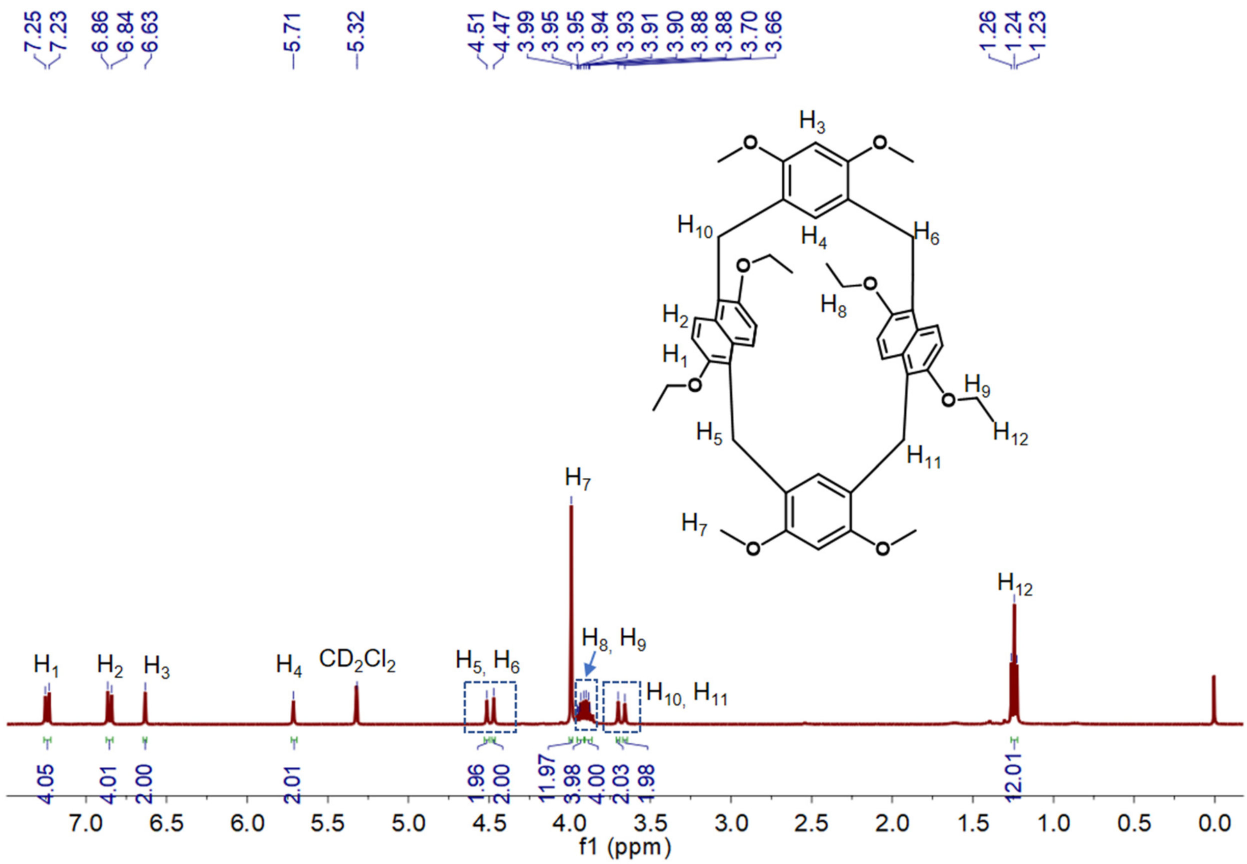

Supplementary Figure 1. ^{1}H NMR spectrum (500 MHz, CD_2Cl_2, 293 K) of H.

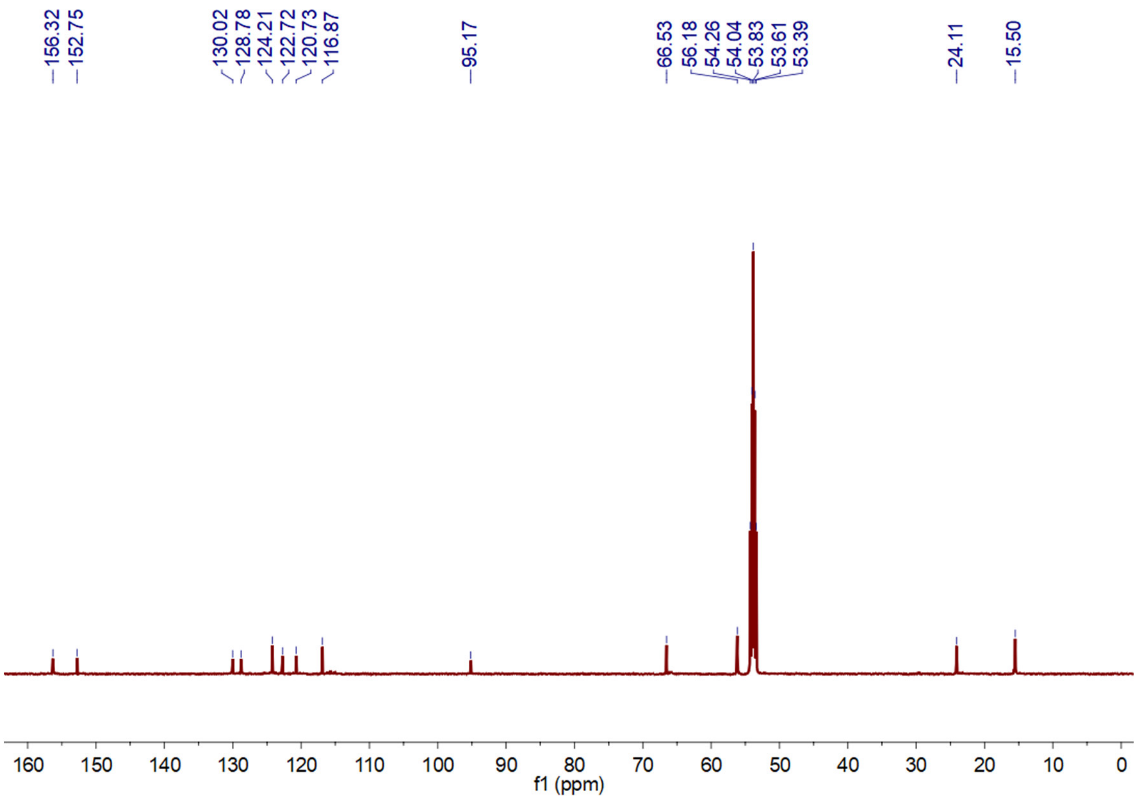

Supplementary Figure 2. ^{13}C NMR spectrum (125 MHz, CD_2Cl_2, 293 K) of H.

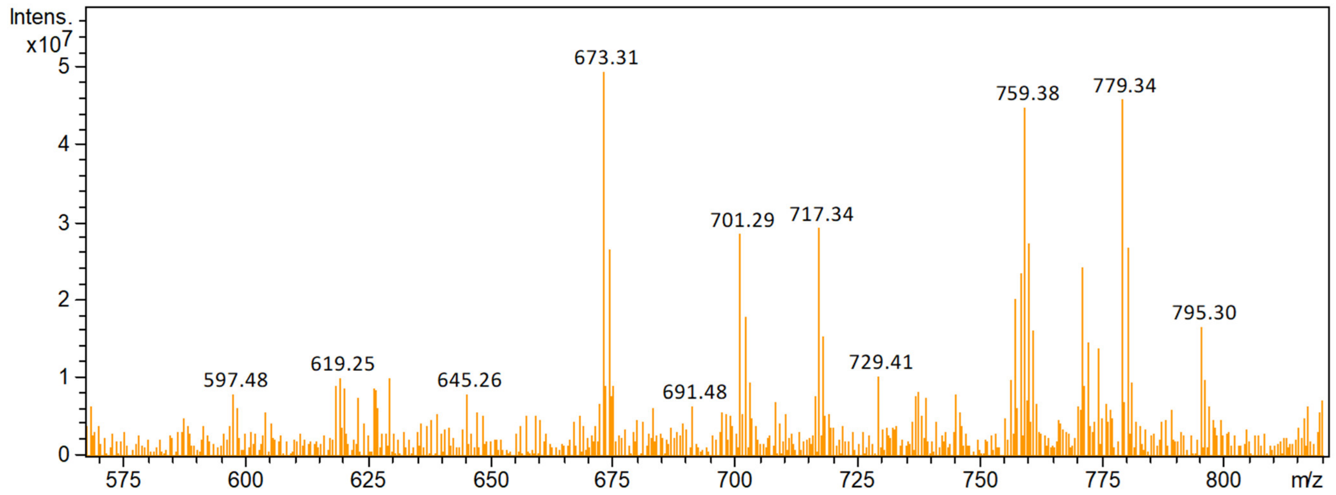

Supplementary Figure 3. Electrospray ionization mass spectrum of H. Assignment of main peaks: *m/z* 779.34 $[M + Na]^+$, 795.30 $[M + K]^+$.

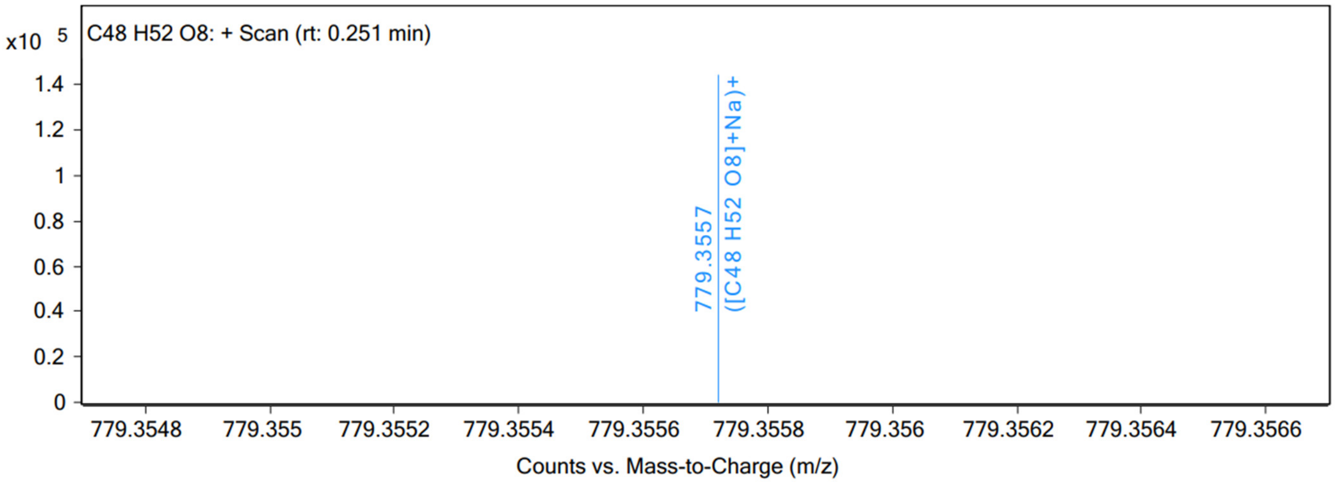

Supplementary Figure 4. Electrospray ionization high resolution mass spectrum of H. Assignment of main peak: *m/z* 779.3557 $[M + Na]^+$.

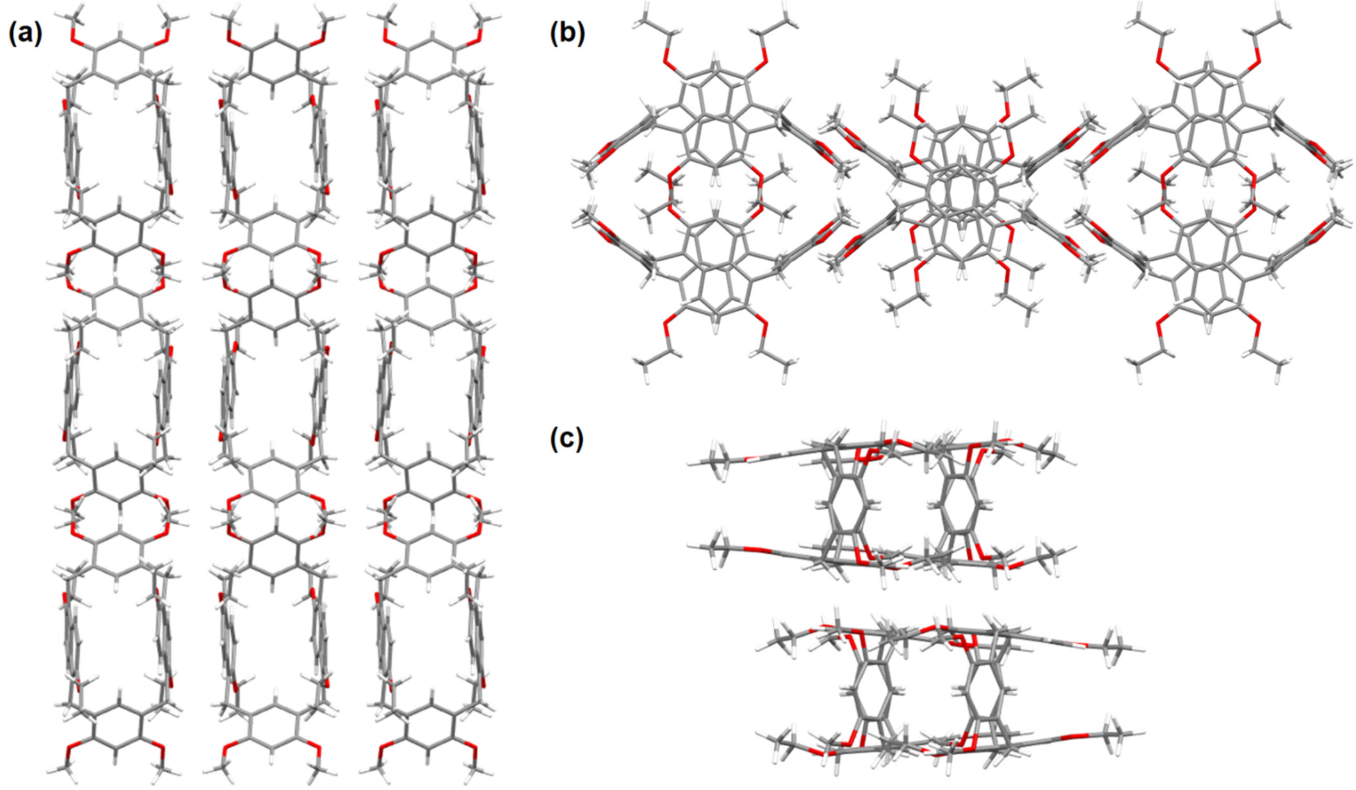

Supplementary Figure 5. Three views of the crystal packing structure of H. (a) *a*-axi, (b) *b*-axi and (c) *c*-axi.

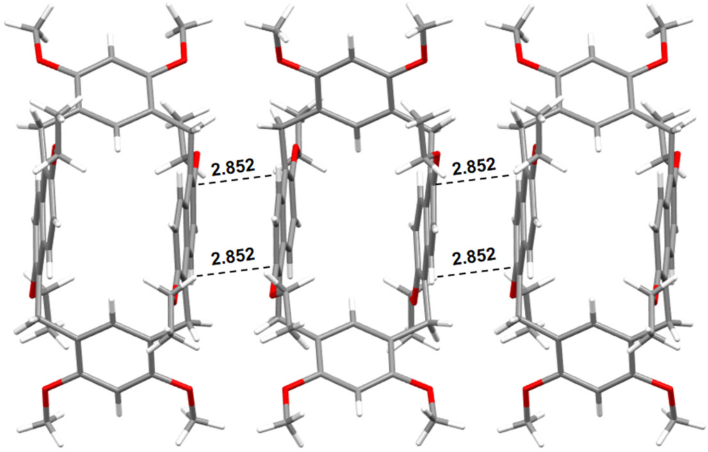

Supplementary Figure 6. H was arranged parallel to its neighbor H through intermolecular C–H⋯π interactions. C–H⋯π distance: 2.852 Å.

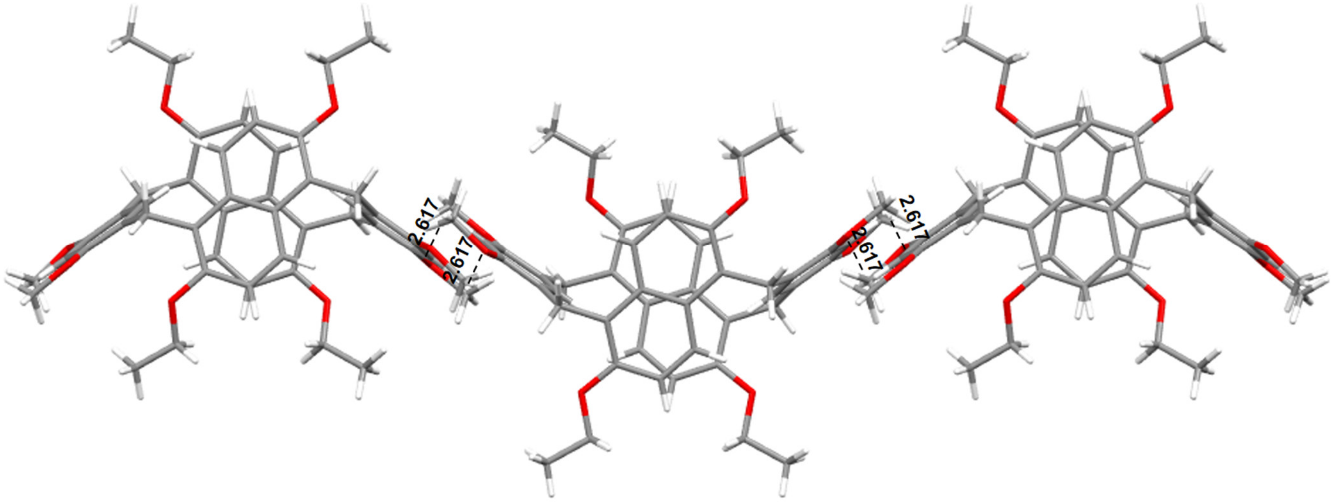

Supplementary Figure 7. The crystal packing structure of H along *b*-axis. H was arranged parallel to its neighbor H through intermolecular C–H···O interactions. C–H···O distance: 2.671 Å.

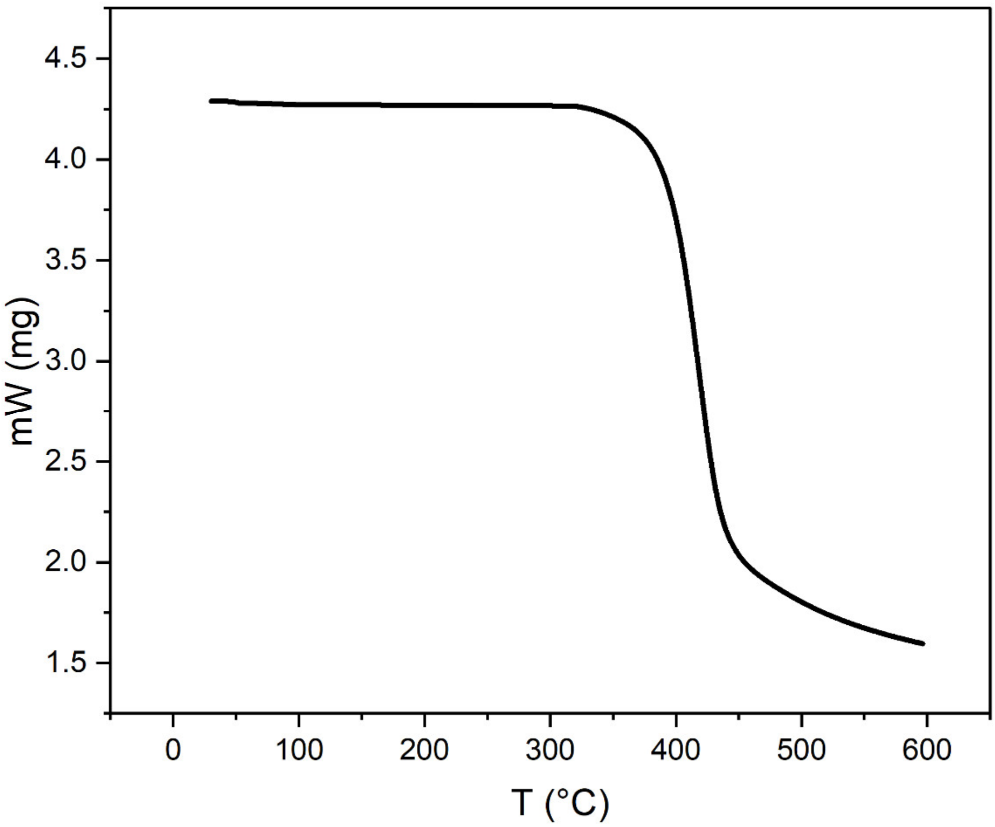

Supplementary Figure 8. Thermogravimetric analysis of Hα.

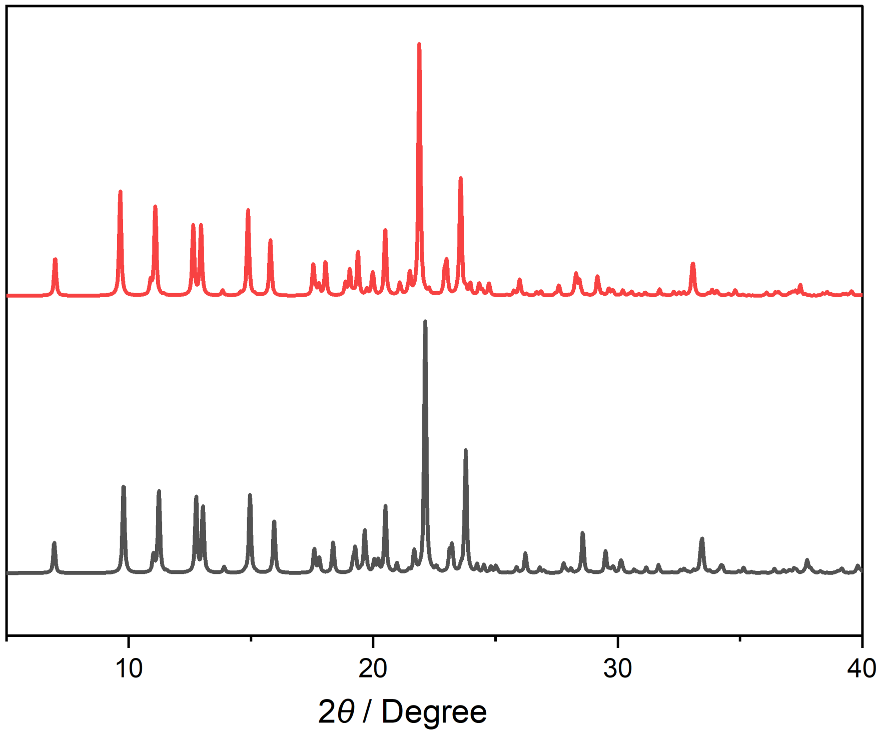

Supplementary Figure 9. Powder X-ray diffraction patterns of H: black line, original Hα; red line, simulated from single crystal structure of H.

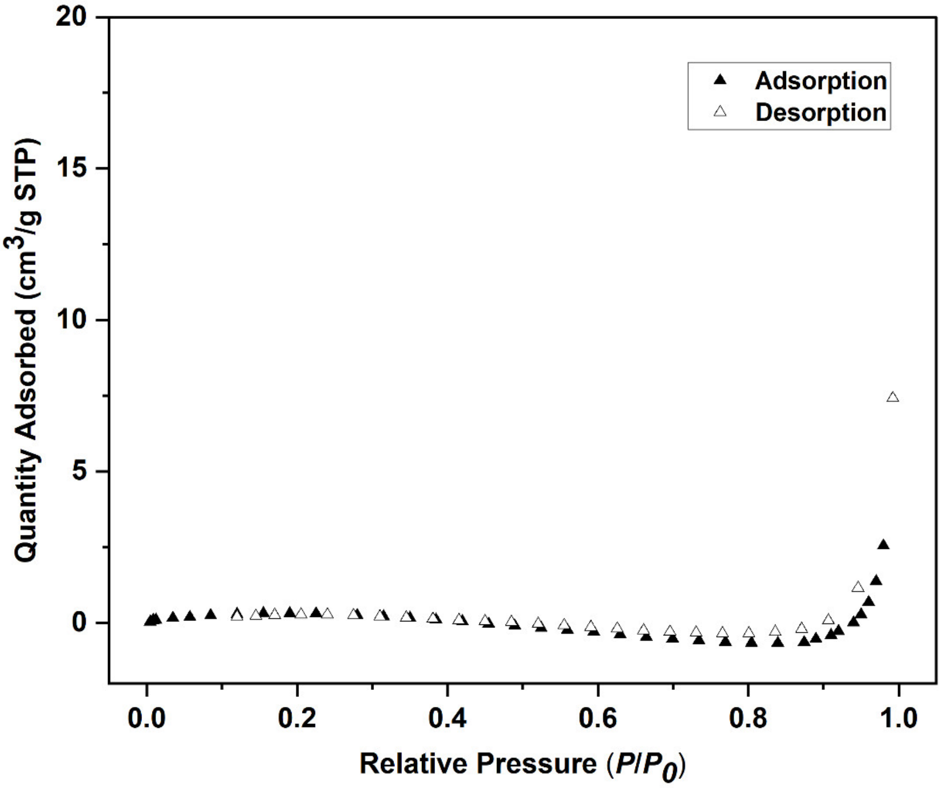

Supplementary Figure 10. N_2 adsorption-desorption isotherm of Hα. The BET surface area value is 0.820 m^2/g. Adsorption, closed symbols; desorption, open symbols.

2.2 Crystallography data

Supplementary Table 1. Experimental single crystal X-ray data for H.

Formula	H
Crystallization Solvent	CH_2Cl_2
Collection Temperature (K)	170(2)
Formula	$C_{48}H_{52}O_8$
Formula Weight	756.89
Crystal System	Orthorhombic
Space Group	*Pbcn*
a [Å]	25.3974(9)
b [Å]	9.6581(5)
c [Å]	16.0561(7)
α [o]	90
β [o]	90
γ [o]	90
V [Å^3]	3938.4(3)
Z	4
D_{calcd} [g cm^{-3}]	1.277
Absorption coefficient (mm^{-1})	0.086
F(000)	1616.0
Theta range [º]	4.512 to 54.24
Reflections collected / unique	57861 [R_{int} = 0.0441]
Data / restraints / parameters	4353 / 18 / 267
Final *R* indices [I>2σ(I)]	R_1 = 0.0399, wR_2 = 0.1017
R indices (all data)	R_1 = 0.0515, wR_2 = 0.1104
Goodness-of-fit on F^2	1.034
Largest difference peak and hole [e.A^{-3}]	0.21 and -0.17
CCDC	2244801

Supplementary Table 2. Experimental single crystal X-ray data for H-TCNB@CH_2Cl_2.

Formula	H-TCNB@CH_2Cl_2
Crystallization Solvent	CH_2Cl_2
Collection Temperature (K)	273(2)
Formula	$C_{60}H_{58}Cl_4N_4O_8$
Formula Weight	1104.90
Crystal System	Orthorhombic
Space Group	*Fdd2*
a [Å]	26.7226(9)
b [Å]	35.7156(12)
c [Å]	11.5081(4)
α [o]	90
β [o]	90
γ [o]	90
V [Å^3]	10983.5(6)
Z	8
D_{calcd} [g cm^{-3}]	1.336
Absorption coefficient (mm^{-1})	0.275
F(000)	4624.0
Theta range [º]	2.95 to 26.40
Reflections collected / unique	13330 / 4718 [R_{int} = 0.0203]
Data / restraints / parameters	4718 / 1 / 347
Final R indices [I>2σ(I)]	R_1 = 0.0817, wR_2 = 0.0817
R indices (all data)	R_1 = 0.0842, wR_2 = 0.0842
Goodness-of-fit on F^2	1.054
Largest difference peak and hole [e.A^{-3}]	0.254 and -0.342
CCDC	2244721

Supplementary Table 3. Experimental single crystal X-ray data for H-TCNB@Bz.

Formula	H-TCNB@Bz
Crystallization Solvent	benzene
Collection Temperature (K)	170(2)
Formula	$C_{70}H_{66}N_4O_8$
Formula Weight	1091.27
Crystal System	Orthorhombic
Space Group	*Fdd2*
a [Å]	26.4248(8)
b [Å]	36.2735(16)
c [Å]	11.9131(3)
α [o]	90
β [o]	90
γ [o]	90
V [Å^3]	11418.9(7)
Z	8
D_{calcd} [g cm^{-3}]	1.270
Absorption coefficient (mm^{-1})	0.083
F(000)	4624.0
Theta range [º]	4.492 to 54.22
Reflections collected / unique	32095 / 6116 [R_{int} = 0.0415]
Data / restraints / parameters	6116 / 1 / 374
Final *R* indices [I>2$\sigma(I)$]	R_1 = 0.0323, wR_2 = 0.0323
R indices (all data)	R_1 = 0.0372, wR_2 = 0.0372
Goodness-of-fit on F^2	1.045
Largest difference peak and hole [e.A^{-3}]	0.127 and -0.196
CCDC	2244722

2.3. Characterization of the complexation of 1,2,4,5-tetracyanobenzene (TCNB) with hybrid[4]arene (H) in solution

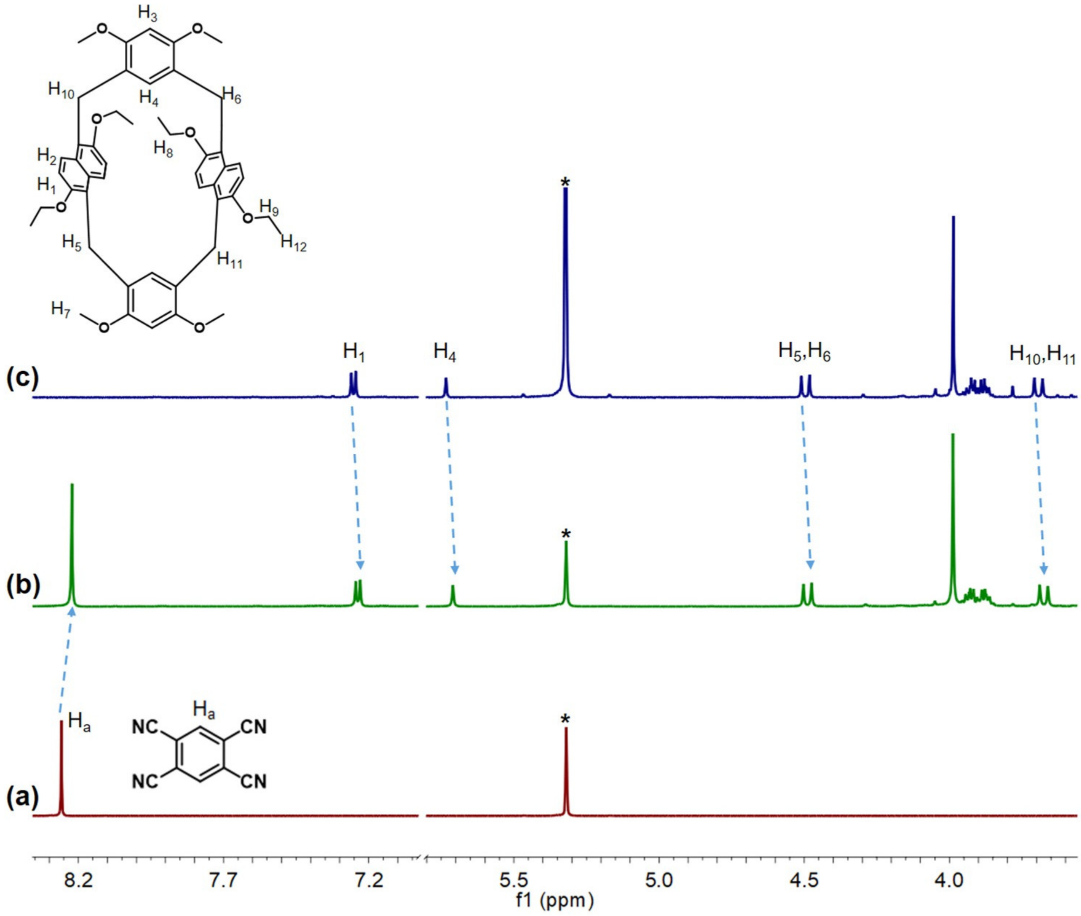

Supplementary Figure 11. Partial ^{1}H NMR spectra (500 MHz, CD_2Cl_2, 293 K) of (a) free TCNB (10.0 mM), (b) H (10.0 mM) and TCNB (10.0 mM), and (c) free H (10.0 mM). (*: solvent residues)

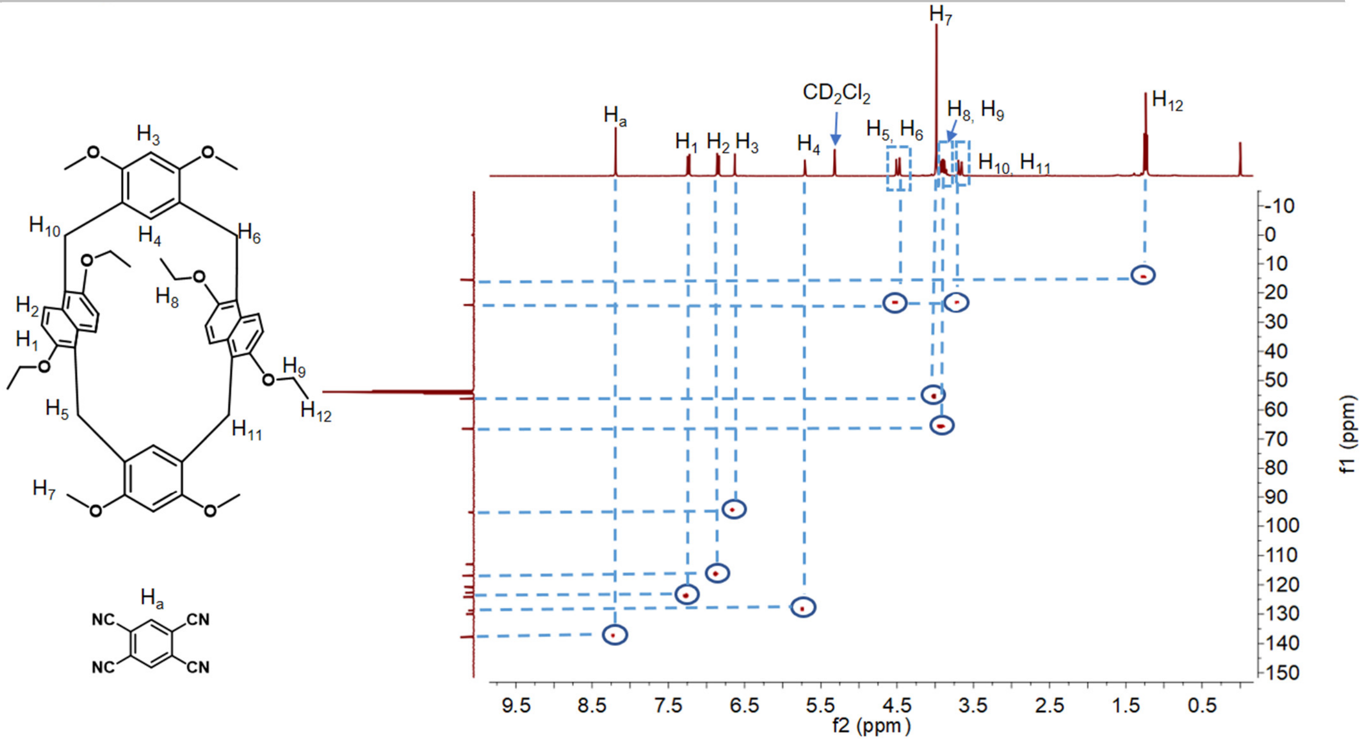

Supplementary Figure 12. HSQC spectrum (500 MHz, CD_2Cl_2, 298 K) of H (10.0 mM) with TCNB (10.0 mM).

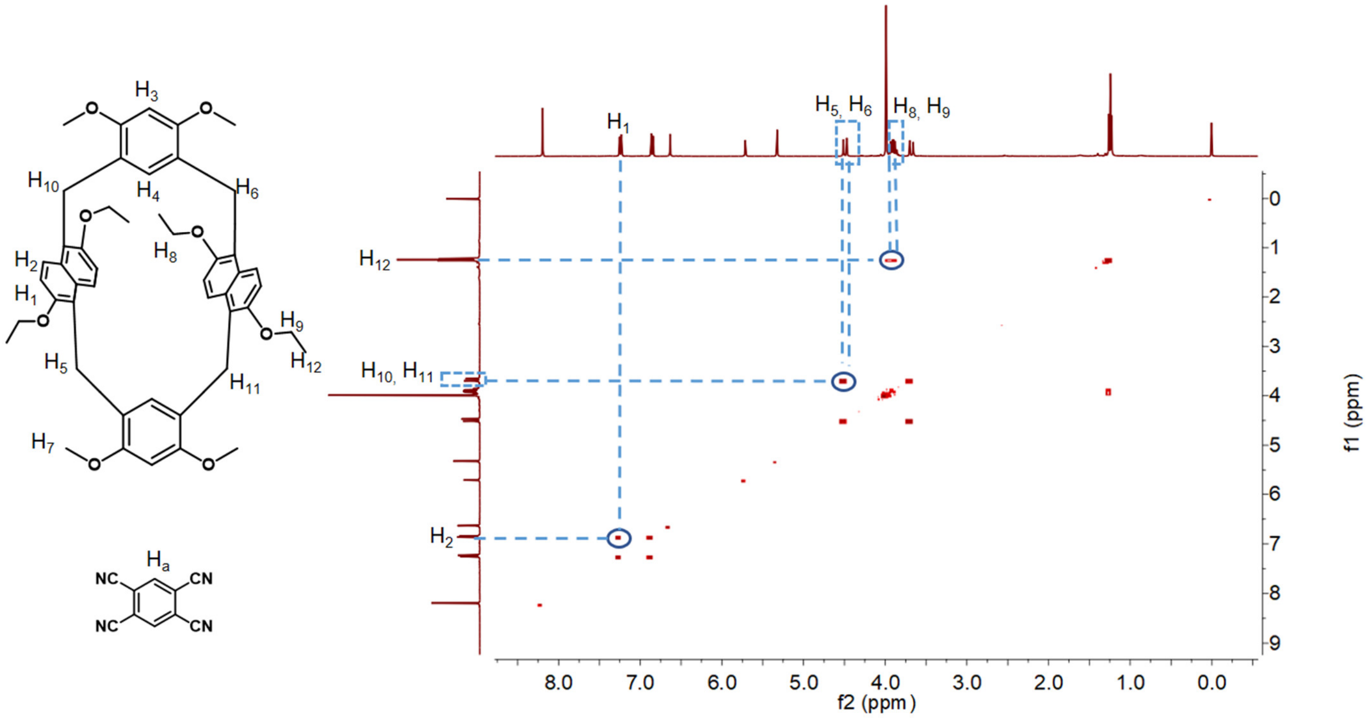

Supplementary Figure 13. 2D COSY spectrum (500 MHz, CD_2Cl_2, 298 K) of H (10.0 mM) with TCNB (10.0 mM).

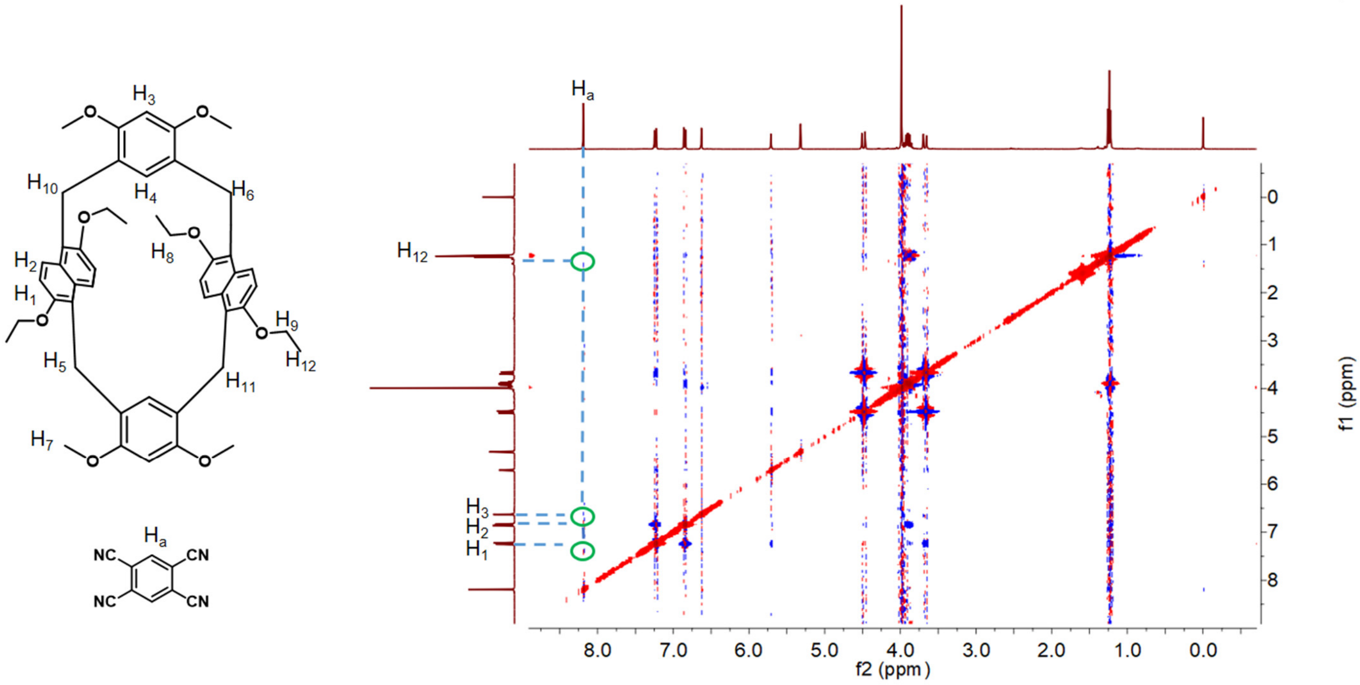

Supplementary Figure 14. 2D NOSEY spectrum (500 MHz, CD_2Cl_2, 298 K) of H (10.0 mM) with TCNB (10.0 mM).

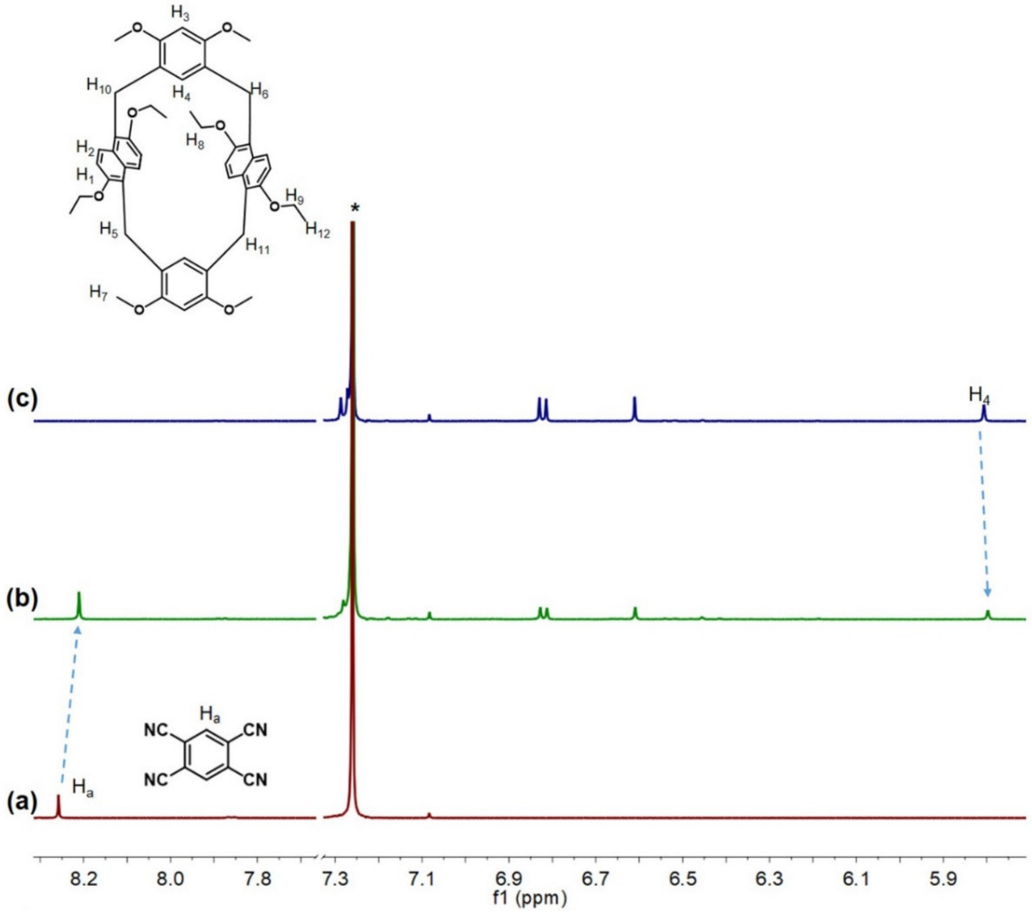

Supplementary Figure 15. Partial ^1H NMR spectra (500 MHz, $CDCl_3$, 293 K) of (a) free TCNB (10.0 mM), (b) H (10.0 mM) and TCNB (10.0 mM), and (c) free H (10.0 mM). (*: solvent residues)

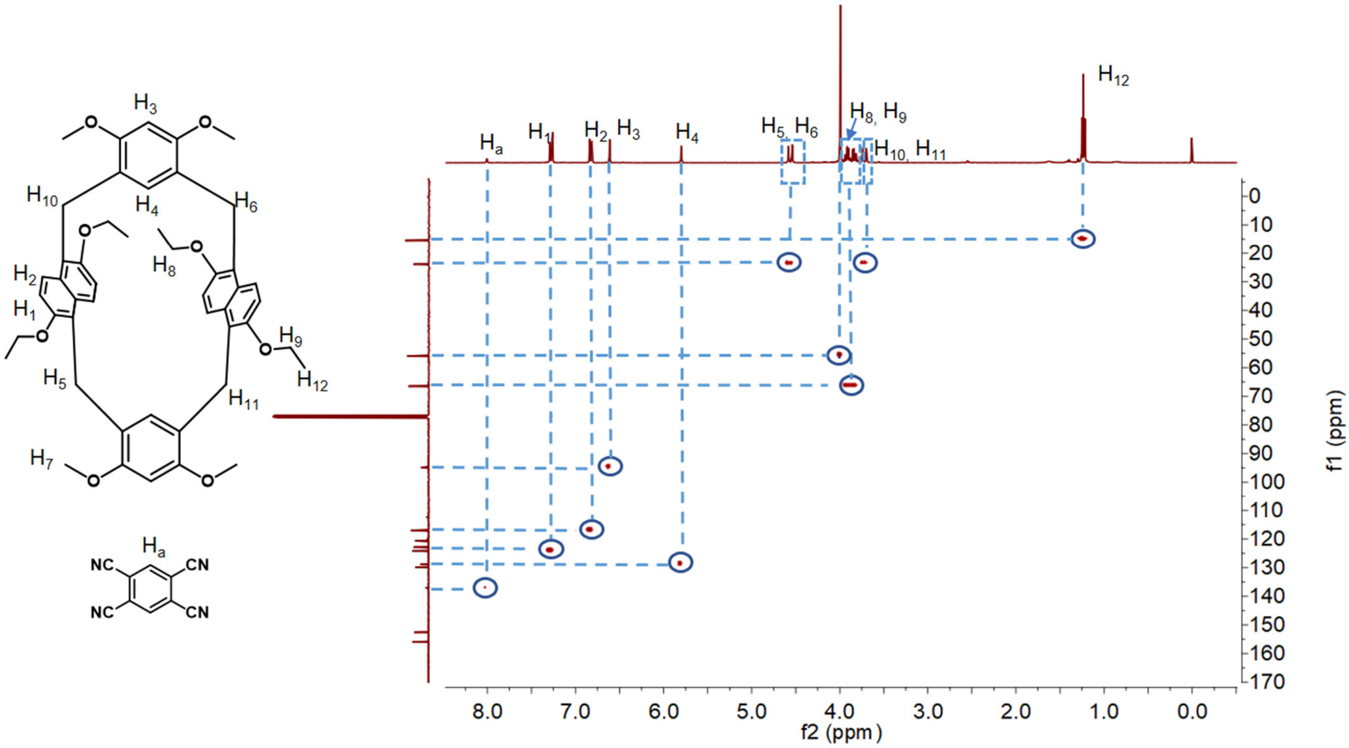

Supplementary Figure 16. HSQC spectrum (500 MHz, $CDCl_3$, 298 K) of H (10.0 mM) with TCNB (10.0 mM).

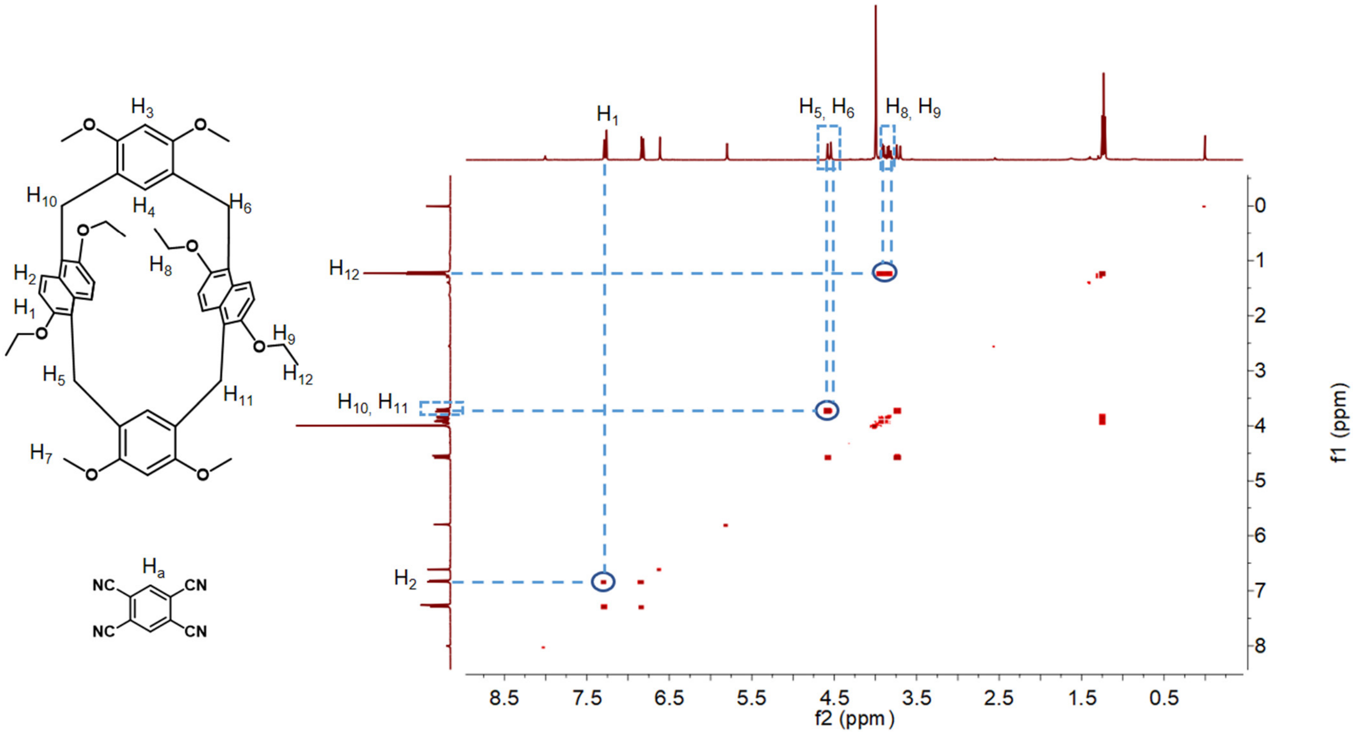

Supplementary Figure 17. 2D COSY spectrum (500 MHz, $CDCl_3$, 298 K) of H (10.0 mM) with TCNB (10.0 mM).

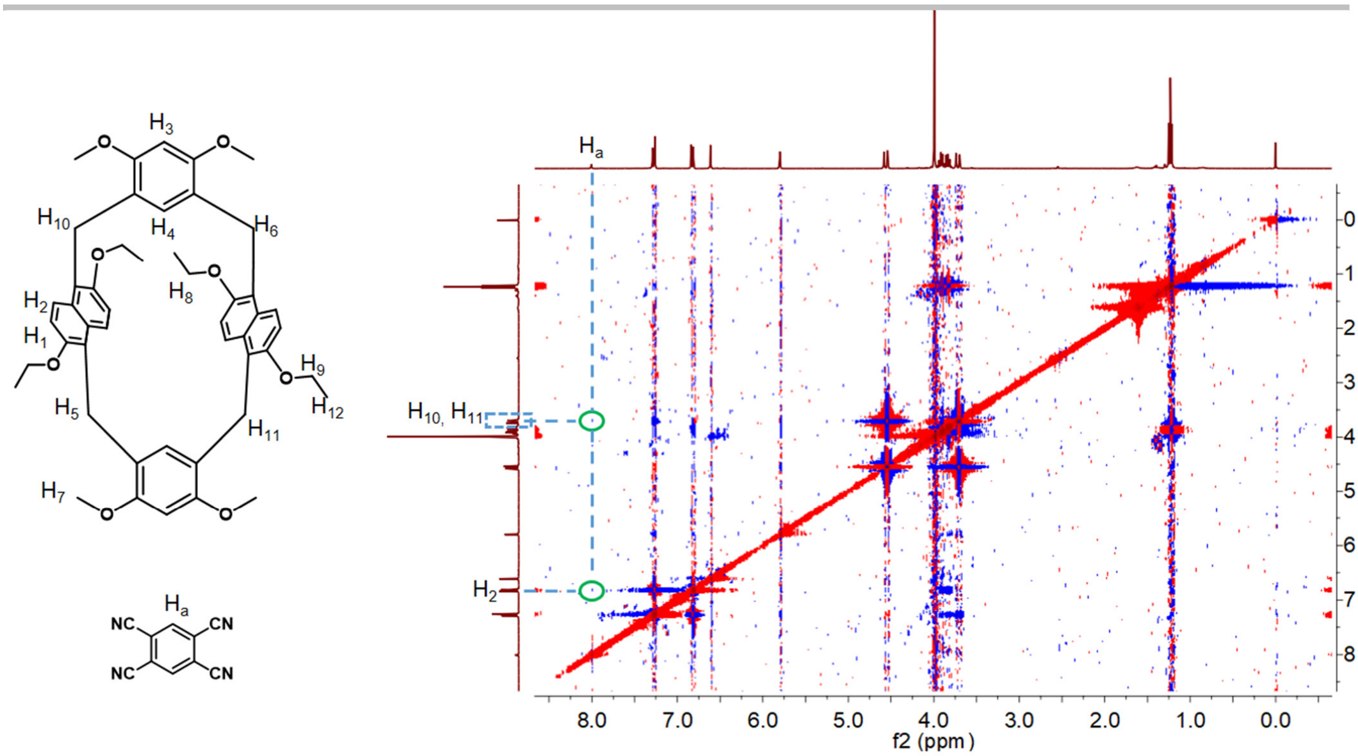

Supplementary Figure 18. 2D NOSEY spectrum (500 MHz, $CDCl_3$, 298 K) of H (10.0 mM) with TCNB (10.0 mM).

2.4. Non-covalent interaction analysis in single crystal structure of H-TCNB@CH_2Cl_2

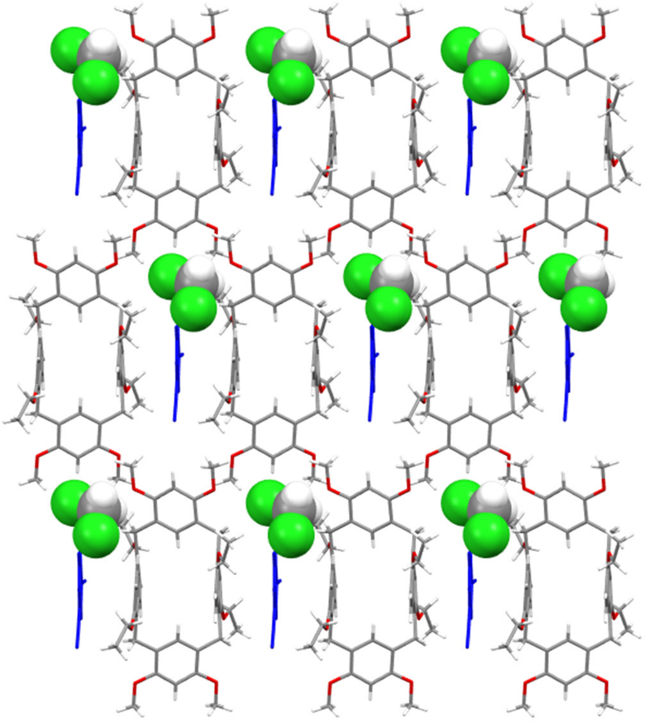

Supplementary Figure 19. Illustration of the packing model of H-TCNB@CH_2Cl_2.

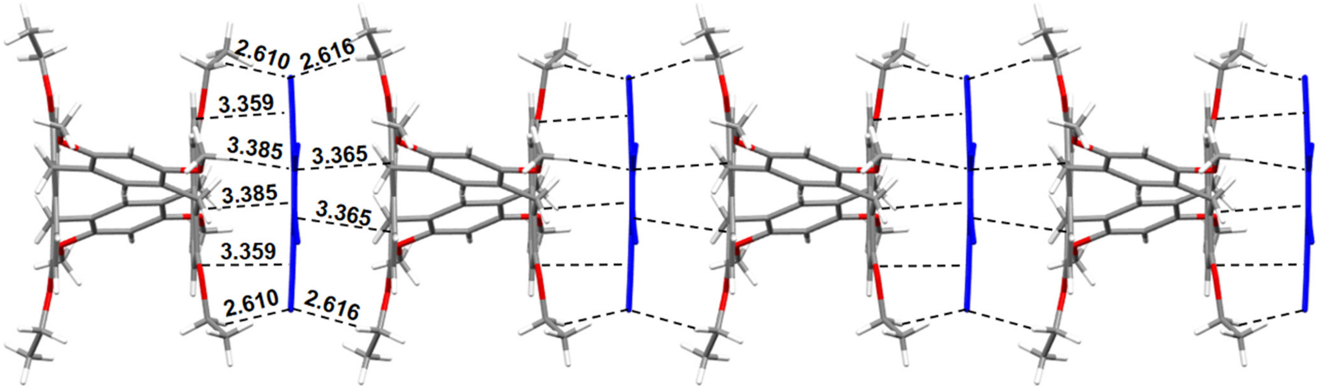

Supplementary Figure 20. The exo-wall π–π interaction between H and TCNB in the co-crystal structures of H-TCNB@CH_2Cl_2. The CH_2Cl_2 molecules are omitted for clarity.

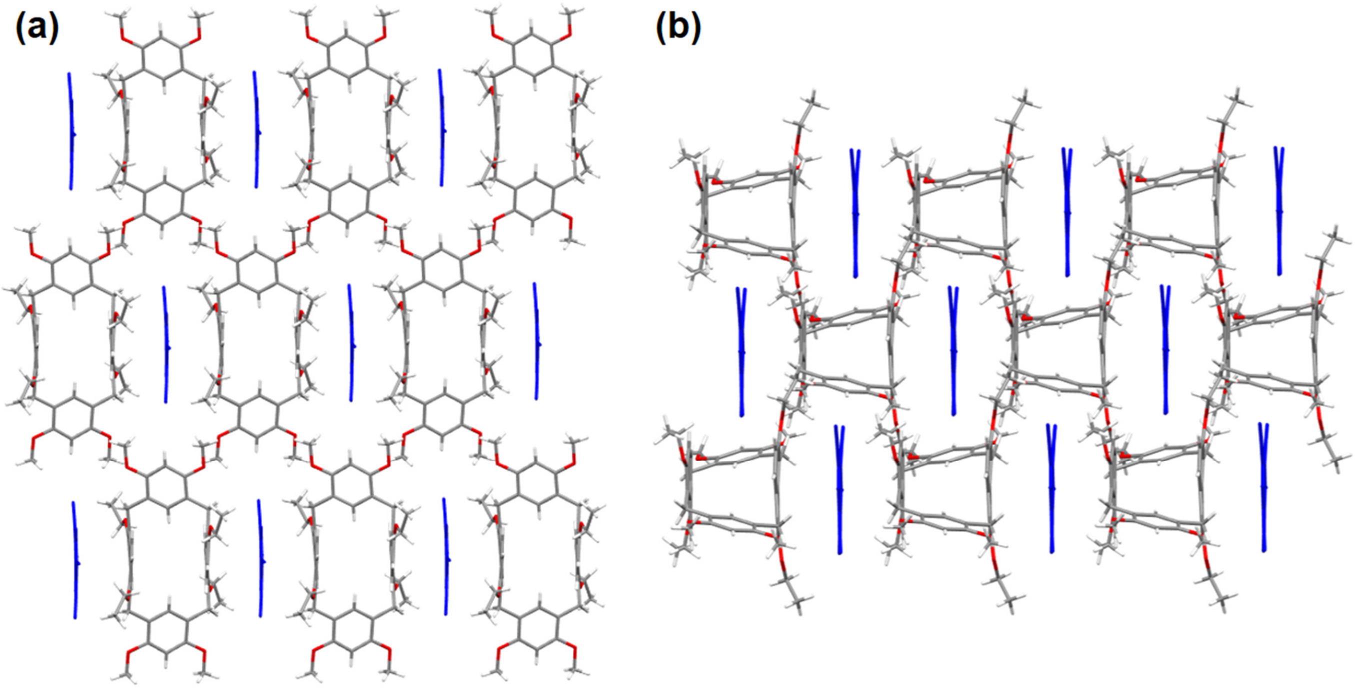

Supplementary Figure 21. Illustration of the packing model of H-TCNB@CH_2Cl_2. (a) Side view, (b) Top view. The CH_2Cl_2 molecules are omitted for clarity.

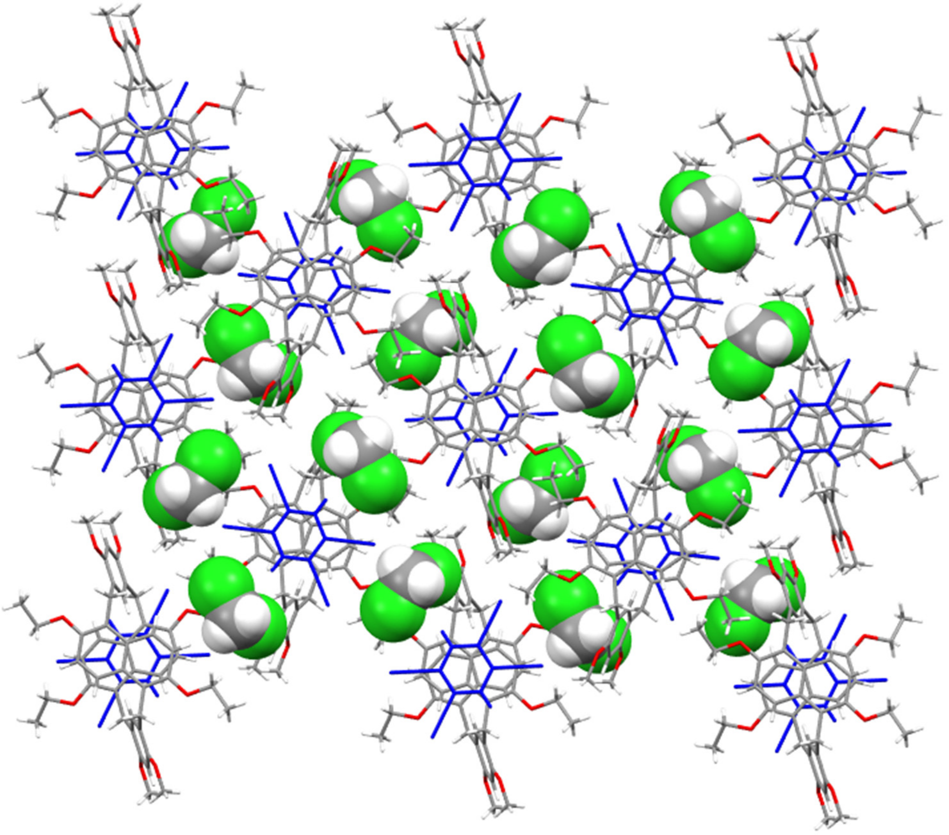

Supplementary Figure 22. Illustration of the packing model of H-TCNB@CH_2Cl_2 along *a*-axis.

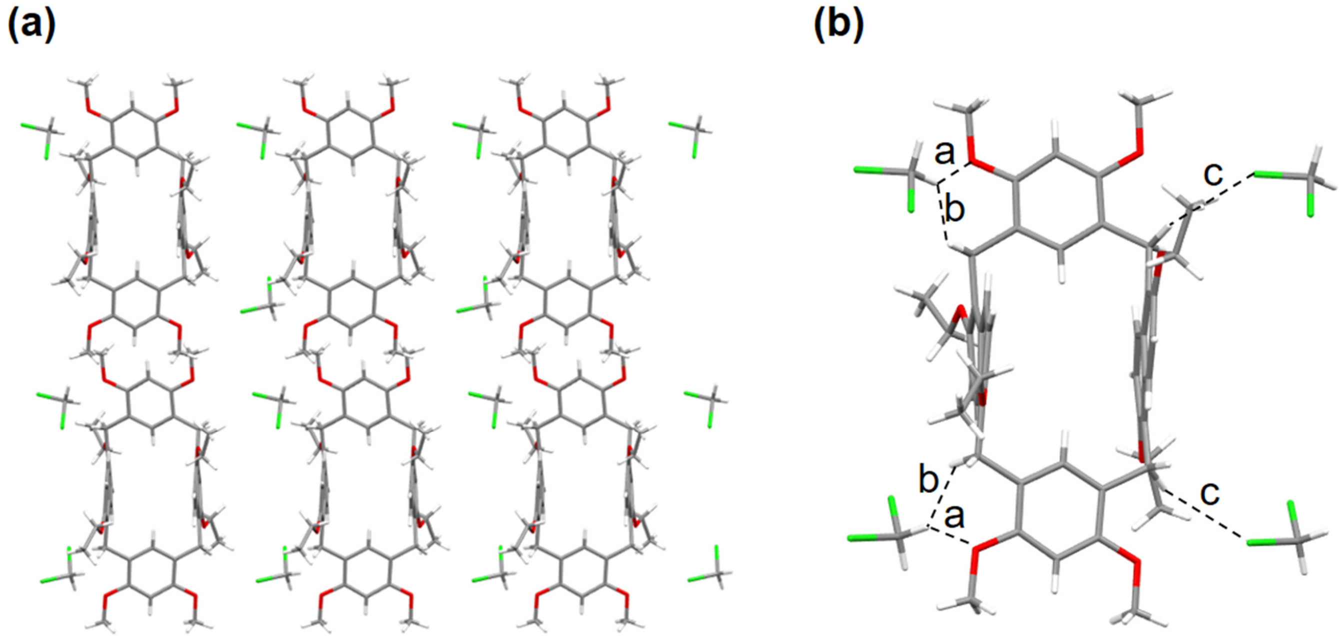

Supplementary Figure 23. Illustration of the (a) packing model of H-TCNB@CH_2Cl_2 and (b) C–H···O and C–H···Cl interactions between CH_2Cl_2 and H. The TCNB molecules are omitted for clarity. C–H···O distances: a = 2.419 Å, b = 2.343 Å. C–H···Cl distance: c = 2.936 Å.

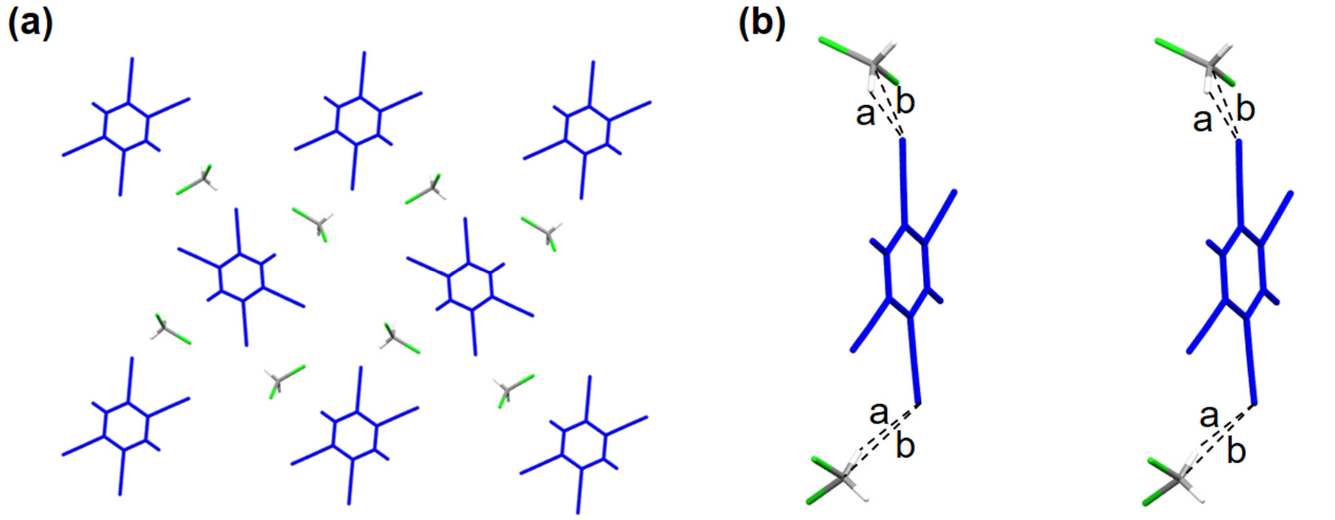

Supplementary Figure 24. Illustration of the (a) packing model of H-TCNB@CH_2Cl_2 and (b) C–H···N interactions between CH_2Cl_2 and TCNB. The H molecules are omitted for clarity. C–H···N distances: a = 2.342 Å, b = 3.218 Å.

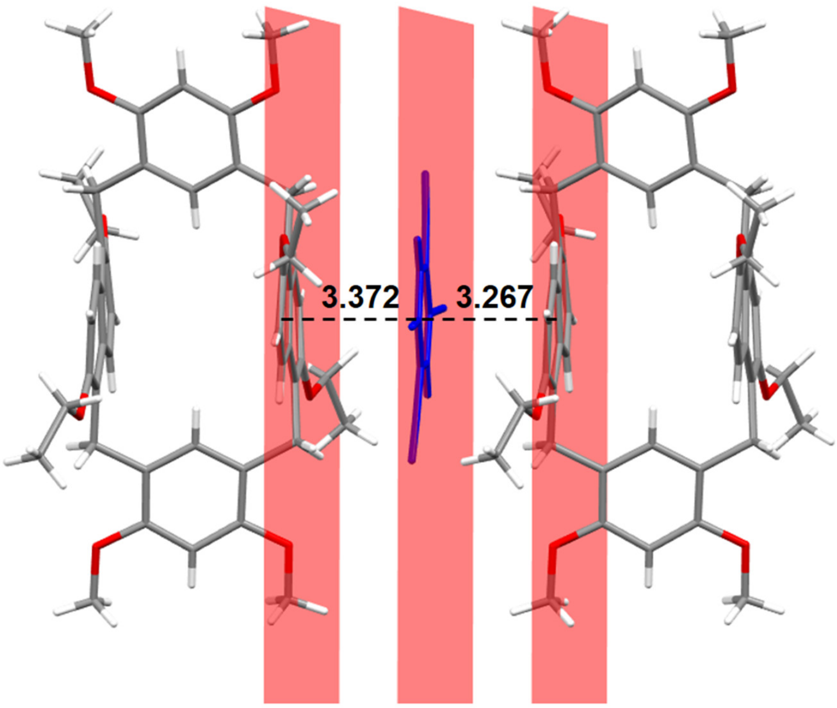

Supplementary Figure 25. The exo-wall π–π interaction between H and TCNB in the co-crystal structures of H-TCNB@CH_2Cl_2. The CH_2Cl_2 molecules are omitted for clarity. π–π distances: 3.372 Å and 3.267 Å.

2.5. Characterization of H-TCNBα

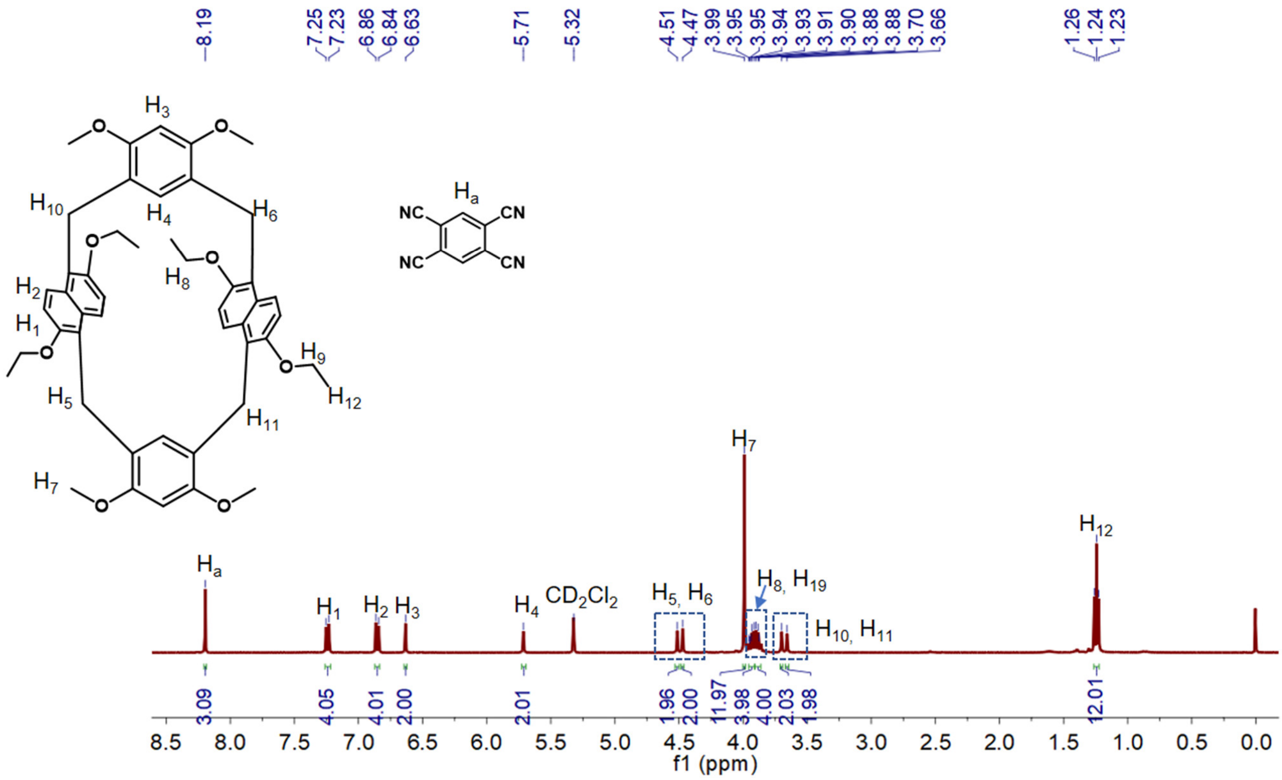

Supplementary Figure 26. ^{1}H NMR spectrum (500 MHz, CD_2Cl_2, 293 K) of H-TCNBα.

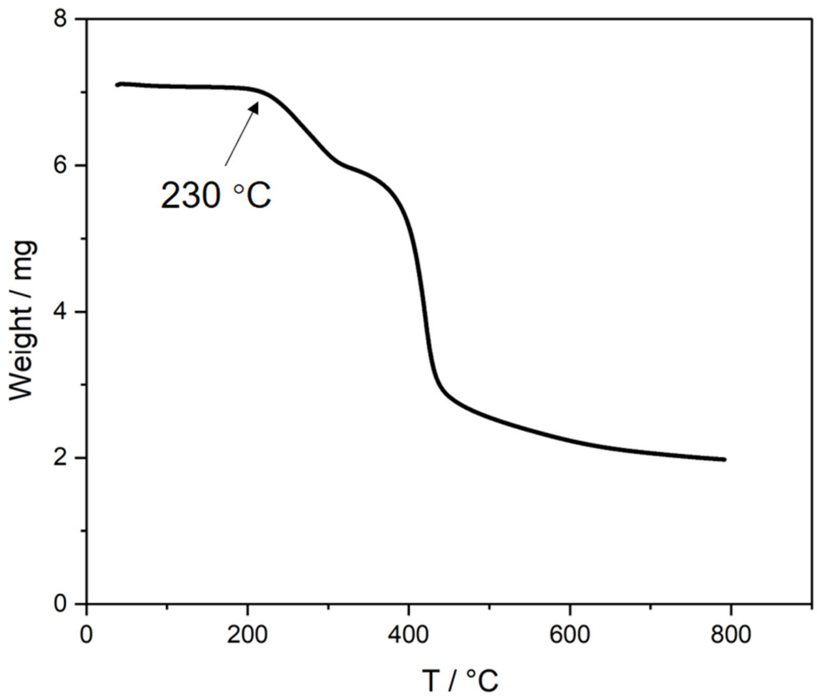

Supplementary Figure 27. Thermogravimetric analysis of H-TCNBα.

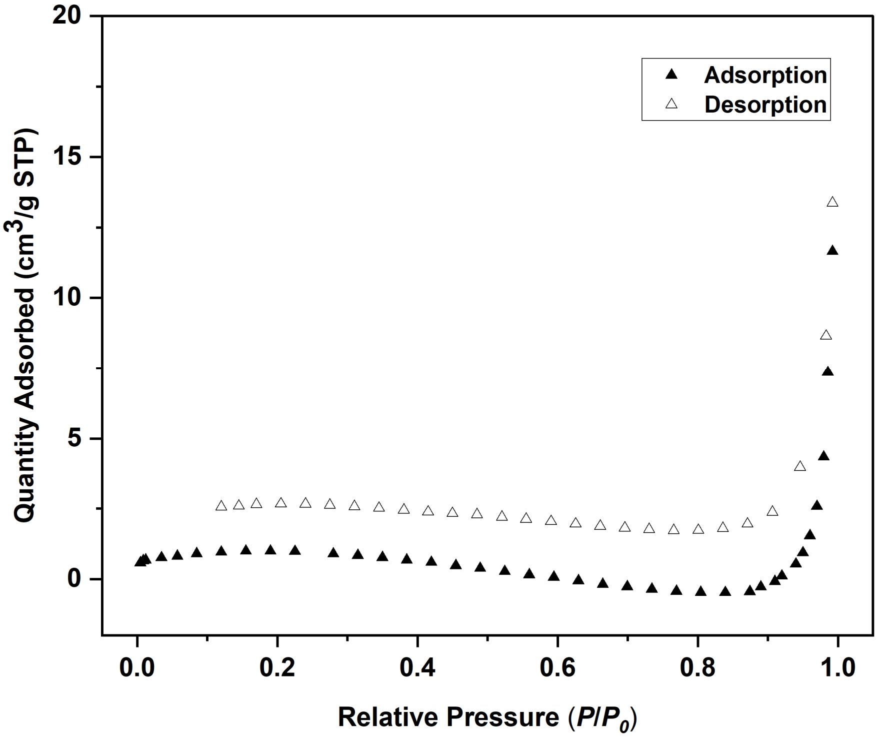

Supplementary Figure 28. N_2 adsorption-desorption isotherm of H-TCNBα. The BET surface area value is 0.970 m^2/g. Adsorption, closed symbols; desorption, open symbols.

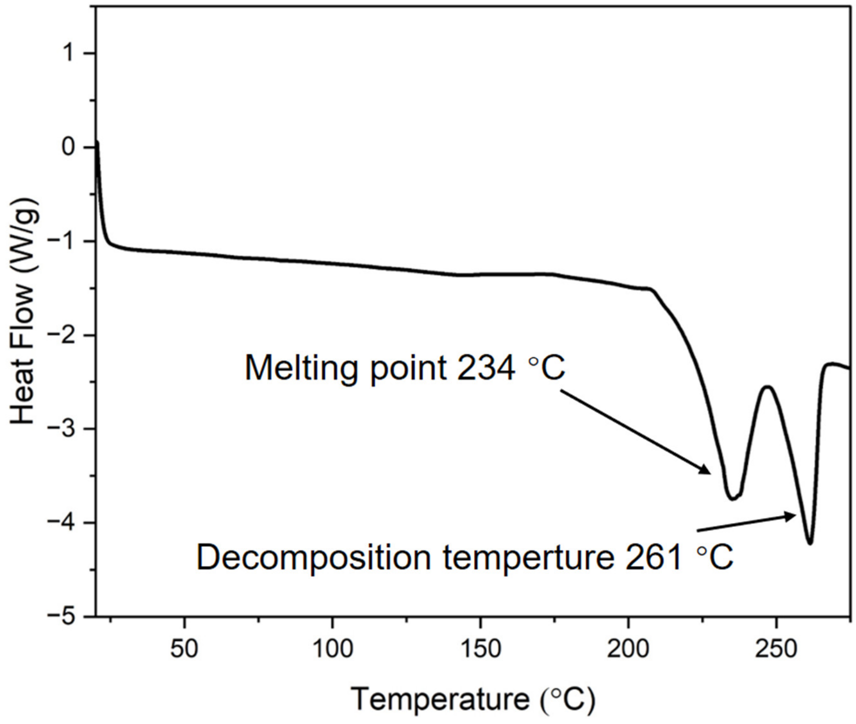

Supplementary Figure 29. DSC plot of H-TCNBα.

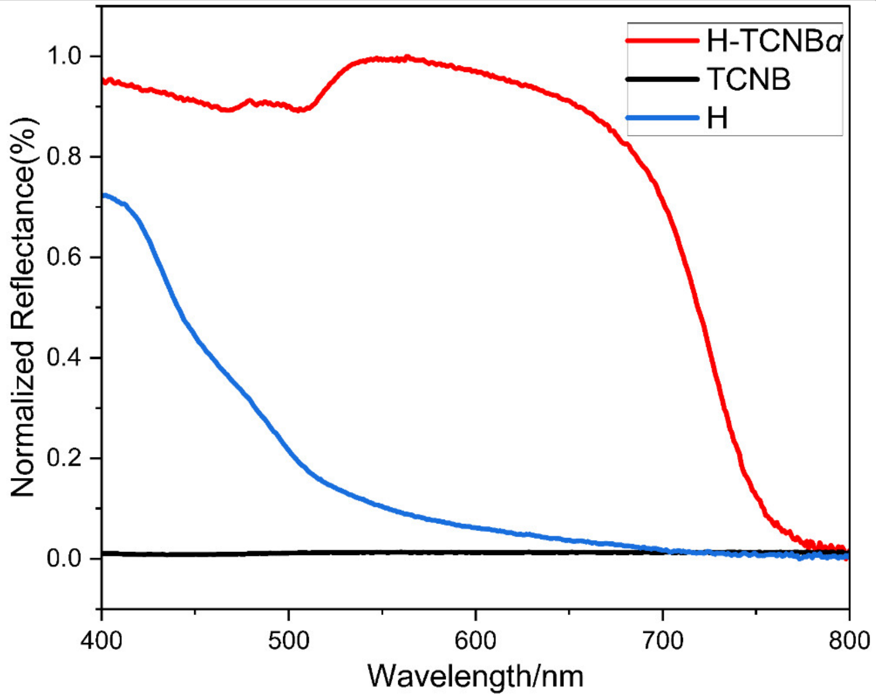

Supplementary Figure 30. Diffuse reflectance spectra of H, TCNB and H-TCNBα.

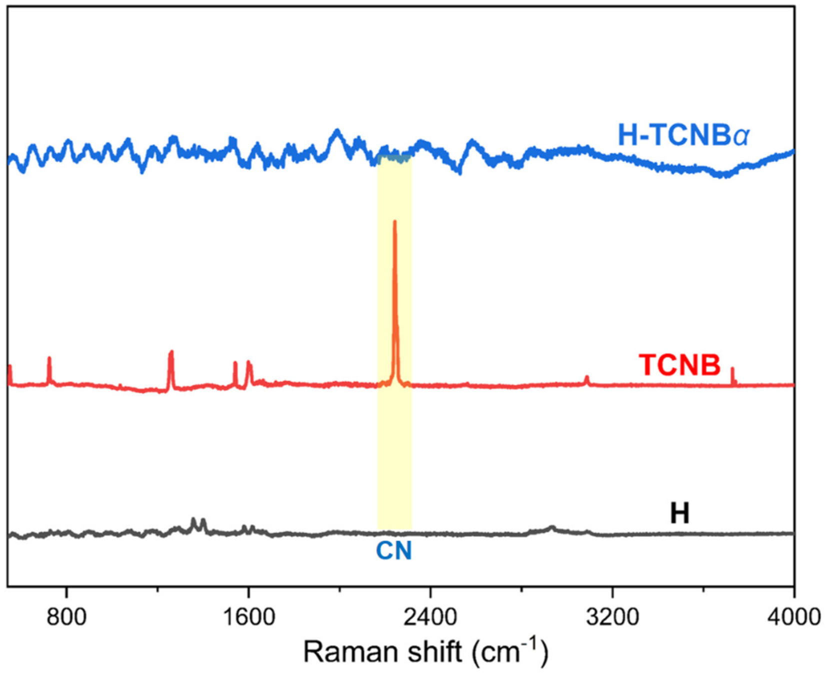

Supplementary Figure 31. Raman spectra of H, TCNB, H-TCNBα.

2.6. Single-component adsorption experiments

^{1}H NMR experiments were performed by dissolving H-TCNBα after adsorption of single-component Bz/Cy vapor in CD_2Cl_2. TGA profiles were recorded using H-TCNBα after adsorption of single-component Bz/Cy vapor.[1]

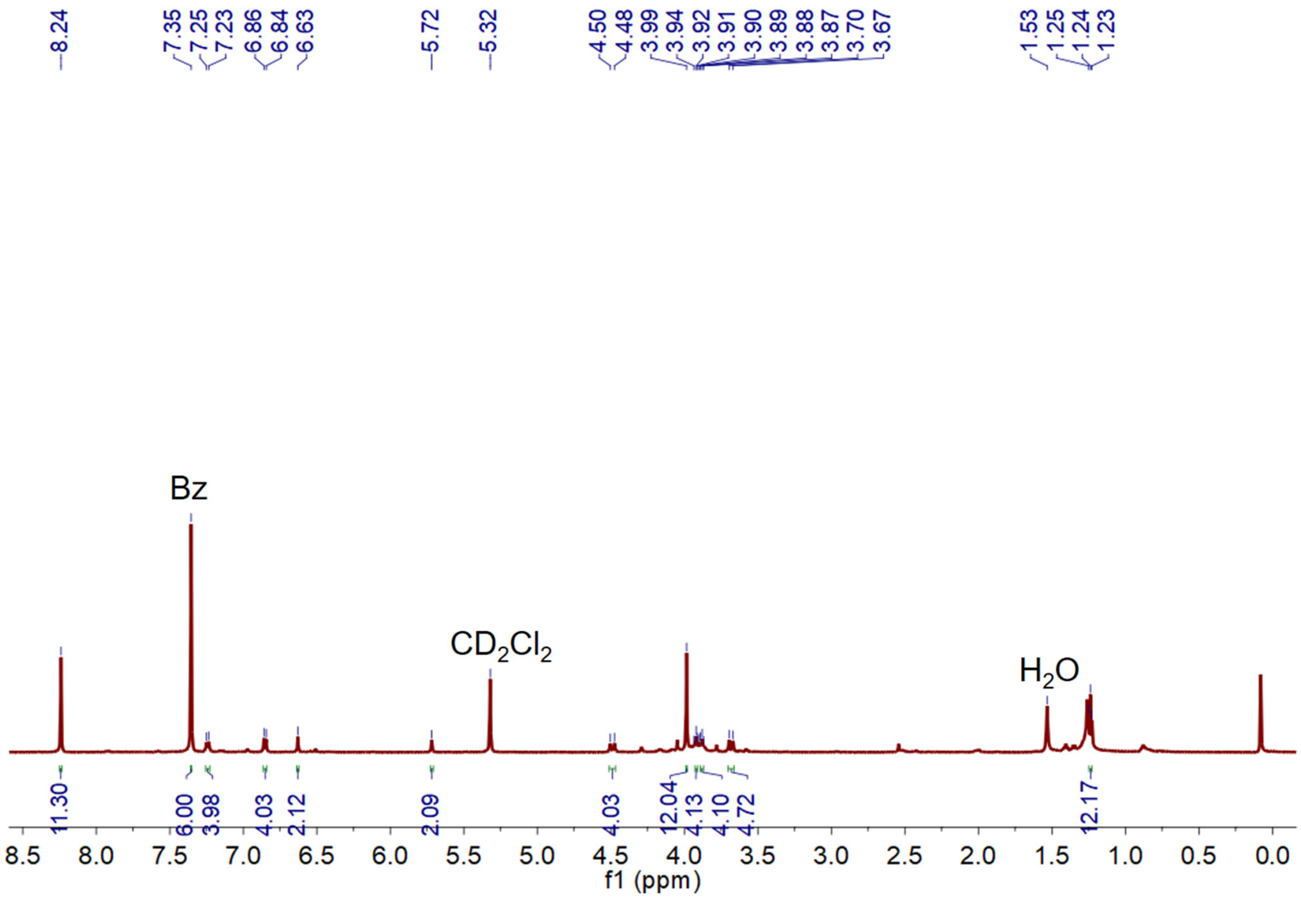

Supplementary Figure 32. ^{1}H NMR spectrum (500 MHz, CD_2Cl_2, 293 K) of H-TCNBα after adsorption of Bz vapor for 24 h.

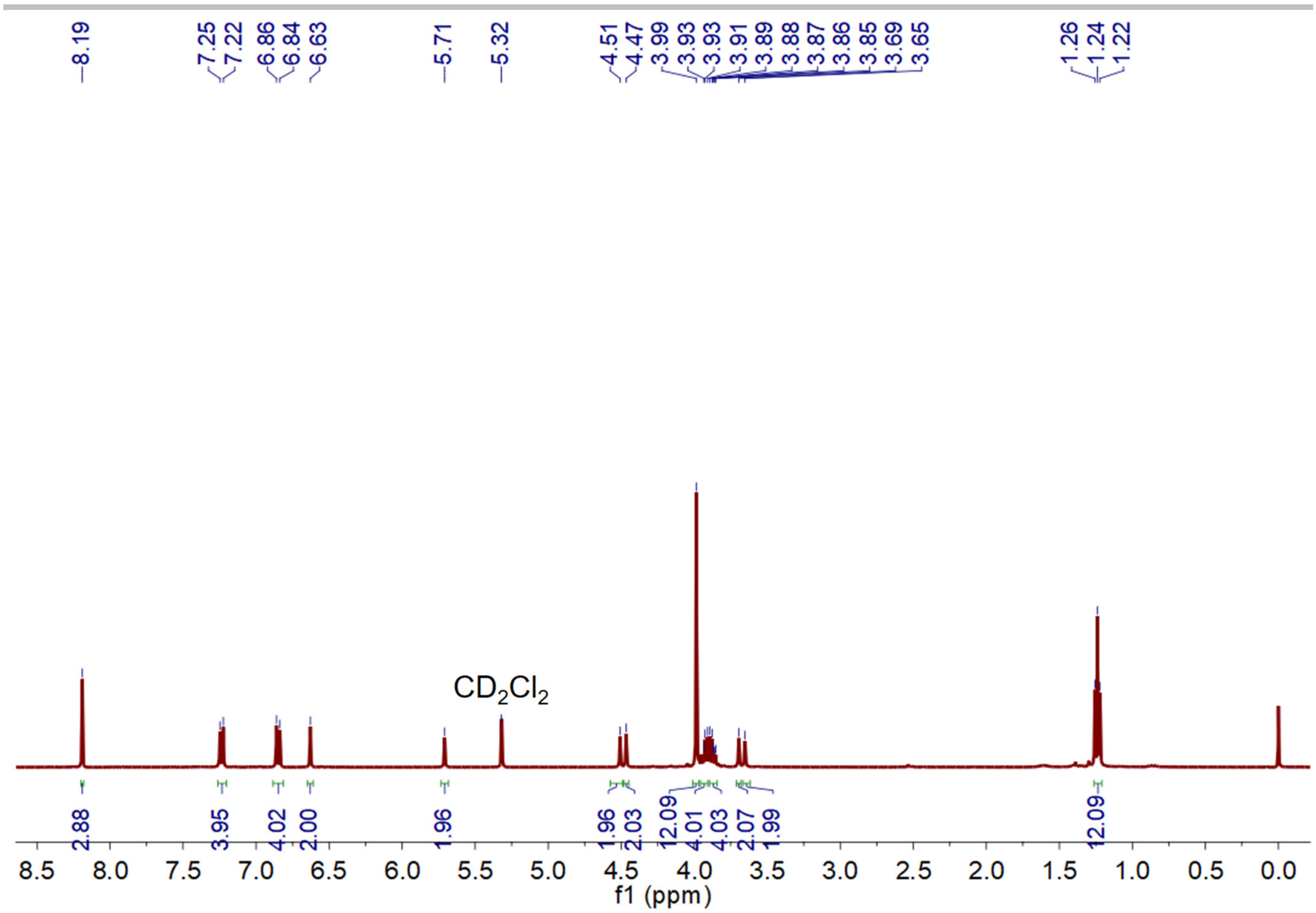

Supplementary Figure 33. ^{1}H NMR spectrum (500 MHz, CD_2Cl_2, 293 K) of H-TCNBα after adsorption of Cy vapor for 24 h.

^{1}H NMR experiments were performed by dissolving H-TCNBα after adsorption of single-component Tol/Py vapor in CD_2Cl_2. TGA profiles were recorded using H-TCNBα after adsorption of single-component Tol/Py vapor.

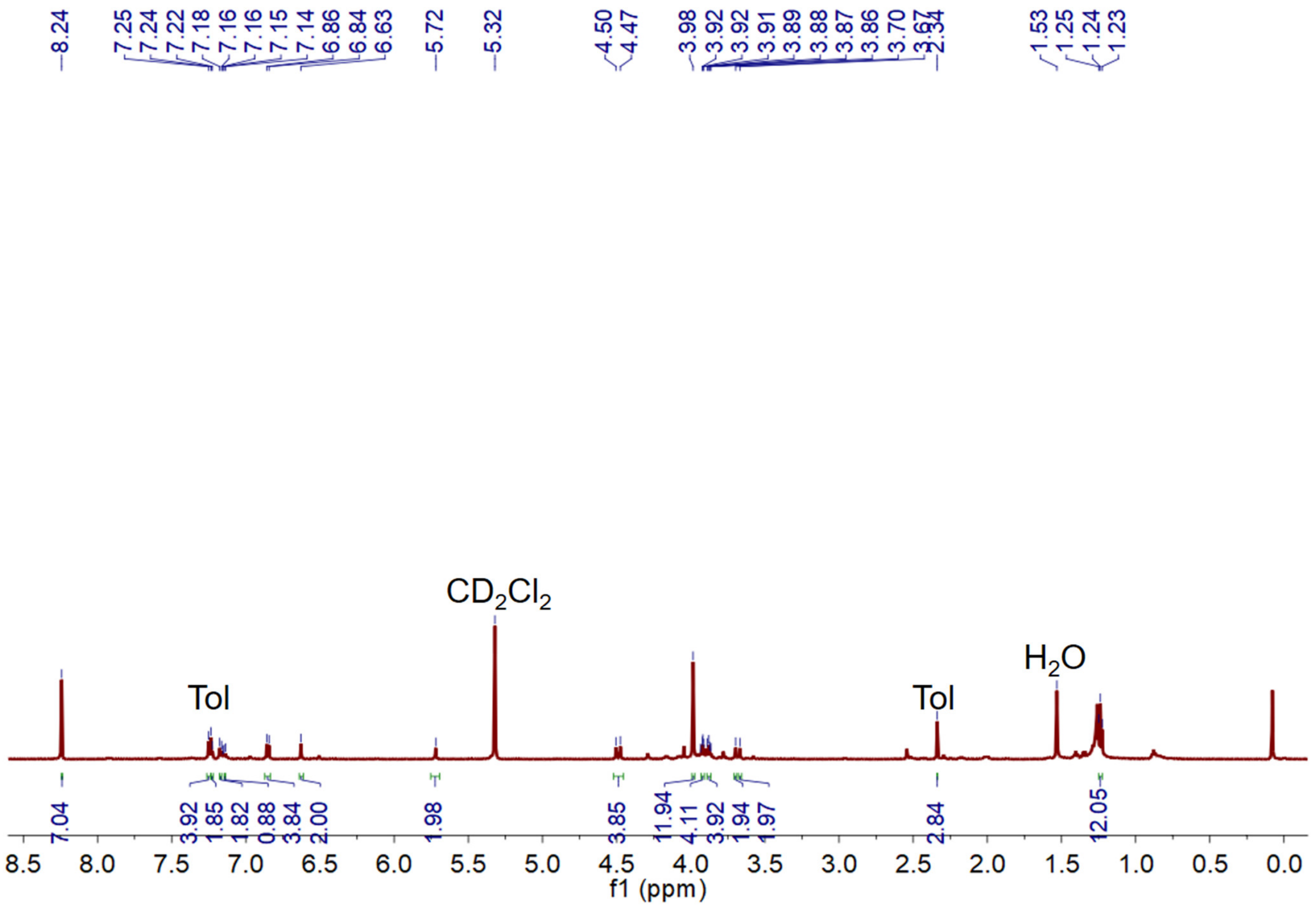

Supplementary Figure 34. ^{1}H NMR spectrum (500 MHz, CD_2Cl_2, 293 K) of H-TCNBα after adsorption of Tol vapor for 24 h.

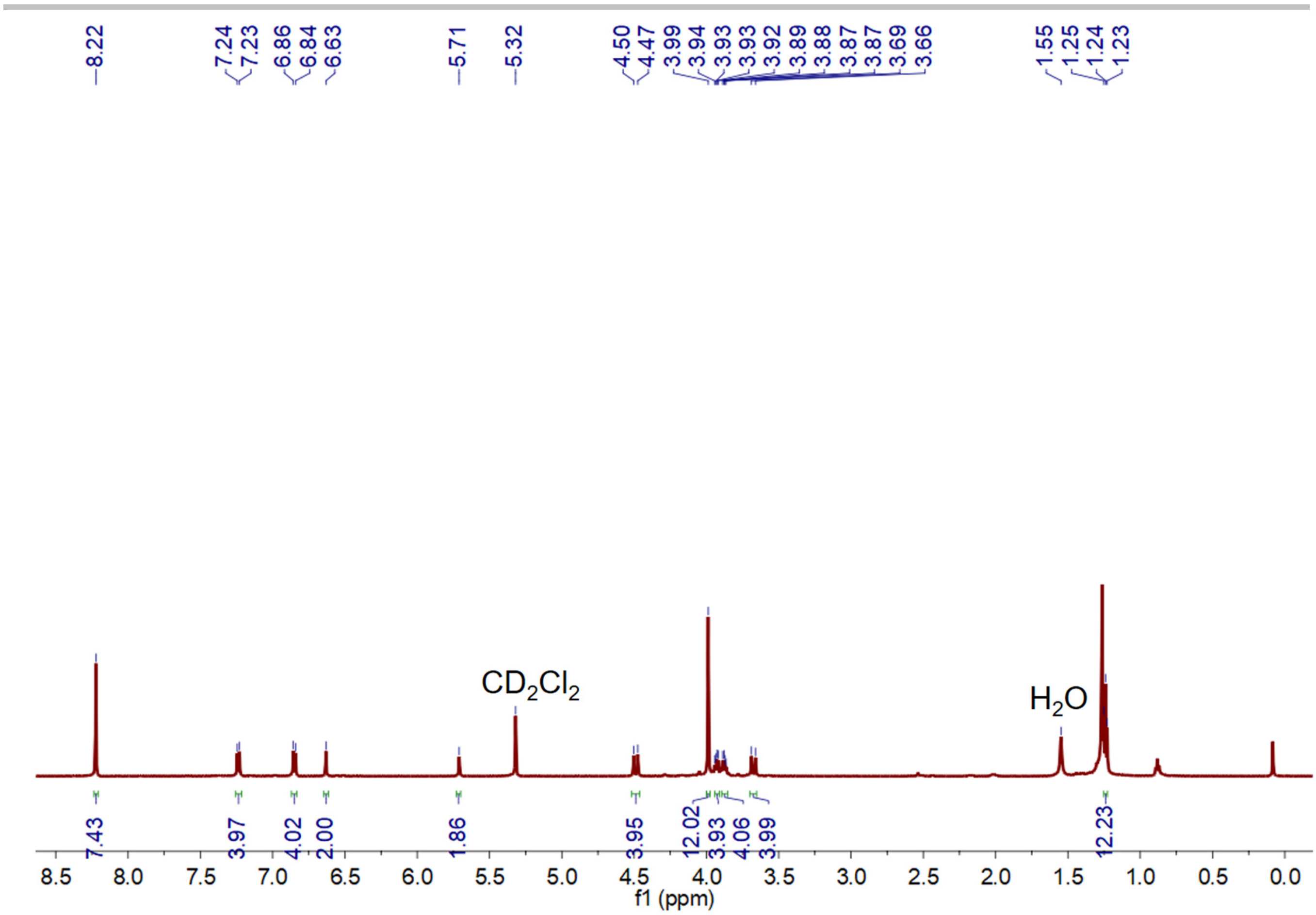

Supplementary Figure 35. ^{1}H NMR spectrum (500 MHz, CD_2Cl_2, 293 K) of H-TCNB*α* after adsorption of Py vapor for 24 h.

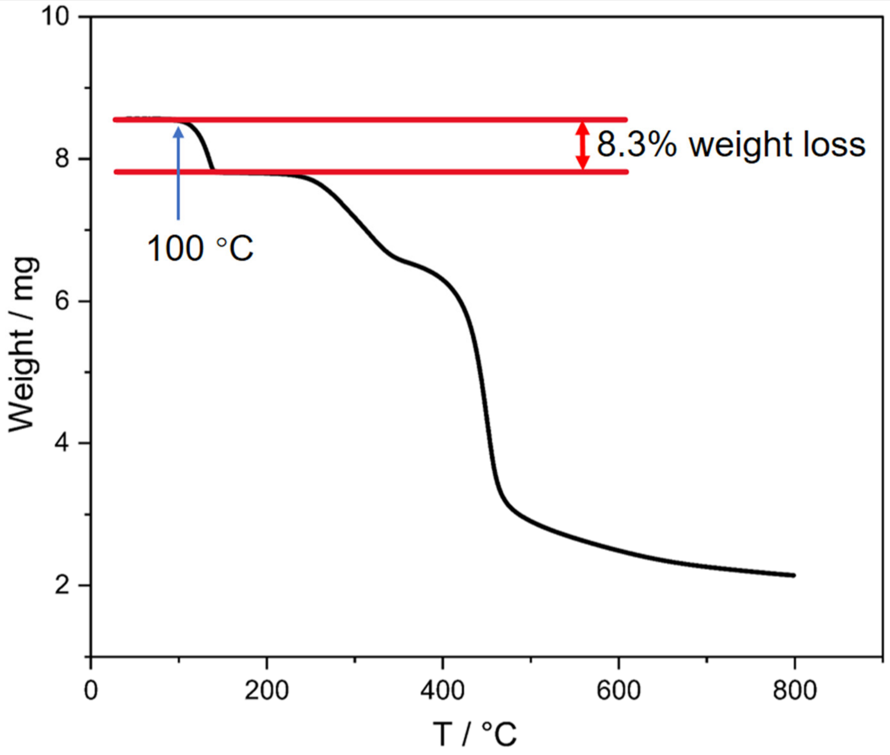

Supplementary Figure 36. Thermogravimetric analysis of H-TCNB*α* after adsorption of Bz vapor for 24 h. The weight loss below 200 °C can be calculated as one Bz molecule per H-TCNB molecule.

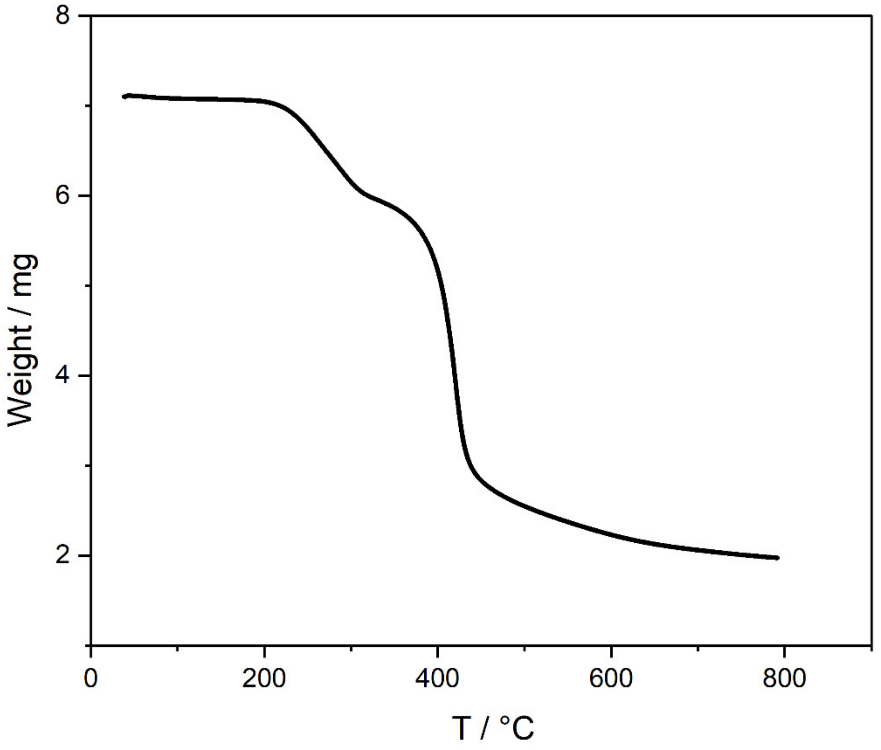

Supplementary Figure 37. Thermogravimetric analysis of H-TCNB*α* after adsorption of Cy vapor for 24 h.

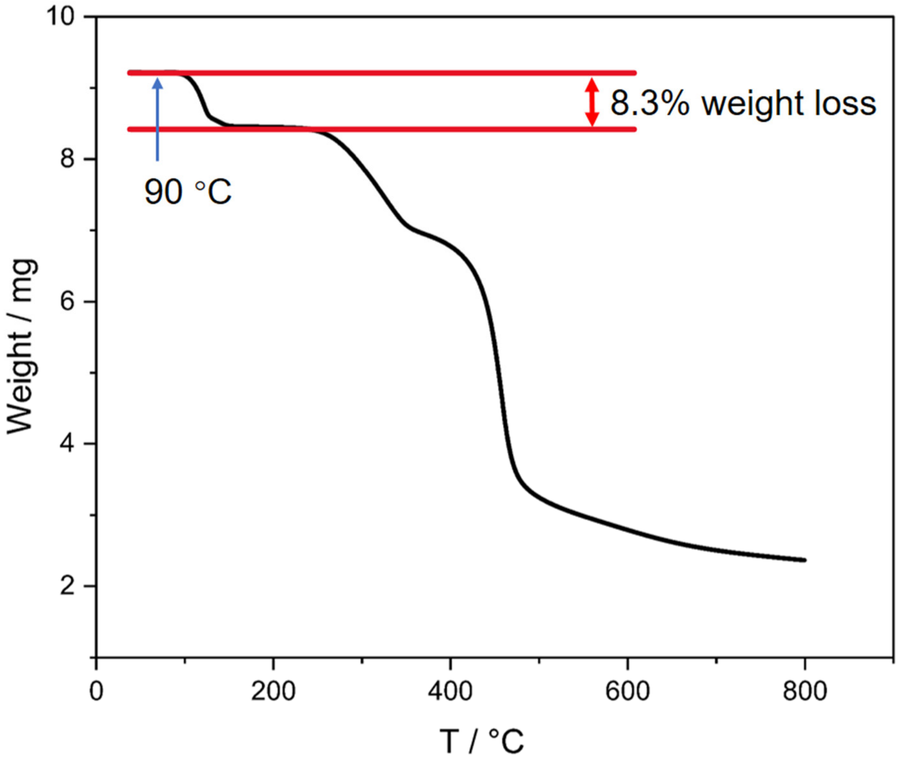

Supplementary Figure 38. Thermogravimetric analysis of H-TCNB*α* after adsorption of Tol vapor for 24 h. The weight loss below 150 °C can be calculated as 0.86 Tol molecule per H-TCNB molecule.

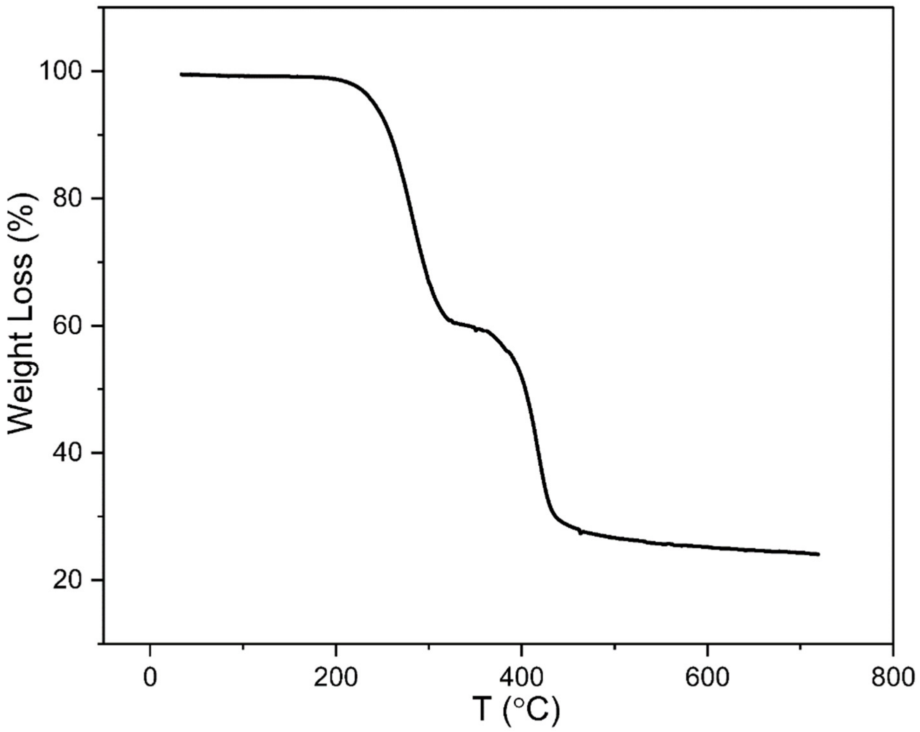

Supplementary Figure 39. Thermogravimetric analysis of H-TCNB*α* after adsorption of Py vapor for 24 h.

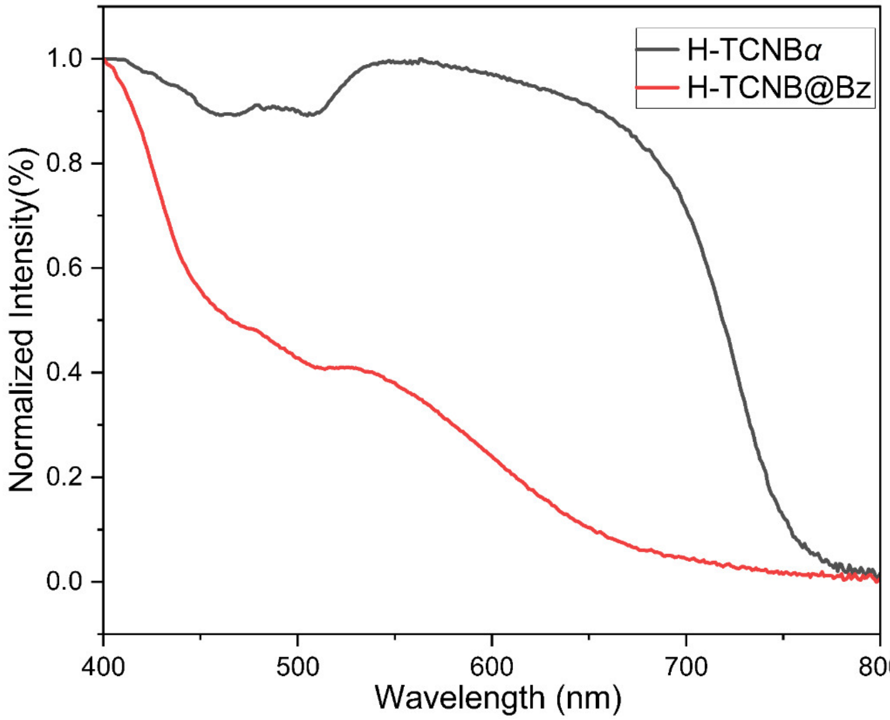

Supplementary Figure 40. Normalized diffuse reflectance spectra of H-TCNB*α* upon adsorption of Bz.

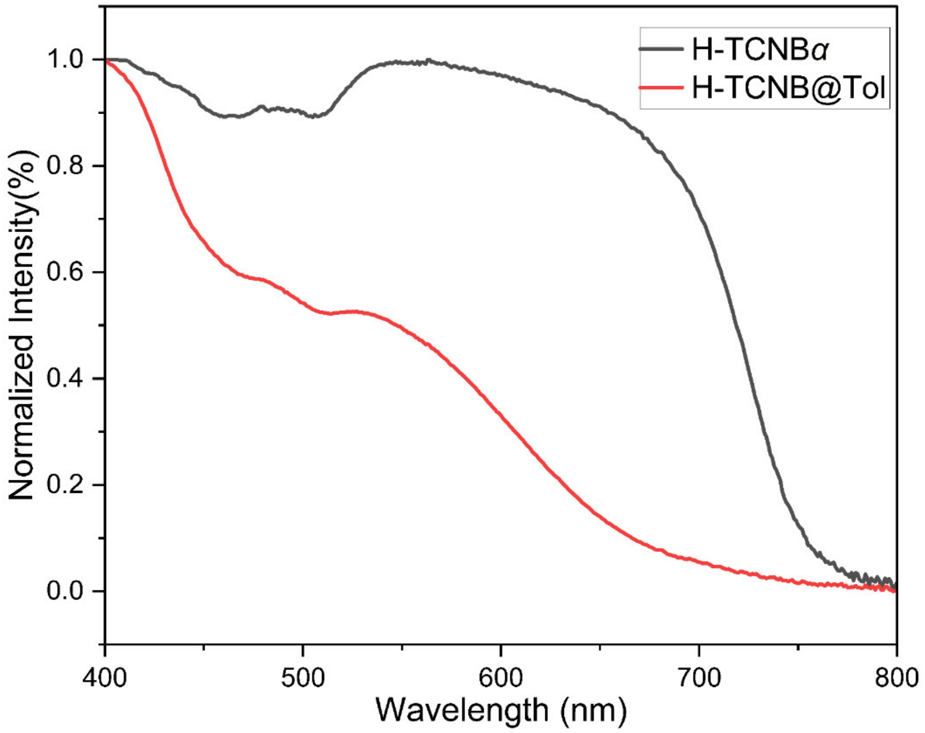

Supplementary Figure 41. Normalized diffuse reflectance spectra of H-TCNB*α* upon adsorption of Tol.

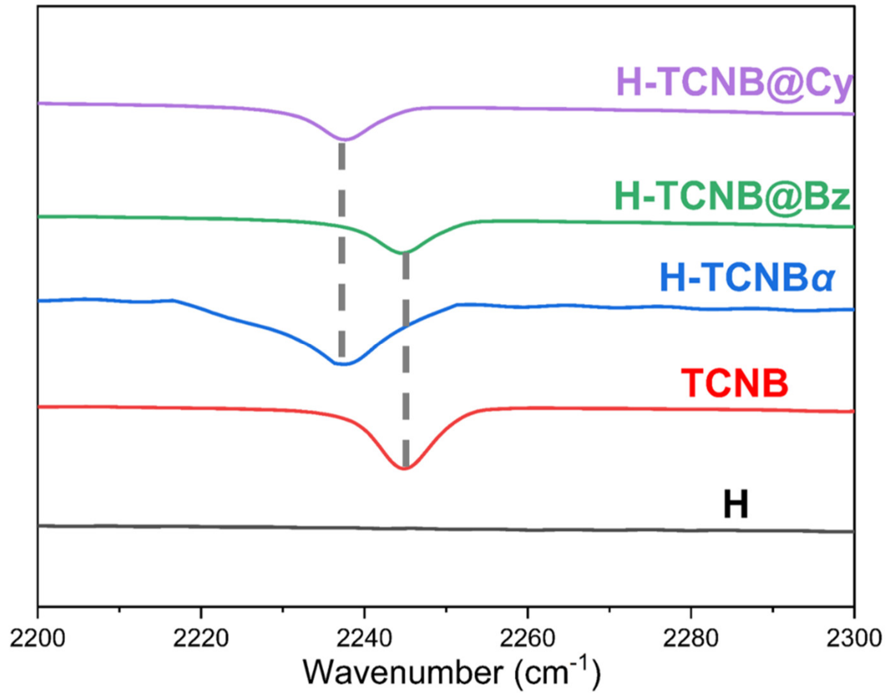

Supplementary Figure 42. Partial FT-IR spectra of H, TCNB, H-TCNB*α*, H-TCNB@Bz and H-TCNB@Cy.

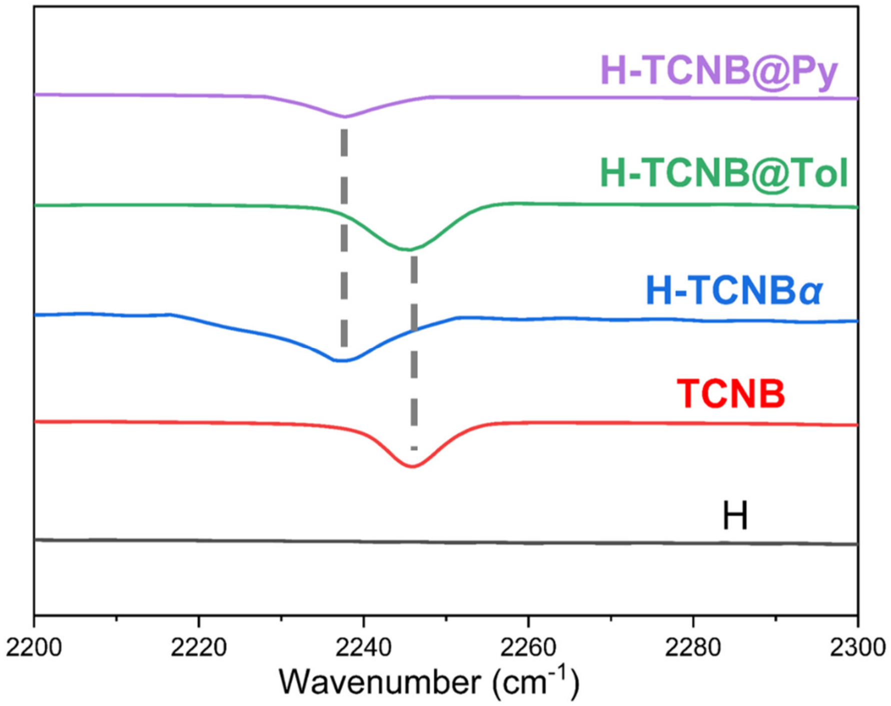

Supplementary Figure 43. Partial FT-IR spectra of H, TCNB, H-TCNB*α*, H-TCNB@Tol and H-TCNB@Py.

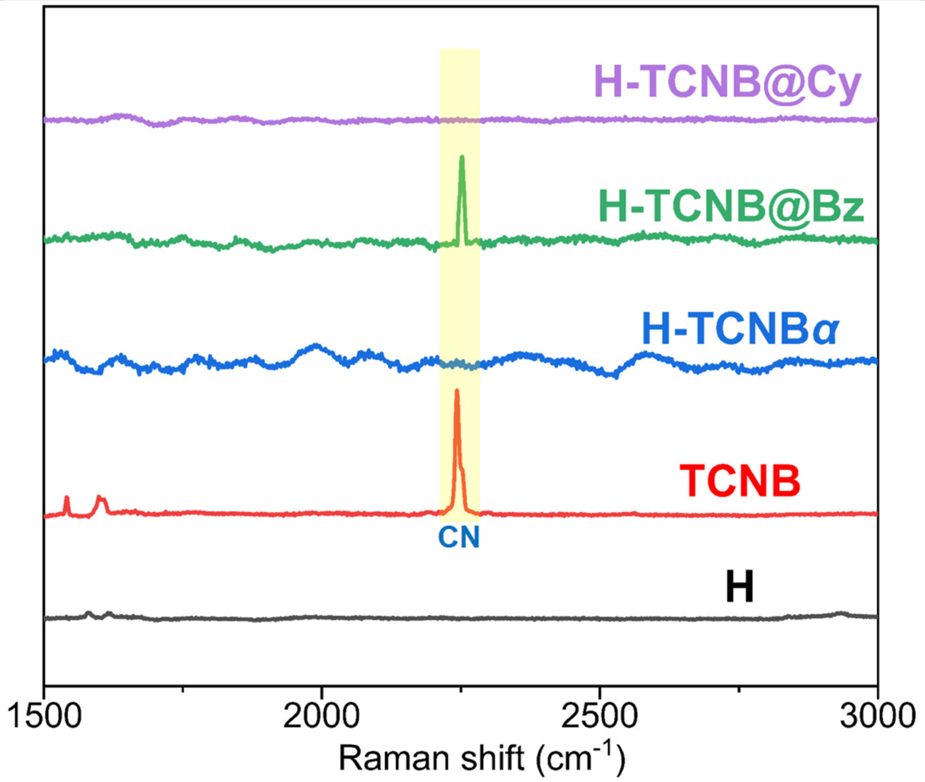

Supplementary Figure 44. Enlarged Raman spectra of H, TCNB, H-TCNB*α*, H-TCNB@Bz and H-TCNB@Cy.

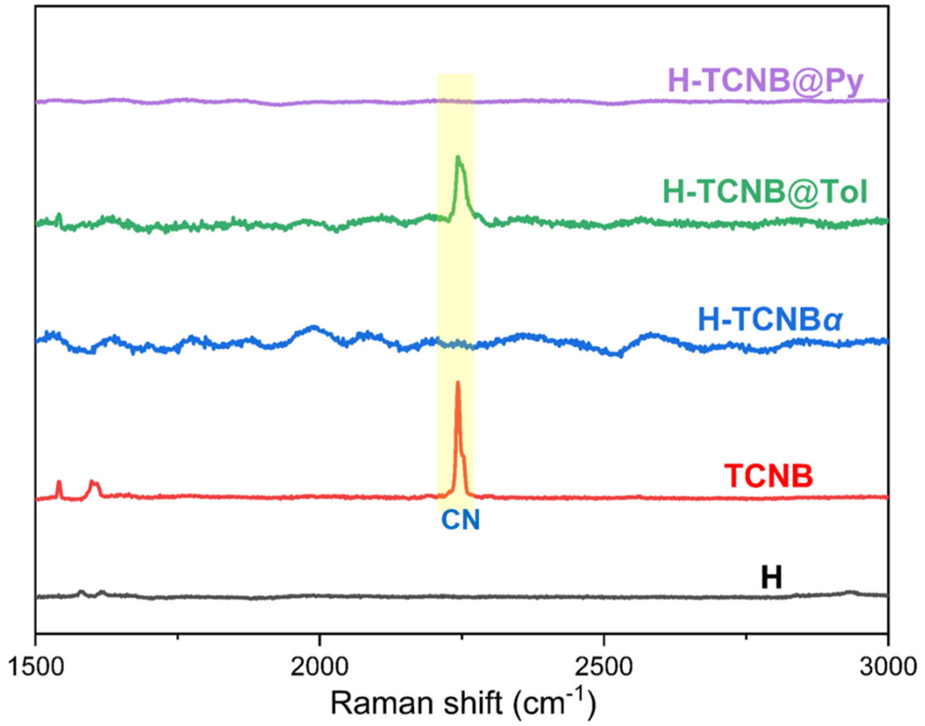

Supplementary Figure 45. Enlarged Raman spectra of H, TCNB, H-TCNB*α*, H-TCNB@Tol and H-TCNB@Py.

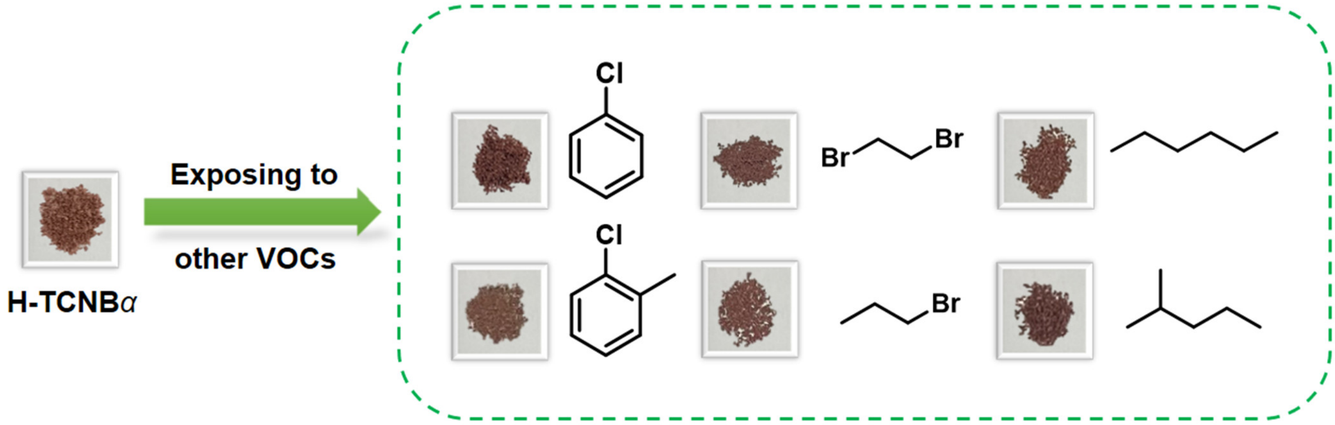

Supplementary Figure 46. The color of H-TCNB*α* after exposure to other VOCs.

2.7. Non-covalent interaction analysis in single crystal structure of H-TCNB@Bz

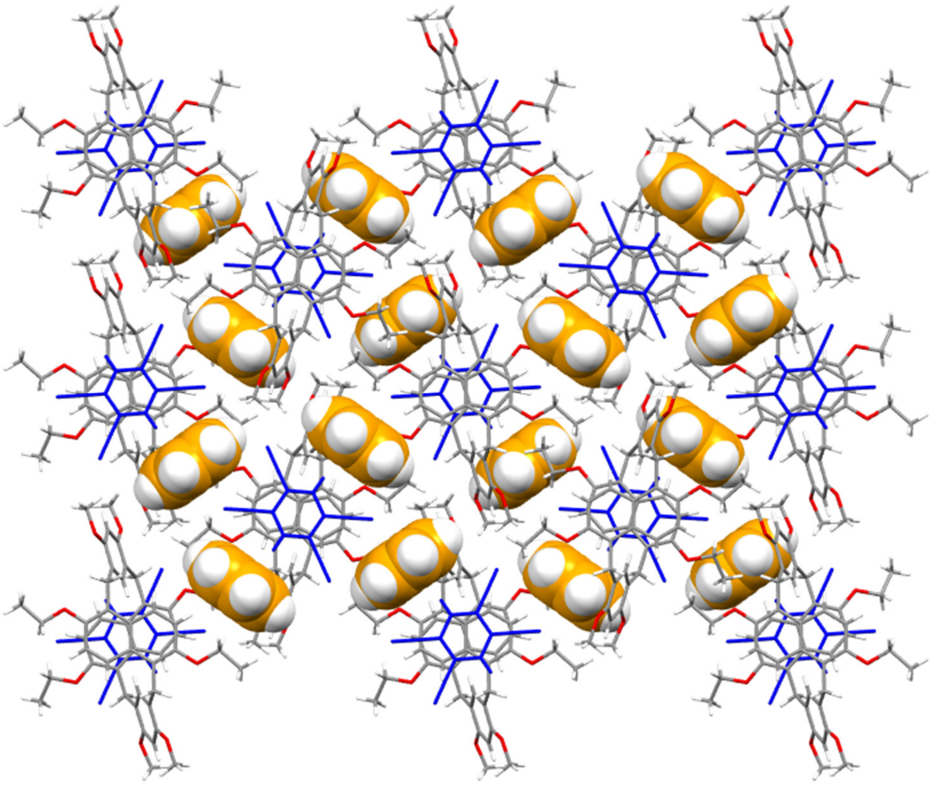

Supplementary Figure 47. Illustration of the packing model of H-TCNB@Bz.

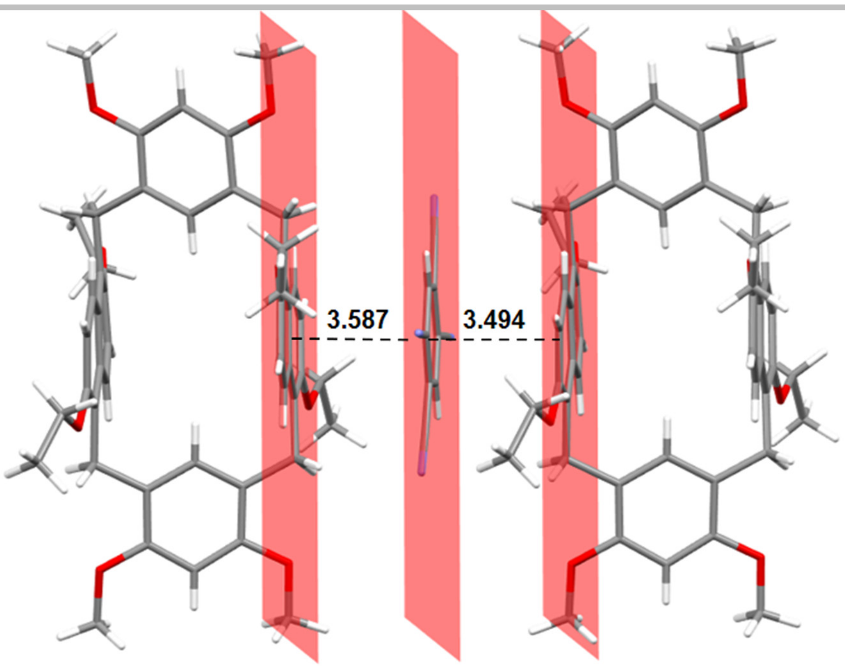

Supplementary Figure 48. The exo-wall π–π interactions between H and TCNB in H-TCNB@Bz. The Bz molecules are omitted for clarity. π–π distances: 3.587 Å and 3.494 Å.

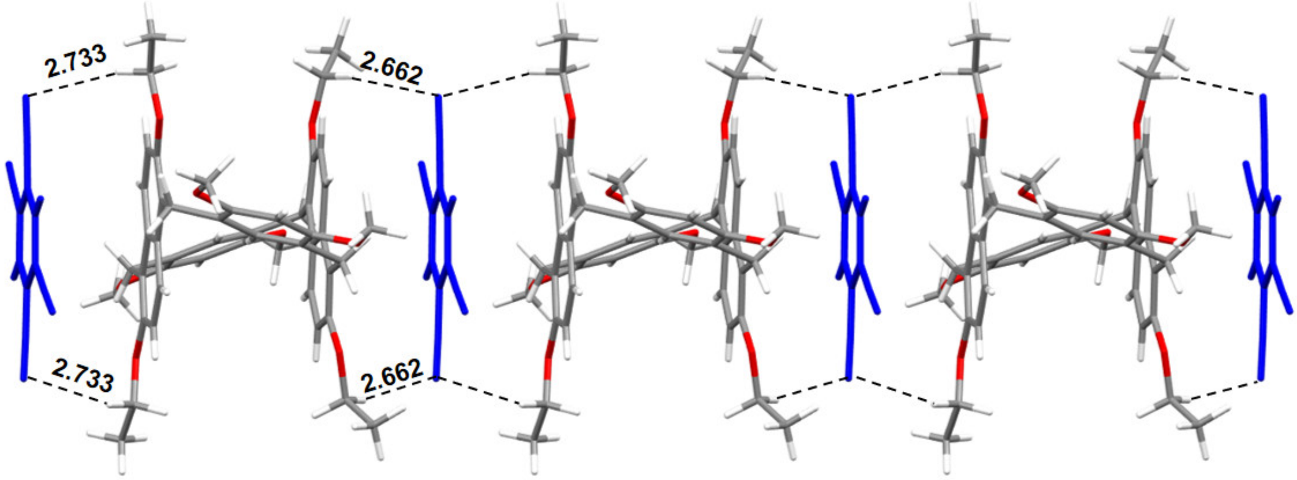

Supplementary Figure 49. The exo-wall C–H···N interaction between H and TCNB in H-TCNB@Bz. The Bz molecules are omitted for clarity. C–H···N distances: 2.733 Å and 2.662 Å.

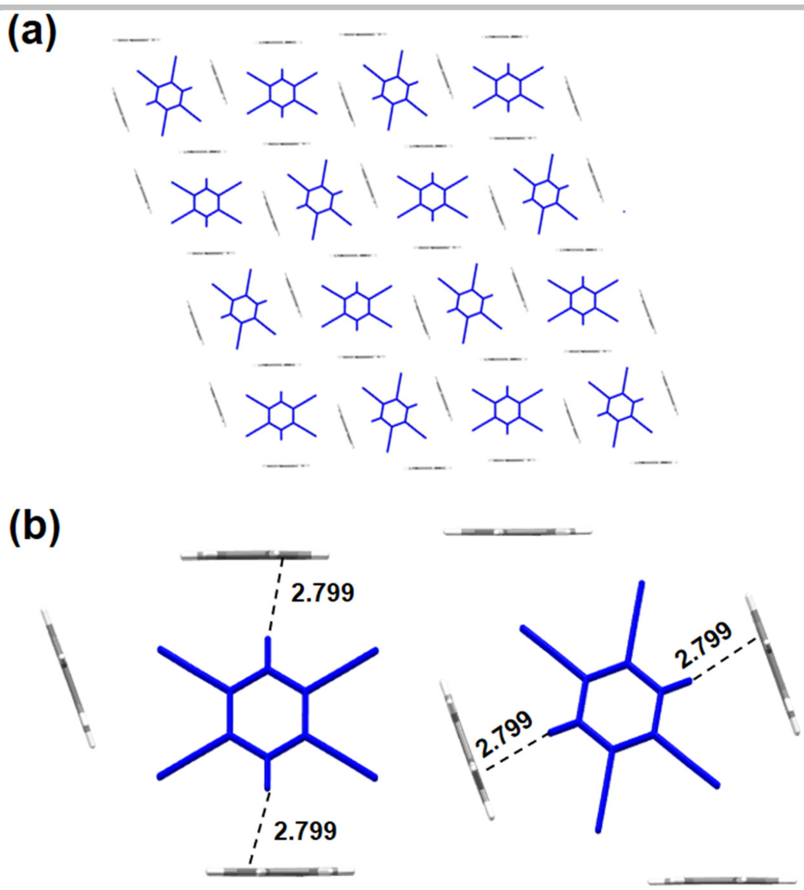

Supplementary Figure 50. Illustration of the (a) packing model of H-TCNB@Bz and (b) C–H···π interaction between Bz and TCNB. The H molecules are omitted for clarity. C–H···π distance: 2.799 Å.

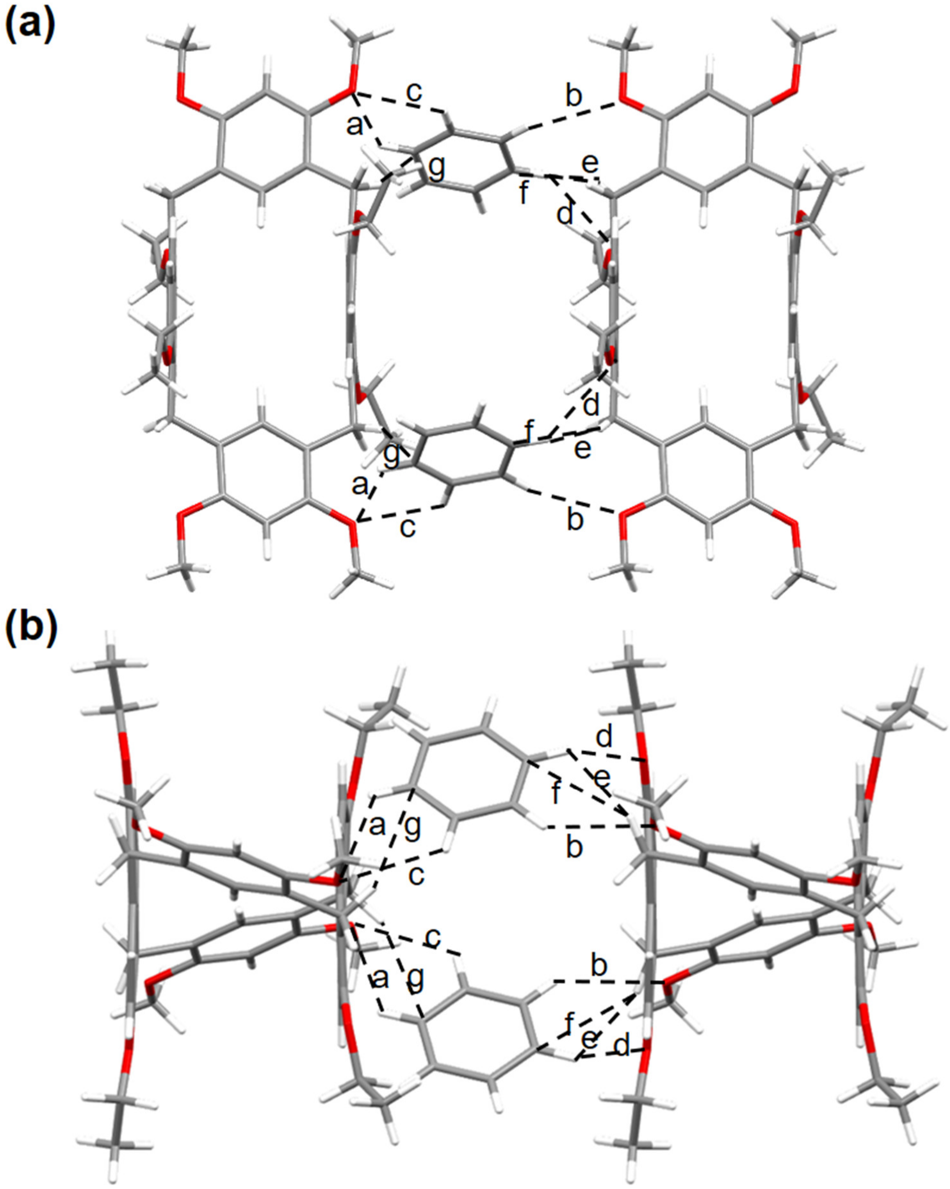

Supplementary Figure 51. Illustration of C–H···O and C–H···π interactions between Bz and H. The TCNB molecules are omitted for clarity. C–H···O distances: a = 2.707 Å, b = 2.685 Å, c = 2.669 Å. C–H···π distances: d = 2.685 Å, e = 2.234 Å, f = 2.800 Å, g = 2.804 Å.

2.8. Selectivity experiments of H-TCNB*α* for the mixture vapors of Bz and Cy

An open 5 mL vial containing 2 mg of H-TCNB*α* adsorbent was placed in a sealed 20 mL vial containing 1 mL of a Bz/Cy mixture (*v*:*v* = 1:1). Uptake in H-TCNB*α* was measured hour by hour by completely dissolving the crystals and measuring the ratio of Bz or Cy to H-TCNB by ^{1}H NMR. The relative uptakes of Bz and Cy in H*α* were also measured by heating the crystals to release the adsorbed vapor and detecting the relative amounts of Bz and Cy in the released vapor using headspace gas chromatography.

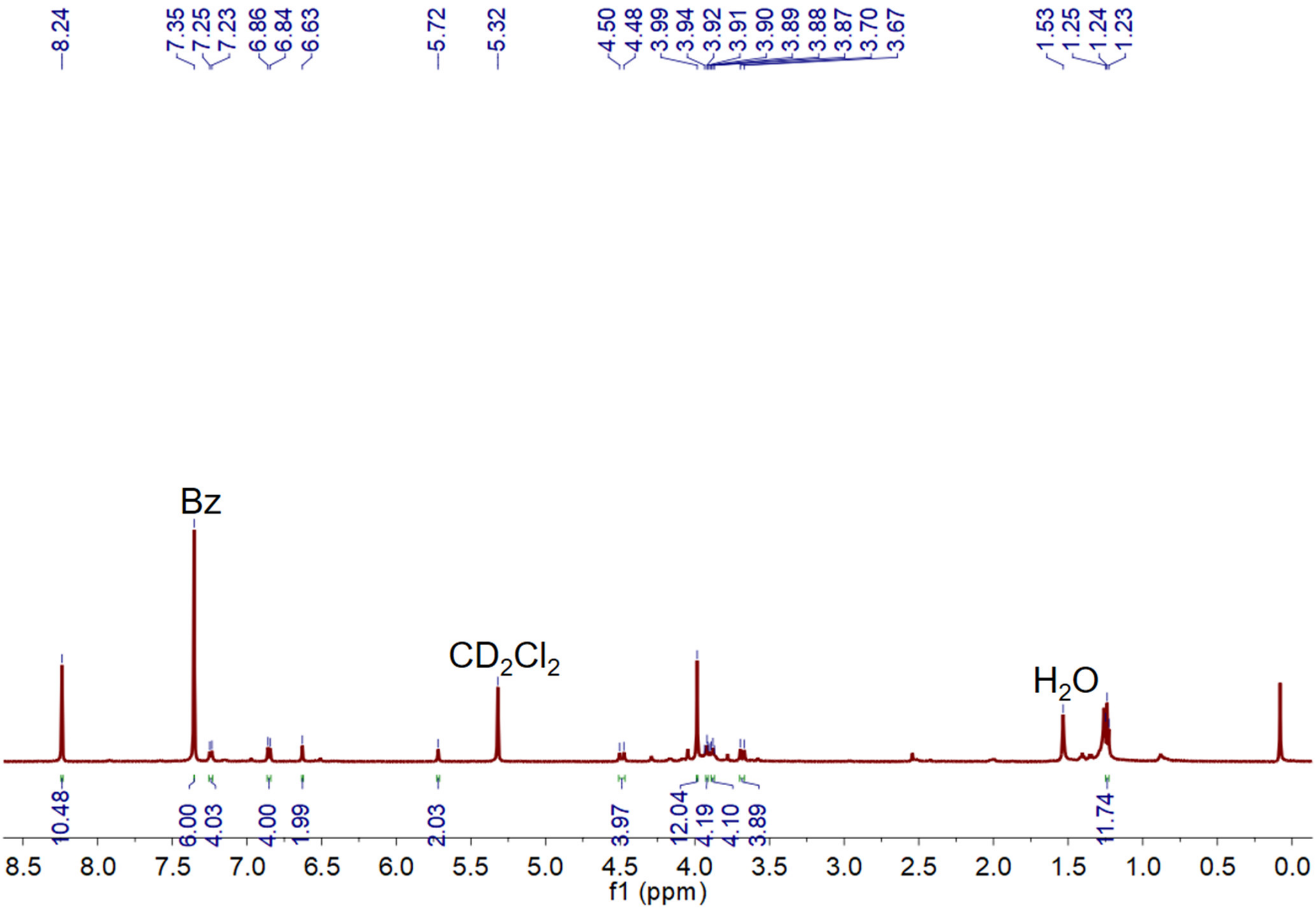

Supplementary Figure 52. ^{1}H NMR spectrum (500 MHz, CD_2Cl_2, 293 K) of H-TCNB*α* after adsorption of a Bz/Cy mixture (*v*:*v* = 1:1) vapor for 12 h.

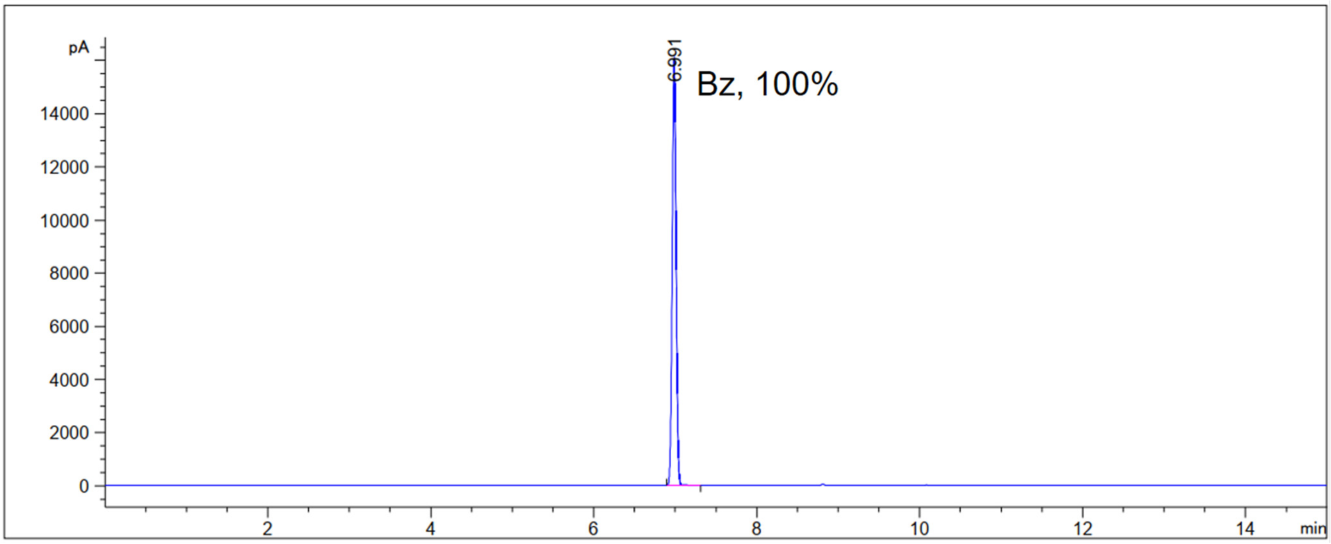

Supplementary Figure 53. Relative uptakes of Bz and Cy adsorbed in H-TCNB*α* for 24 h using head space gas chromatography.

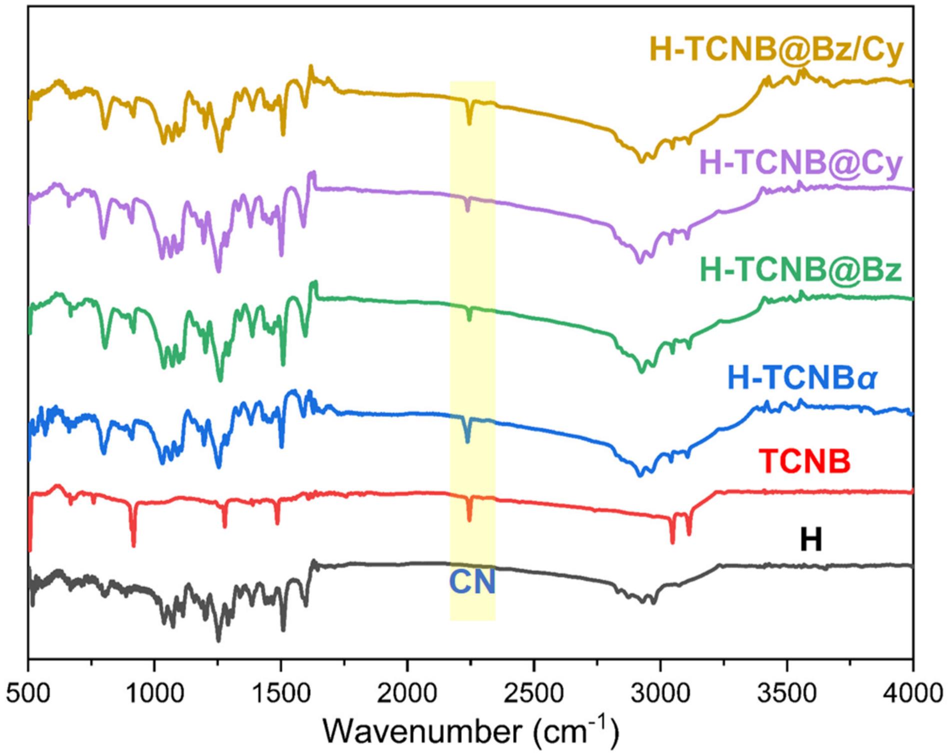

Supplementary Figure 54. FT-IR spectra of H, TCNB, H-TCNB*α*, H-TCNB@Bz, H-TCNB@Cy, and H-TCNB@Bz/Cy.

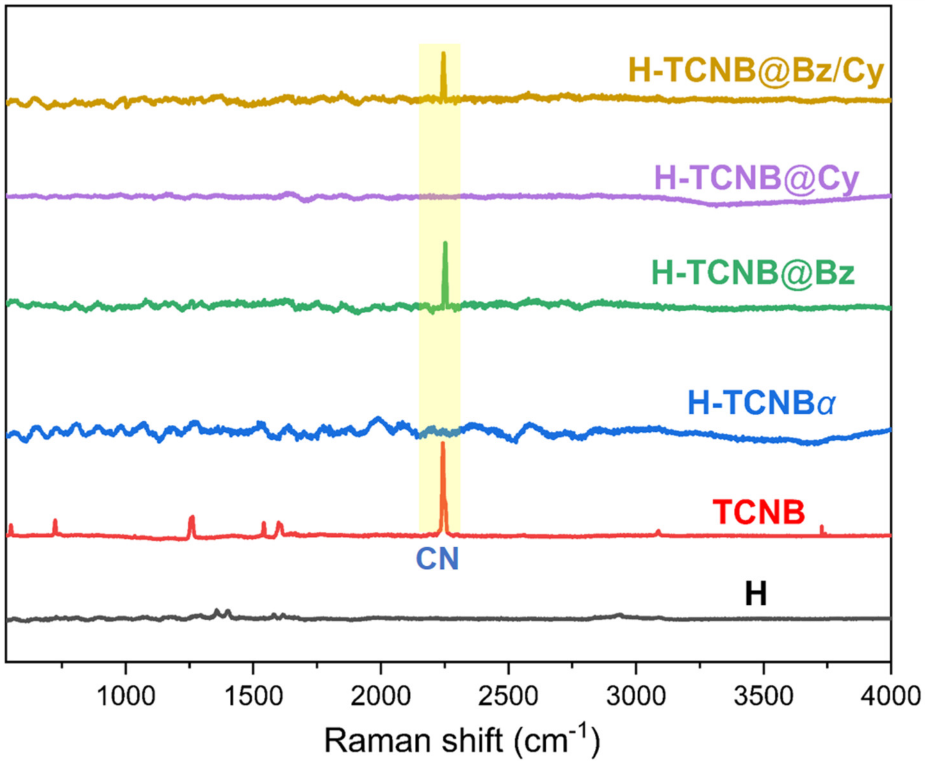

Supplementary Figure 55. Raman spectra of H, TCNB, H-TCNB*α*, H-TCNB@Bz, H-TCNB@Cy, and H-TCNB@Bz/Cy.

2.9. Selectivity experiments of H-TCNB*α* for the mixture vapors of Tol and Py

An open 5 mL vial containing 2 mg of H-TCNB*α* adsorbent was placed in a sealed 20 mL vial containing 1 mL of a Tol/Py mixture (*v*:*v* = 1:1). Uptake in H-TCNB*α* was measured hour by hour by completely dissolving the crystals and measuring the ratio of Tol or Py to H-TCNB*α* by ^{1}H NMR. The relative uptakes of Tol and Py in H*α* were also measured by heating the crystals to release the adsorbed vapor and detecting the relative amounts of Tol and Py in the released vapor using headspace gas chromatography.

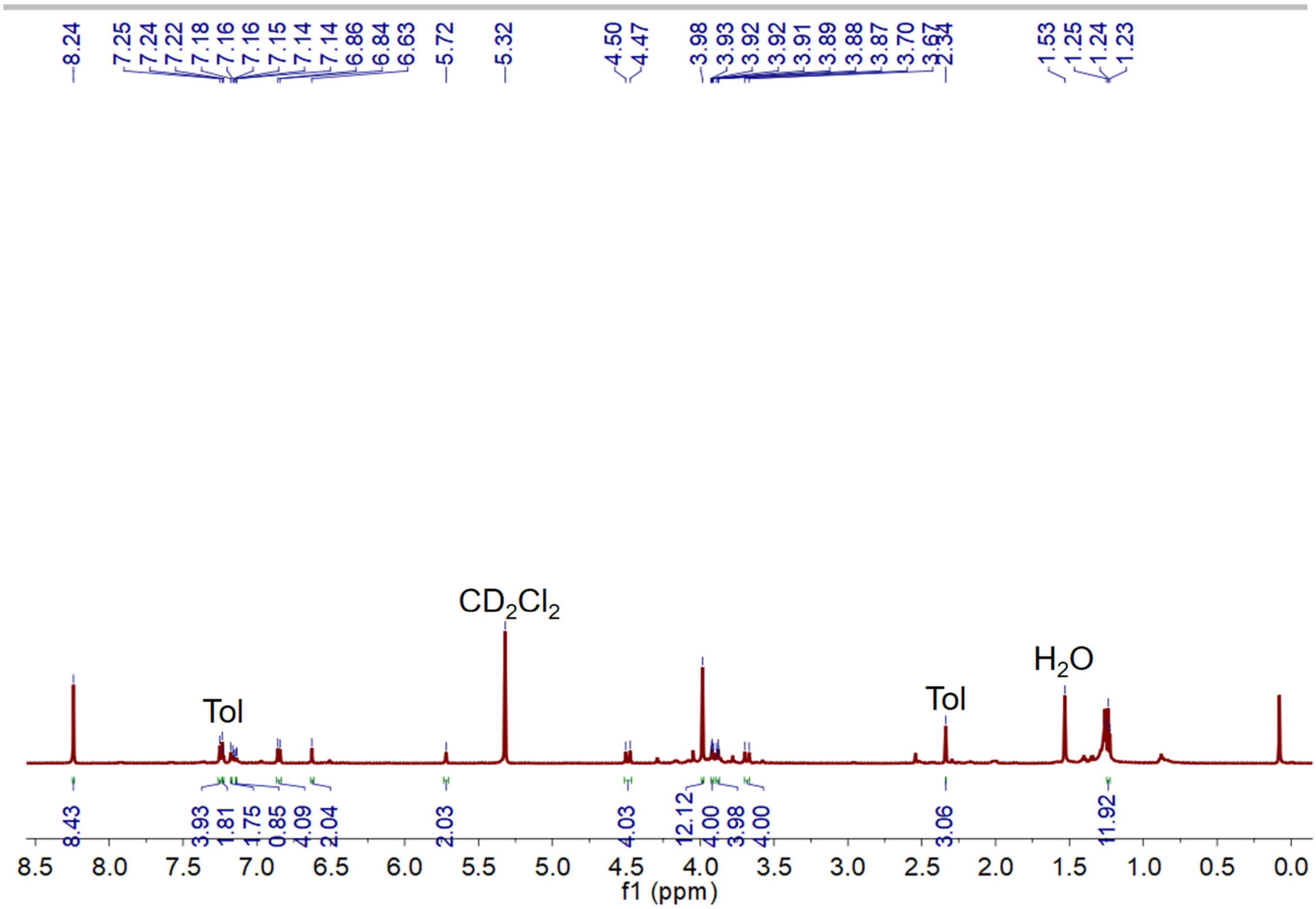

Supplementary Figure 56. ¹H NMR spectrum (500 MHz, CD_2Cl_2, 293 K) of H*α* after adsorption of a Tol/Py mixture (*v*:*v* = 1:1) vapor for 24 h.

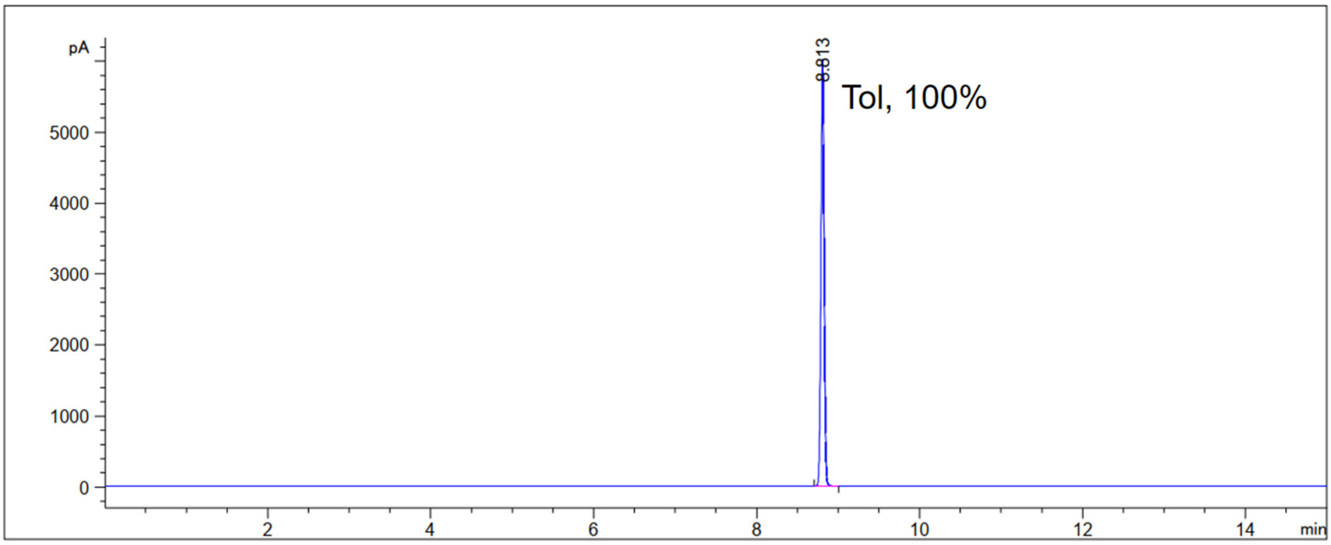

Supplementary Figure 57. Relative uptakes of Tol and Py adsorbed in H-TCNB*α* for 24 h using head space gas chromatography.

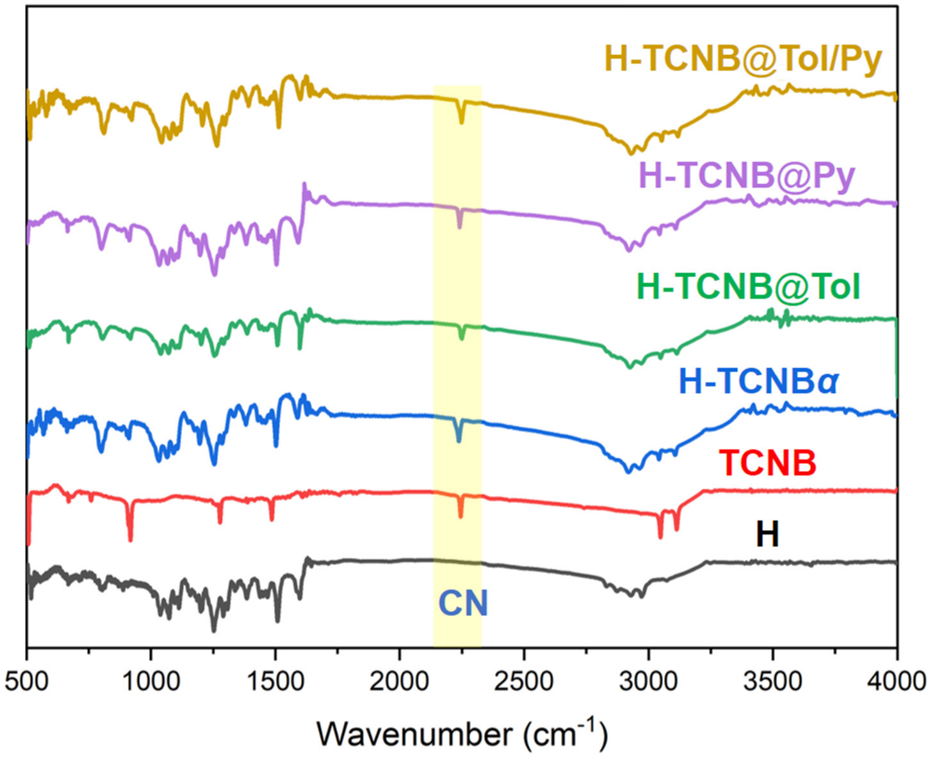

Supplementary Figure 58. FT-IR spectra of H, TCNB, H-TCNB*α*, H-TCNB@Tol, H-TCNB@Py, and H-TCNB@Tol/Py.

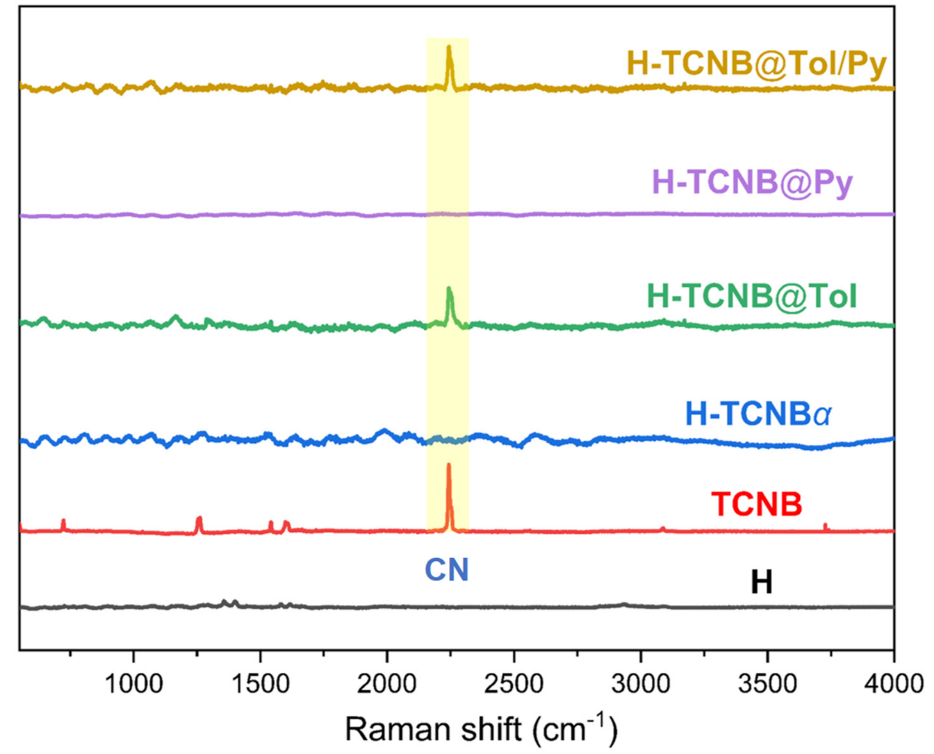

Supplementary Figure 59. Raman spectra of H, TCNB, H-TCNB*α*, H-TCNB@Tol, H-TCNB@Py, and H-TCNB@Tol/Py.

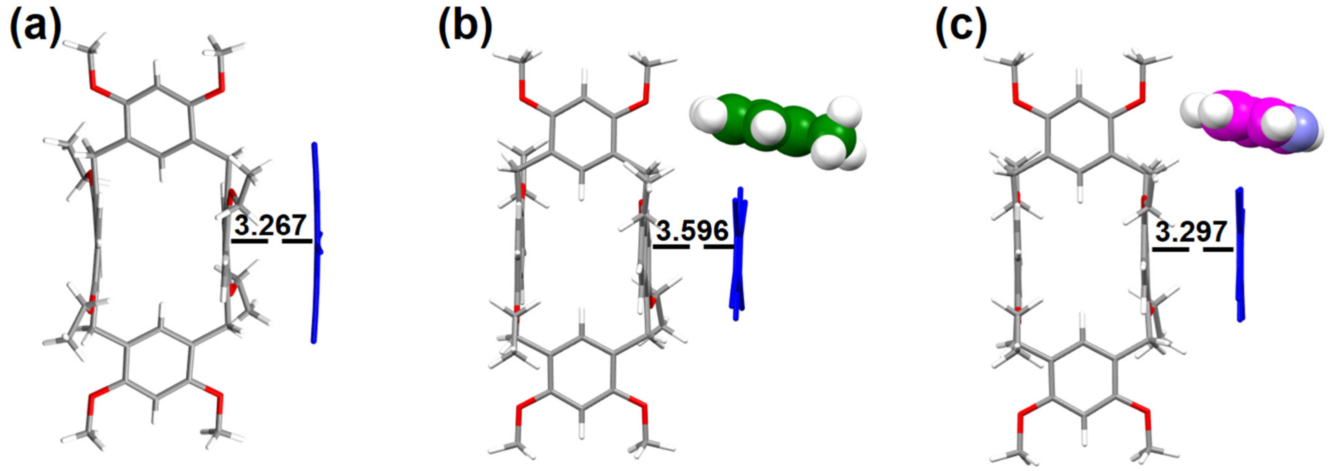

Supplementary Figure 60. The centroid-centroid distance of H with TCNB in (a) H-TCNBα (3.267 Å) and calculated binding modes of (b) H-TCNB@Tol (3.596 Å) and (c) H-TCNB@Py (3.587 Å).

Supplementary Table 4. Thermodynamic parameters of calculated binding modes of H-TCNB@Tol and H-TCNB@Py at 298 K.

	ΔH (kJ/mol)	ΔG (kJ/mol)
H-TCNB@Tol	-43.33	-38.33
H-TCNB@Py	20.16	12.20

2.10. Purity experiments of liquid Cy or Py by H-TCNBα

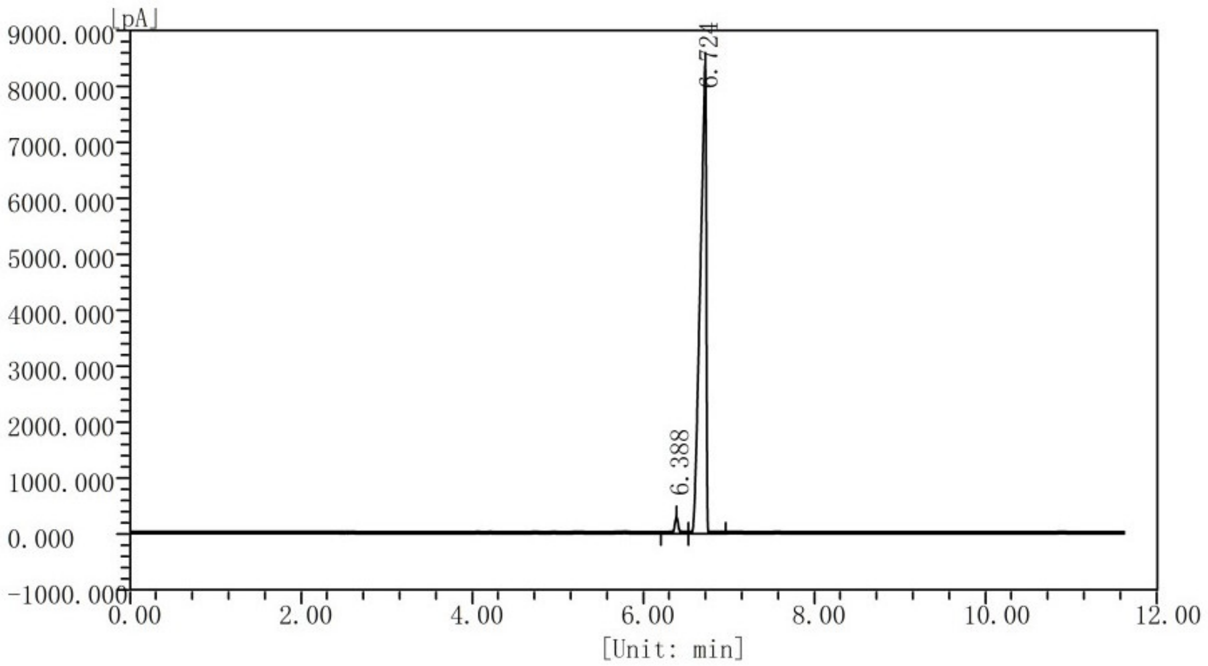

Analysis Report

Peak	Component	RetTime [min]	Height [uV]	Area [uV*s]	Area [%]	Content [%]
1	**Bz**	6.388	291542.0	824061.4	1.9910	1.9910
2	**Cy**	6.724	8397338.9	40565863.0	98.0090	98.0090
		Totals:	8688881.0	41389924.0	100.0000	100.0000

Supplementary Figure 61. GC analysis of the sample of 98:2 Cy/Bz.

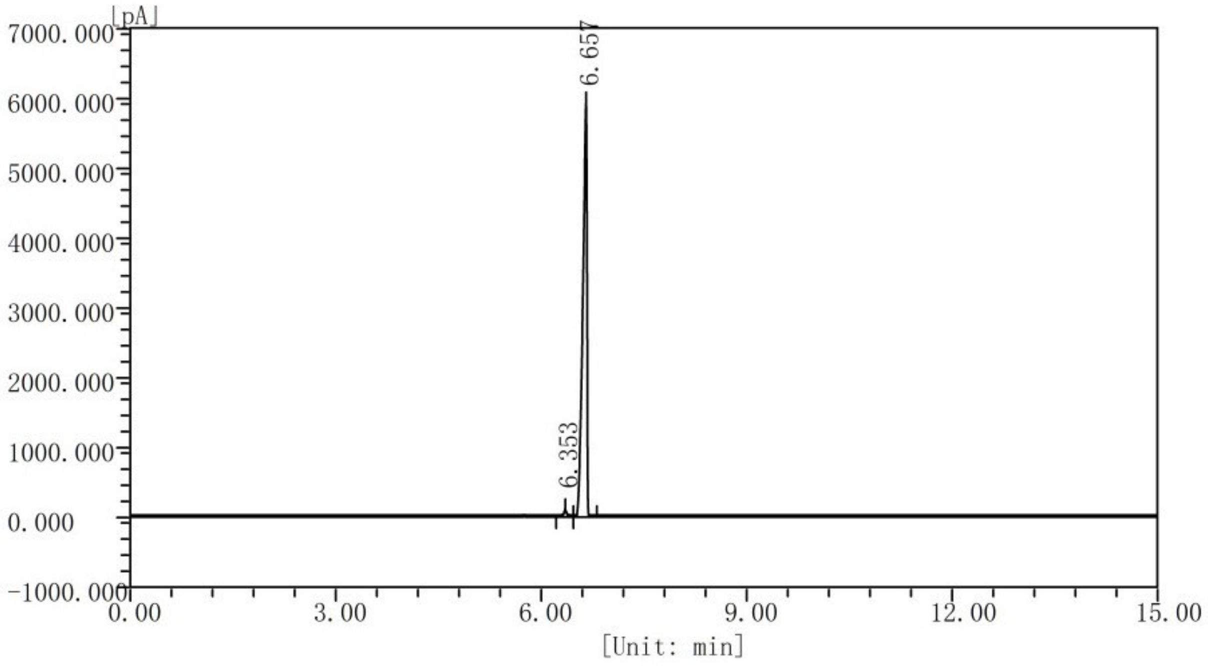

Analysis Report

Peak	Component	RetTime [min]	Height [uV]	Area [uV*s]	Area [%]	Content [%]
1	**Bz**	6.353	99565.2	301520.9	1.2052	1.2052
2	**Cy**	6.657	5925516.1	24715940.6	98.7948	98.7948
		Totals:	6025081.0	25017462.0	100.0000	100.0000

Supplementary Figure 62. GC analysis of the 98:2 Cy/Bz after adsorption by H-TCNB*α* (3.00 mg).

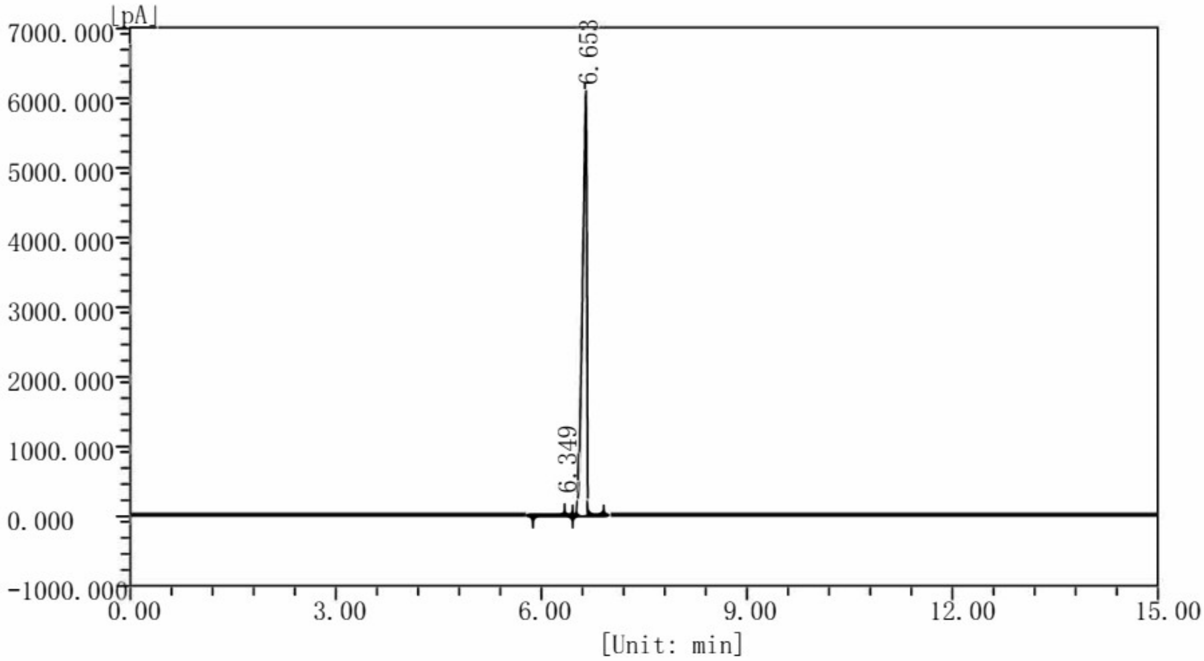

Analysis Report

Peak	Component	RetTime [min]	Height [uV]	Area [uV*s]	Area [%]	Content [%]
1	**Bz**	6.349	63596.9	222584.7	0.5680	0.5680
2	**Cy**	6.653	8940172.8	38964739.9	99.4320	99.4320
		Totals:	9003769.7	39187324.6	100.0000	100.0000

Supplementary Figure 63. GC analysis of the 98:2 Cy/Bz after adsorption by H-TCNB*α* (6.00 mg).

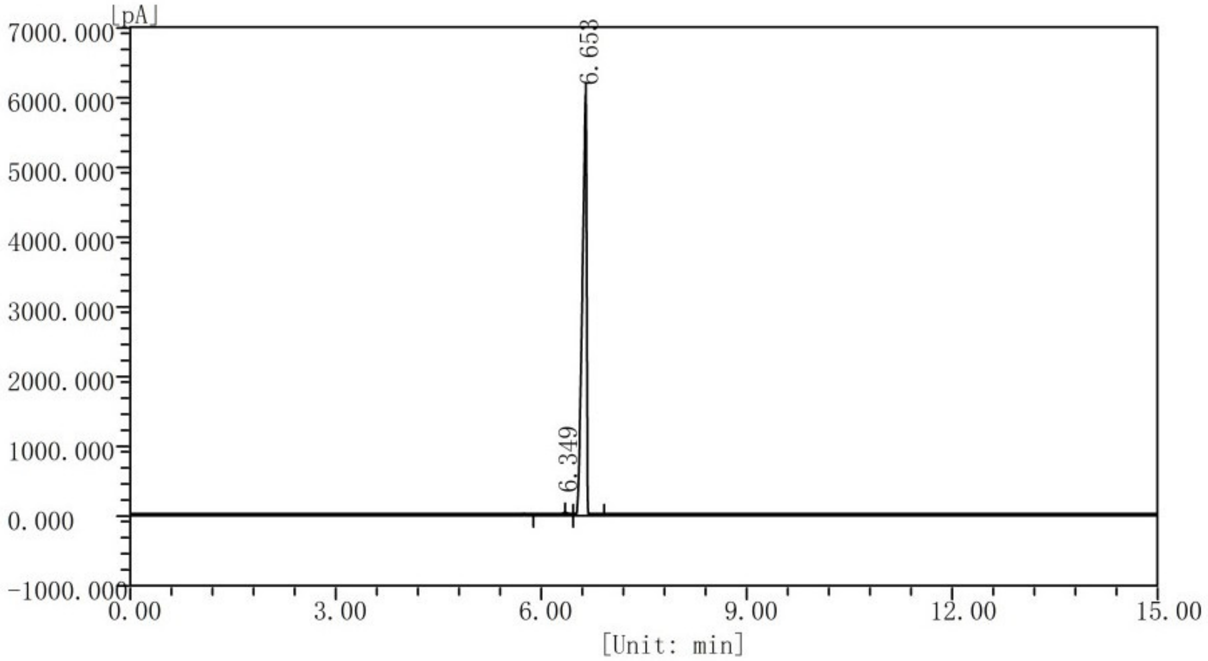

Analysis Report

Peak	Component	RetTime [min]	Height [uV]	Area [uV*s]	Area [%]	Content [%]
1	**Bz**	6.349	20552.9	71933.7	0.2720	0.2720
2	**Cy**	6.653	6050902.8	26372177.1	99.7280	99.7280
		Totals:	6071455.5	26444110.0	100.0000	100.0000

Supplementary Figure 64. GC analysis of the 98:2 Cy/Bz after adsorption by H-TCNB*α* (9.00 mg).

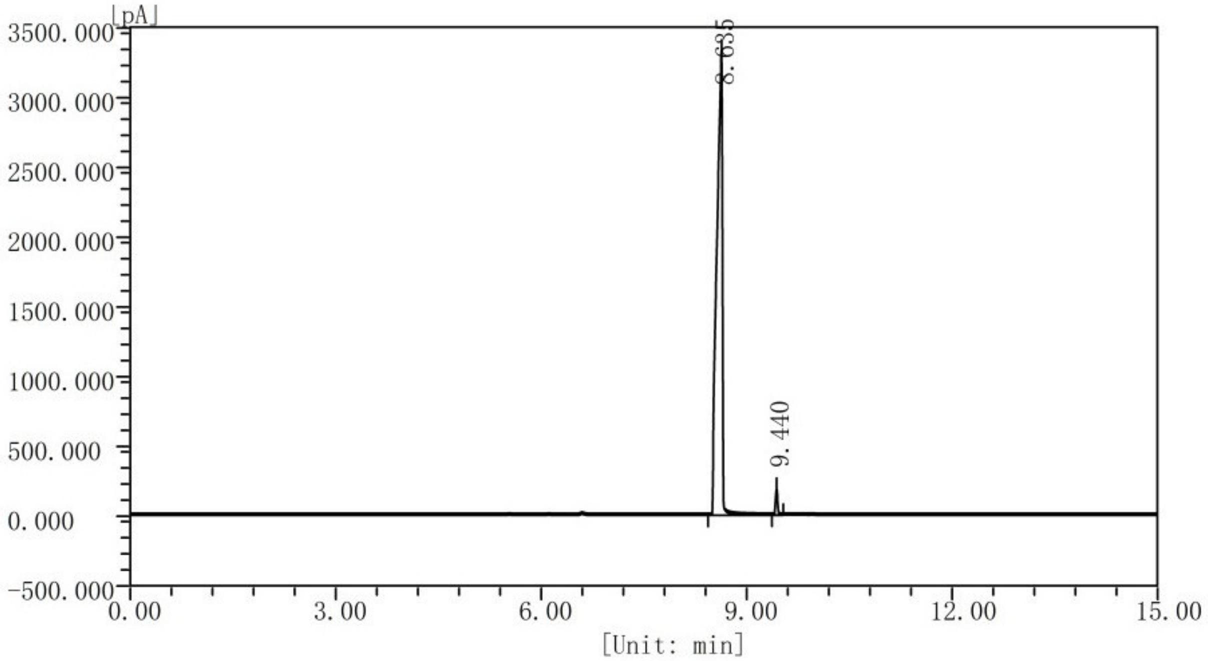

Analysis Report

Peak	Component	RetTime [min]	Height [uV]	Area [uV*s]	Area [%]	Content [%]
1	**Py**	8.635	3330020.8	18631926.3	97.9385	97.9385
2	**Tol**	9.440	186273.1	392189.2	2.0615	2.0615
		Totals:	3516294.0	19024116.0	100.0000	100.0000

Supplementary Figure 65. GC analysis of 98:2 Py/Tol.

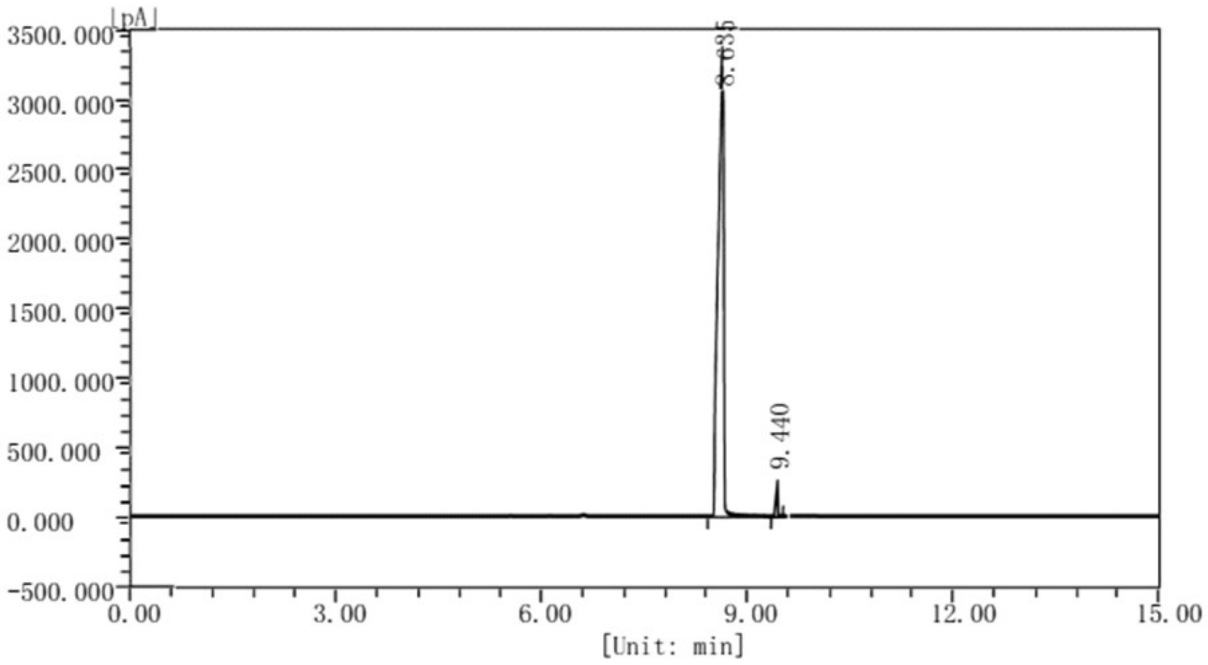

Analysis Report

Peak	Component	RetTime [min]	Height [uV]	Area [uV*s]	Area [%]	Content [%]
1	**Py**	8.635	3870305.7	21654895.2	98.4877	98.4877
2	**Tol**	9.440	164584.2	346524.3	1.5123	1.5123
		Totals:	4034890.0	22001420.0	100.0000	100.0000

Supplementary Figure 66. GC analysis of the 98:2 Py/Tol after adsorption by H-TCNB*α* (3.00 mg).

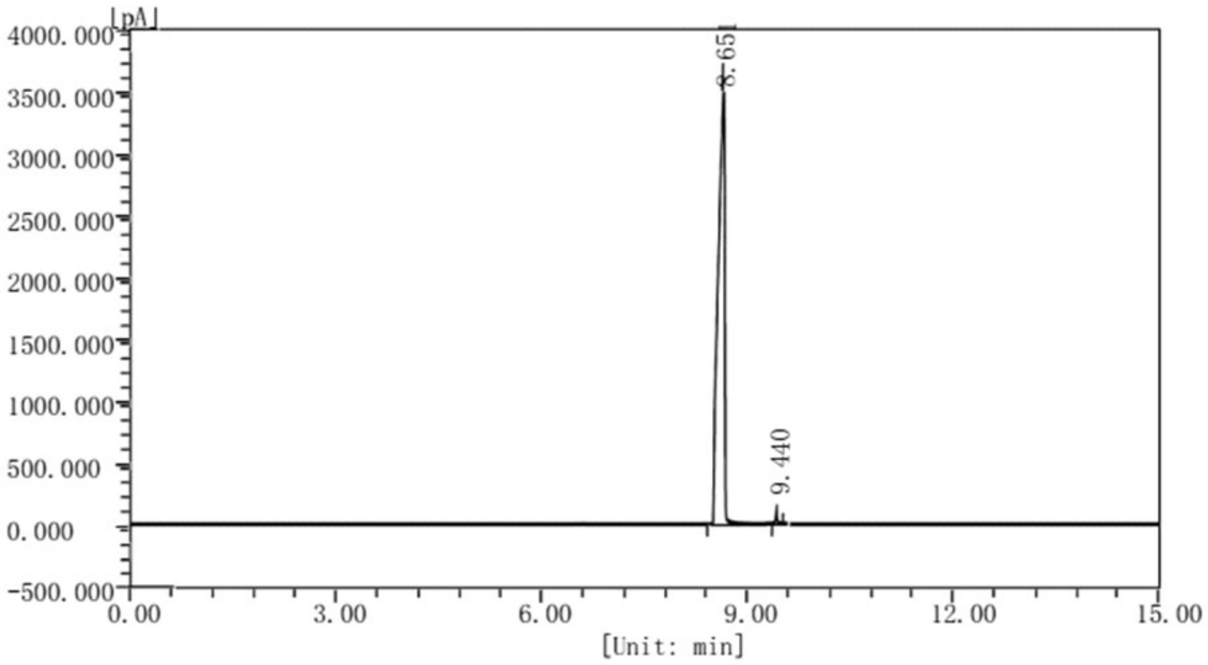

Analysis Report

Peak	Component	RetTime [min]	Height [uV]	Area [uV*s]	Area [%]	Content [%]
1	**Py**	8.651	401293.7	22452941.3	99.0421	99.0421
2	**Tol**	9.440	95666.5	201421.4	0.9579	0.9579
		Totals:	496960.2	22654362.7	100.0000	100.0000

Supplementary Figure 67. GC analysis of the 98:2 Py/Tol after adsorption by H-TCNB*α* (6.00 mg).

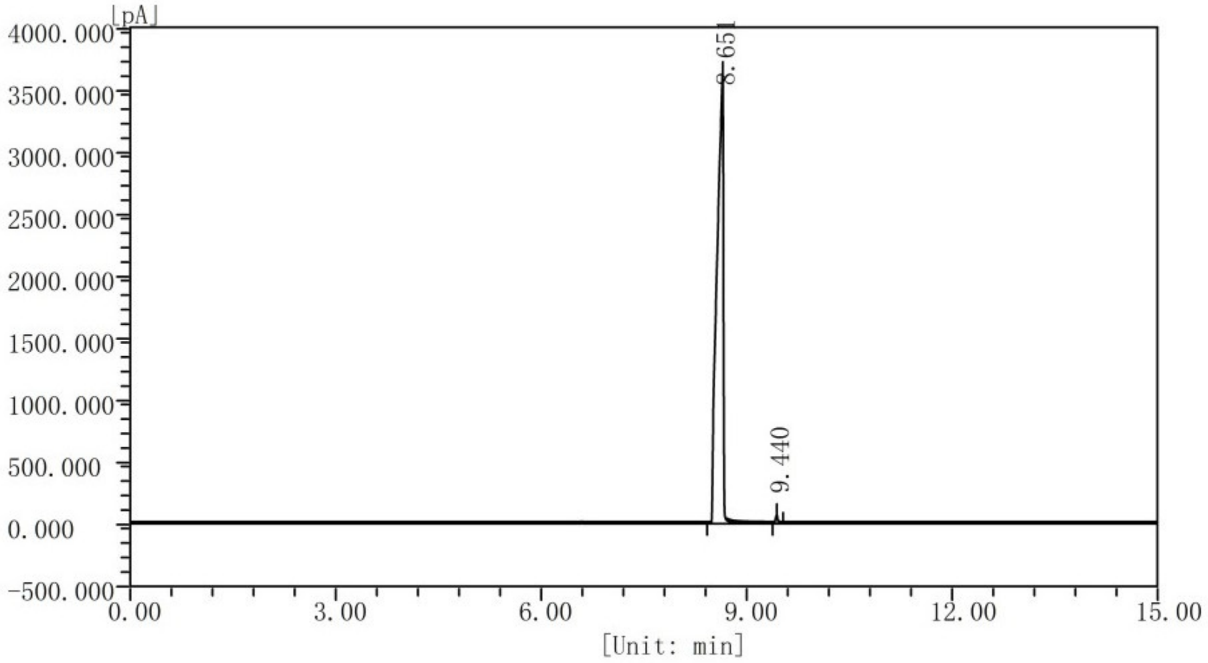

Analysis Report

Peak	Component	RetTime [min]	Height [uV]	Area [uV*s]	Area [%]	Content [%]
1	**Py**	8.651	3638153.3	23316906.7	99.3540	99.3540
2	**Tol**	9.440	69336.0	151597.3	0.6460	0.6460
		Totals:	3707489.3	23468504.0	100.0000	100.0000

Supplementary Figure 68. GC analysis of the 98:2 Py/Tol after adsorption by H-TCNB*α* (9.00 mg).

2.11. Recyclability of H-TCNB*α*

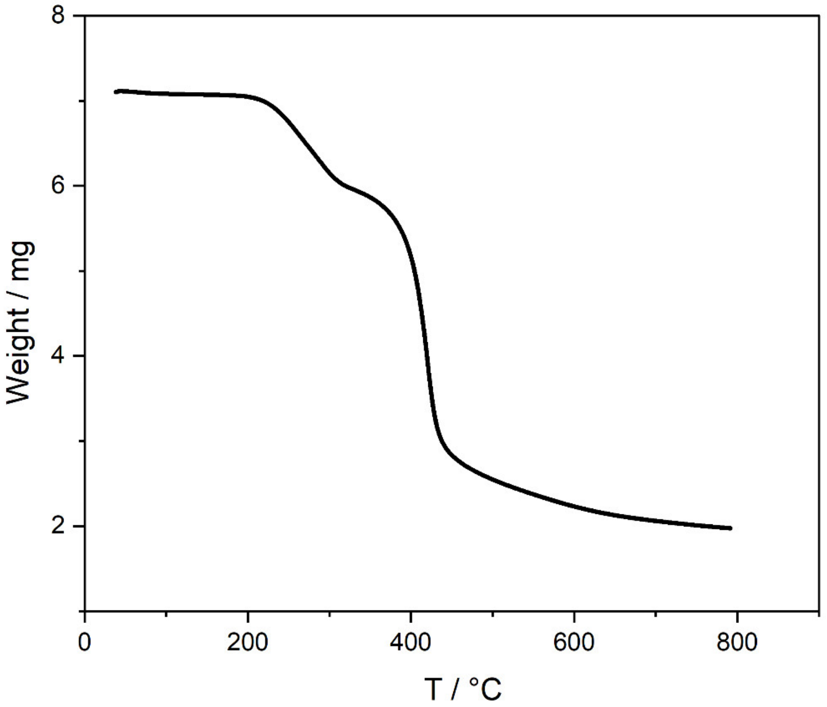

Supplementary Figure 69. Thermogravimetric analysis of H-TCNB@Bz after heating at 100 °C under vacuum for 9 h.

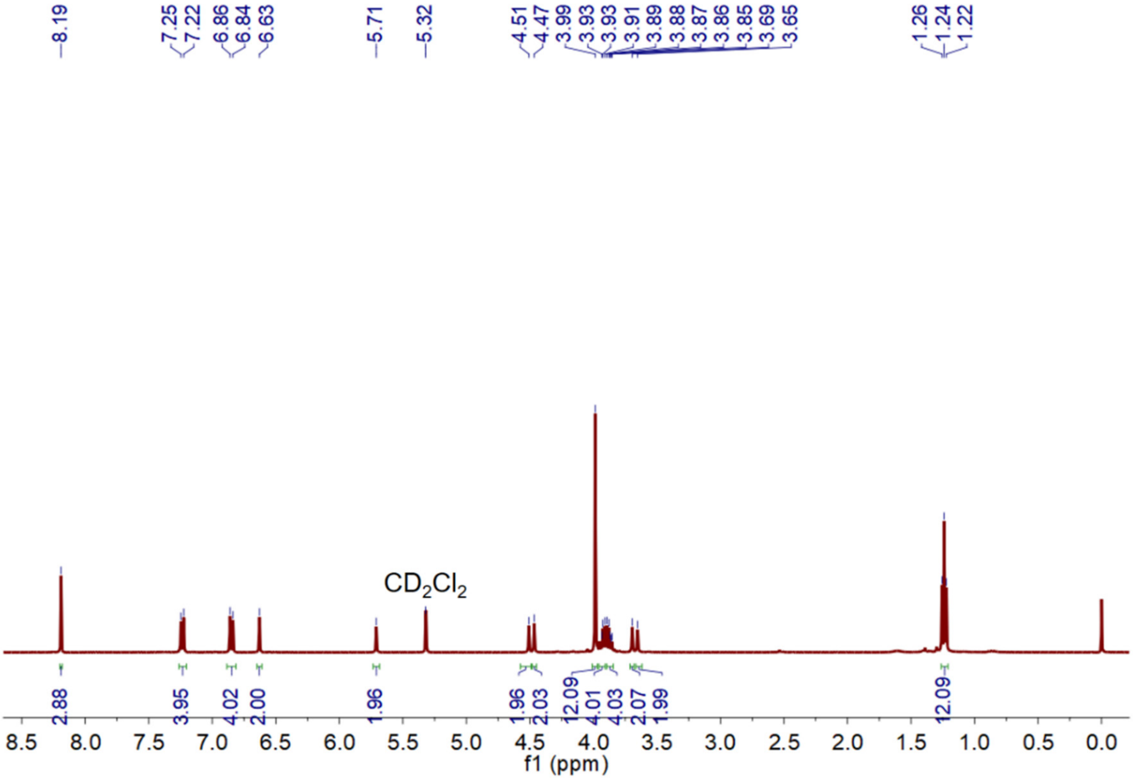

Supplementary Figure 70. ^{1}H NMR spectrum (500 MHz, CD_2Cl_2, 293 K) of H-TCNB@Bz after heating at 100 °C under vacuum for 9 h.

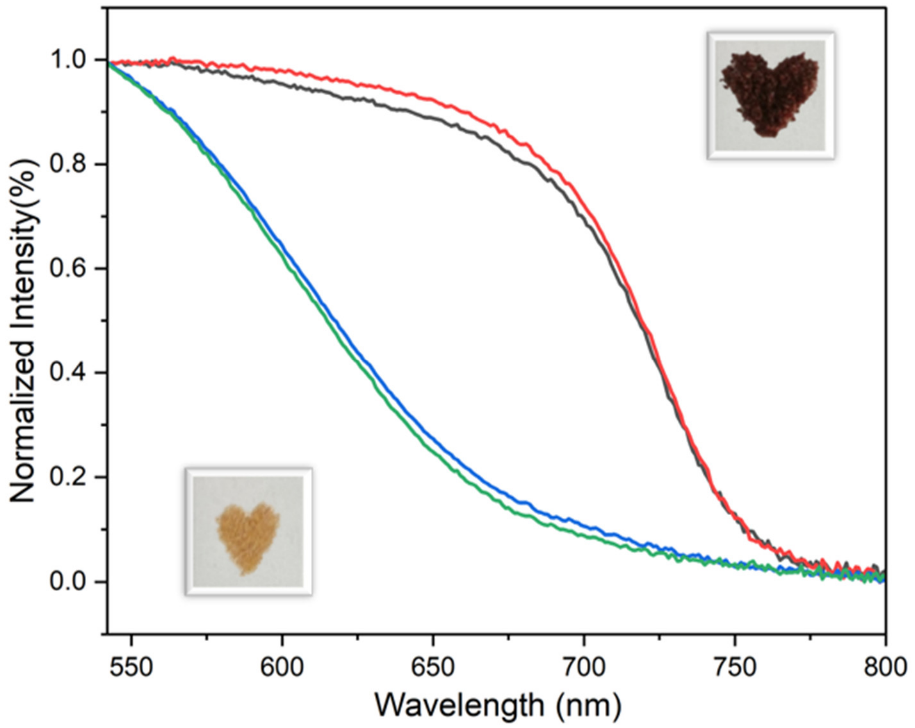

Supplementary Figure 71. Diffuse reflectance spectra of H-TCNB*α* (black line), re-activated H-TCNB*α* (red line), first uptake of Bz (blue line), and second uptake of Bz (green line).

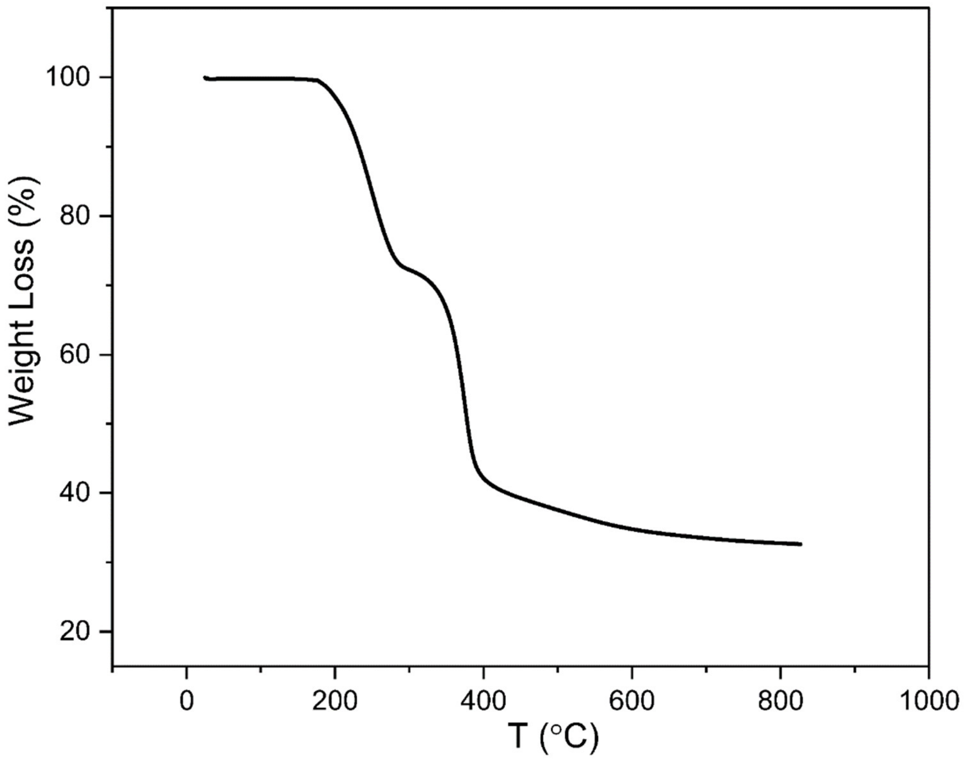

Supplementary Figure 72. Thermogravimetric analysis of H-TCNB@Tol after heating at 100 °C under vacuum for 9 h.

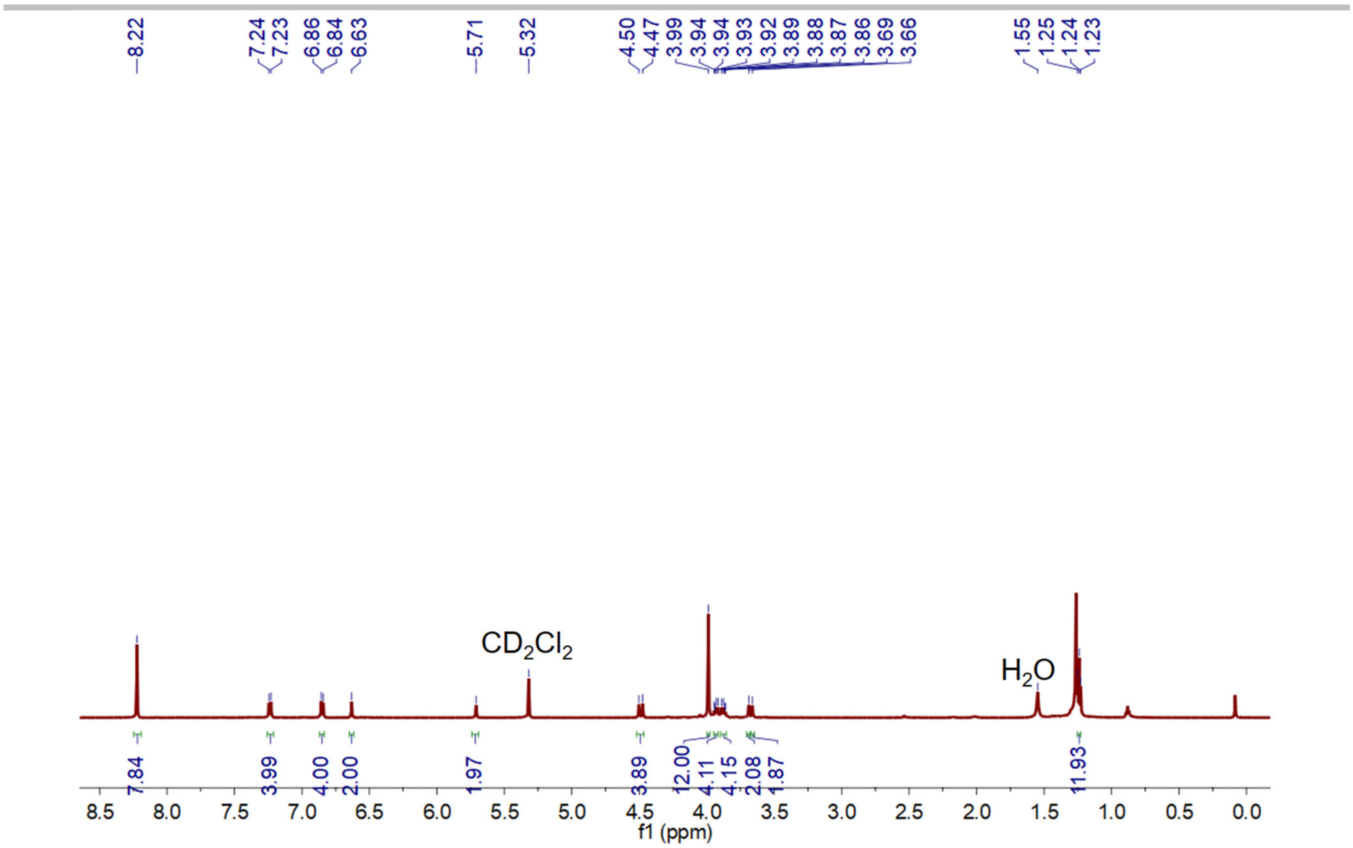

Supplementary Figure 73. ^{1}H NMR spectrum (500 MHz, CD_2Cl_2, 293 K) of H-TCNB@Tol after heating at 100 °C under vacuum for 9 h.

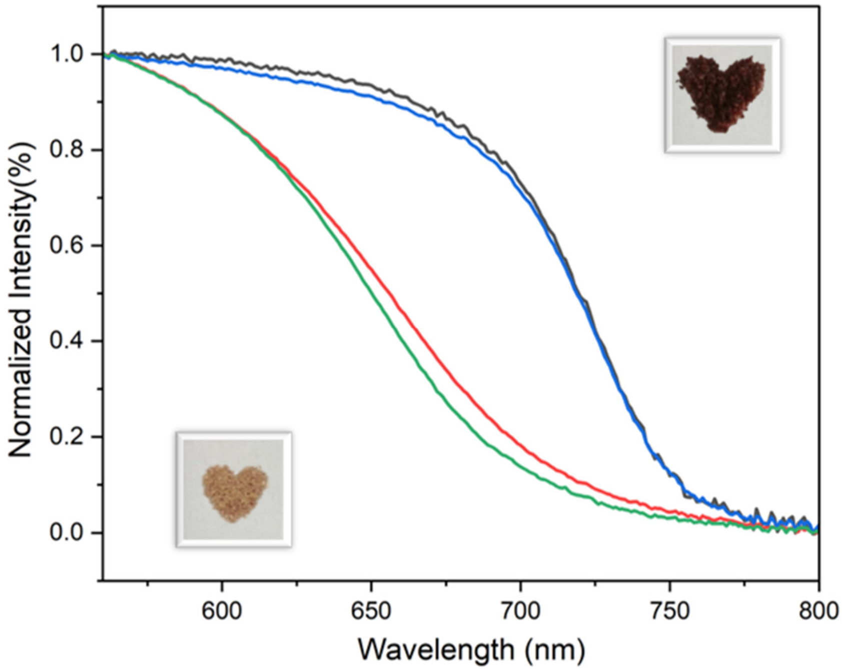

Supplementary Figure 74. Diffuse reflectance spectra of H-TCNB*α* (black line), re-activated H-TCNB*α* (red line), first uptake of Tol (blue line), and second uptake of Tol (green line).

2.12. Vapochromic behaviors experiments based on co-crystals of DON-TCNB*α* and DOB-TCNB*α*

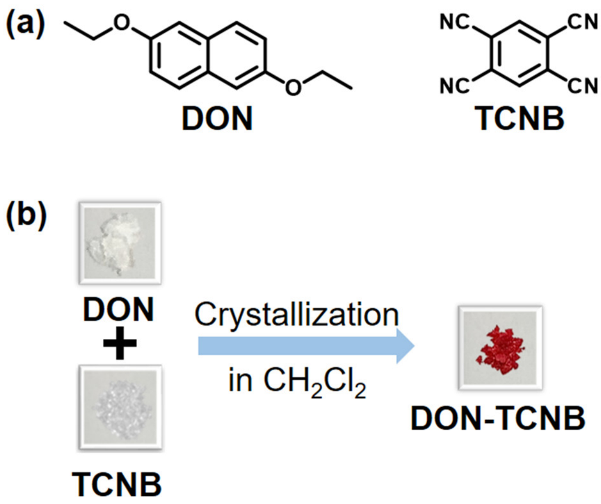

Supplementary Figure 75. (a) Chemical structures of DON and TCNB. (b) Images of DON, TCNB and DON-TCNB.

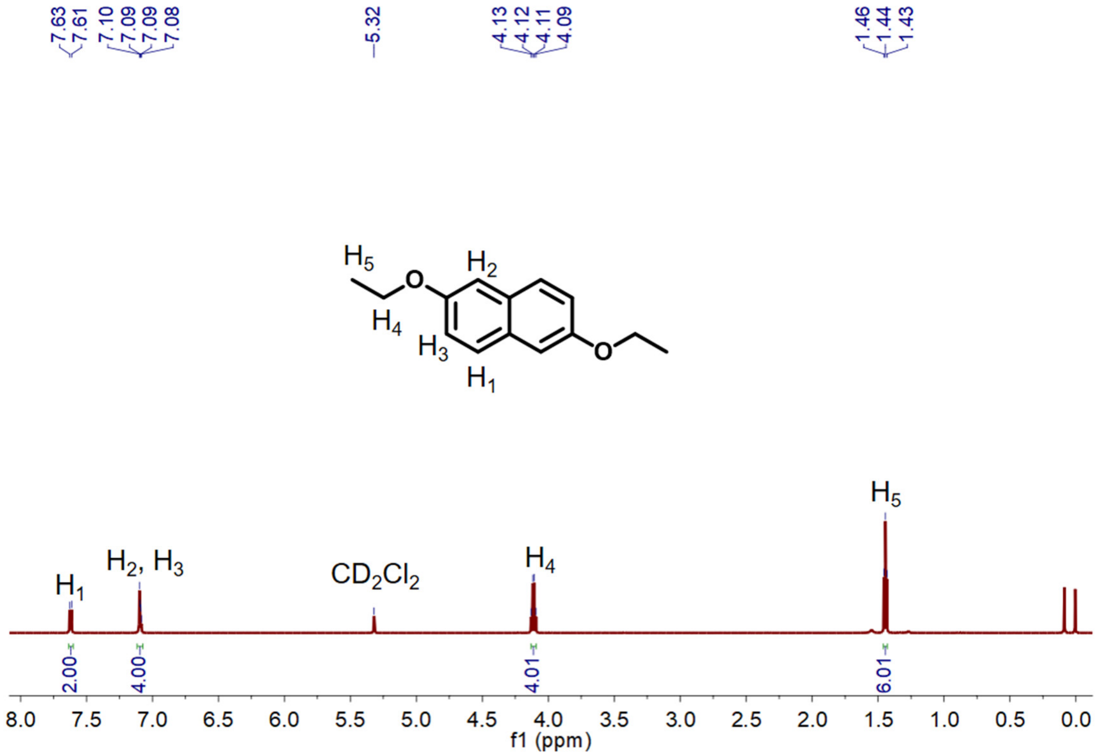

Supplementary Figure 76. ^1H NMR spectrum (600 MHz, CD_2Cl_2, 293 K) of DON.

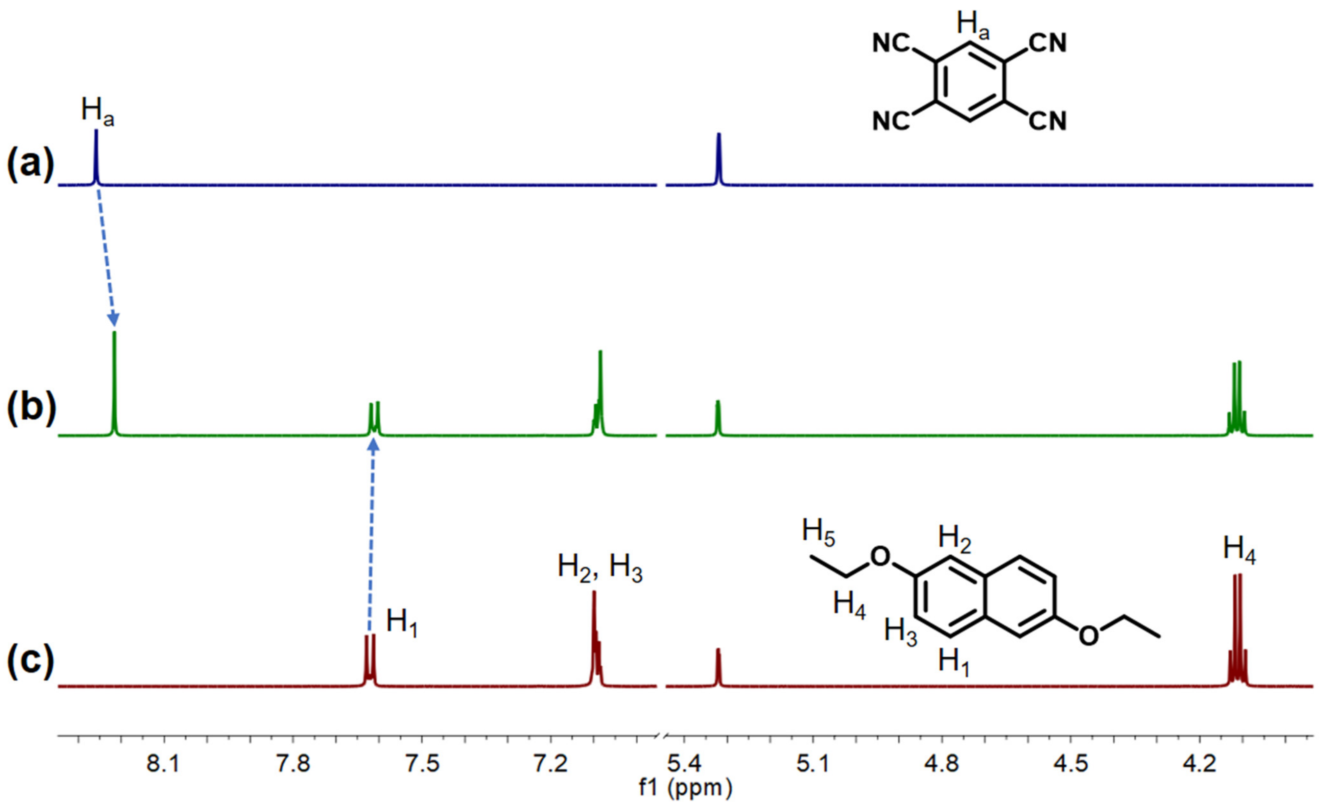

Supplementary Figure 77. Partial ^{1}H NMR spectra (600 MHz, CD_2Cl_2, 293 K) of (a) free TCNB (10.0 mM), (b) DON (10.0 mM) and TCNB (10.0 mM), and (c) free DON (10.0 mM).

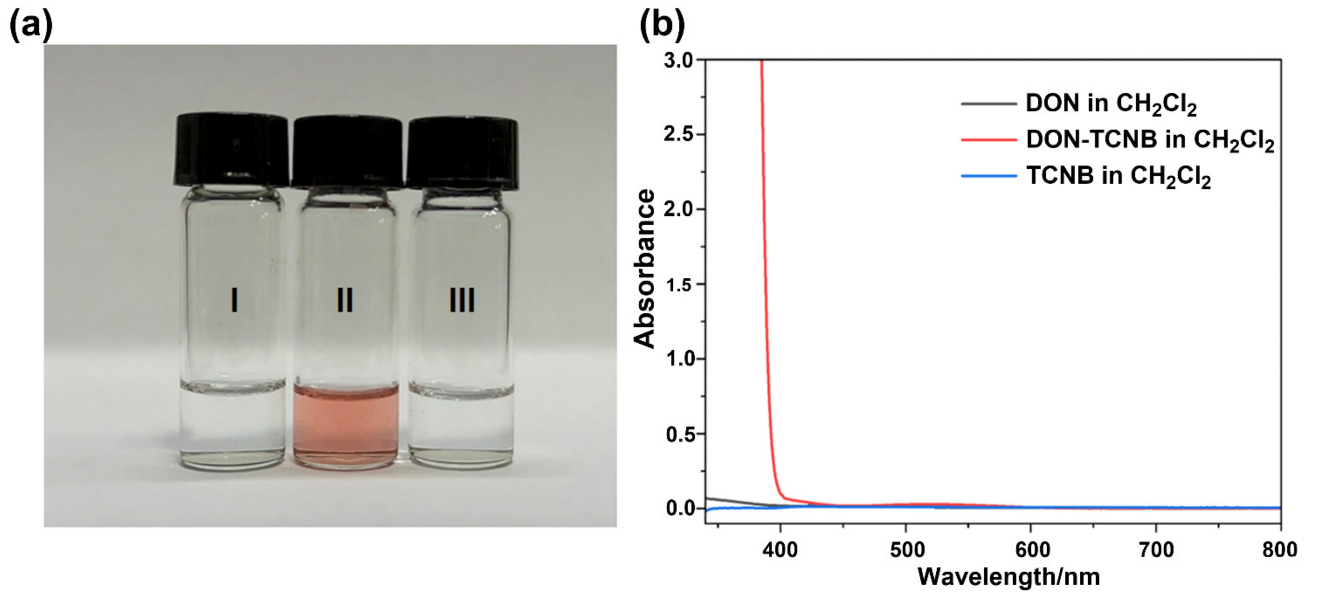

Supplementary Figure 78. (a) Images: (I) DON (5.00 mM) in CH_2Cl_2; (II) DON (5.00 mM) and TCNB (5.00 mM) in CH_2Cl_2; (III) TCNB (5.00 mM) in CH_2Cl_2. (b) UV-*vis* spectra: DON (5.00 mM); DON (5.00 mM) and TCNB (5.00 mM); TCNB (5.00 mM).

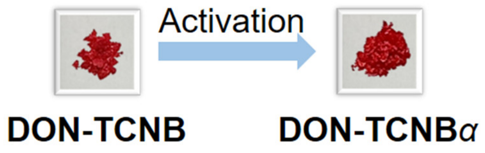

Supplementary Figure 79. Images of DON-TCNB and DON-TCNBα.

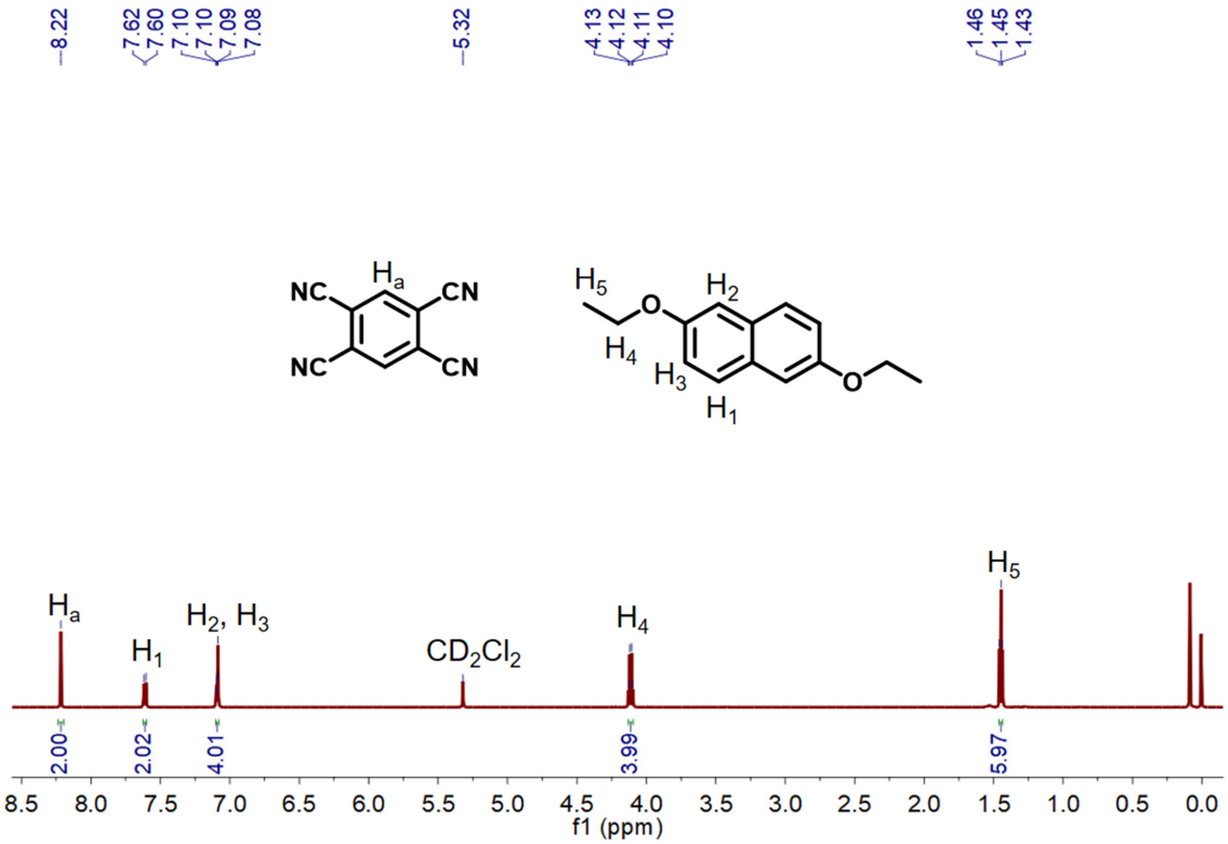

Supplementary Figure 80. ^{1}H NMR spectrum (500 MHz, CD_2Cl_2, 293 K) of DON-TCNBα.

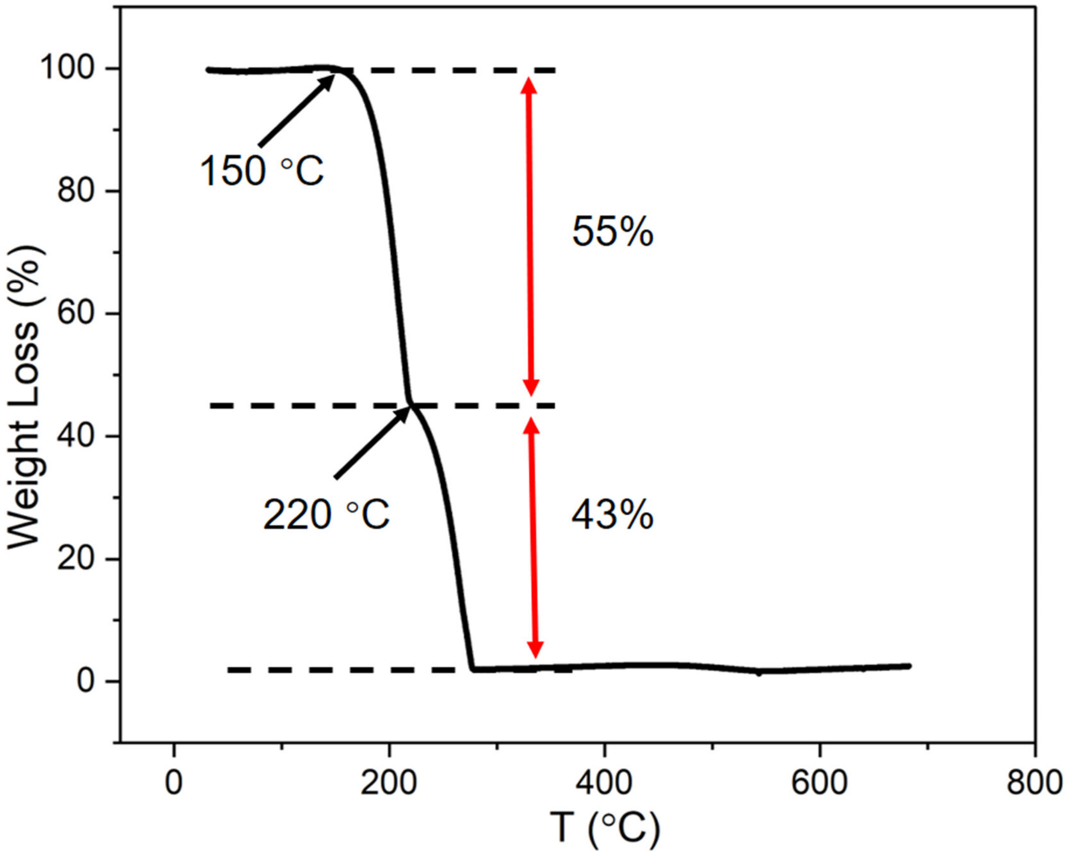

Supplementary Figure 81. Thermogravimetric analysis of DON-TCNBα.

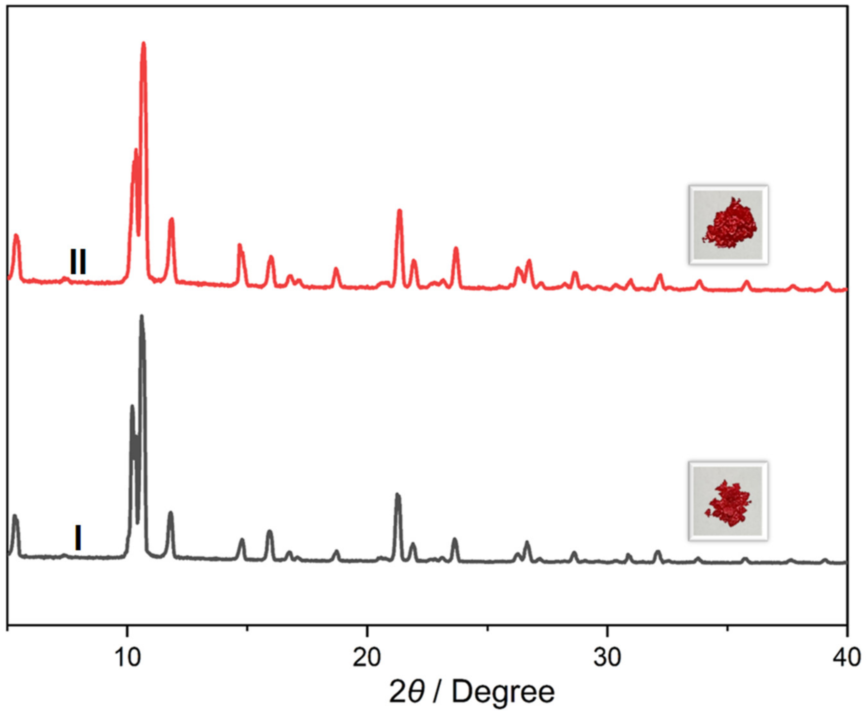

Supplementary Figure 82. PXRD patterns and images of (I) DON-TCNB and (II) DON-TCNBα.

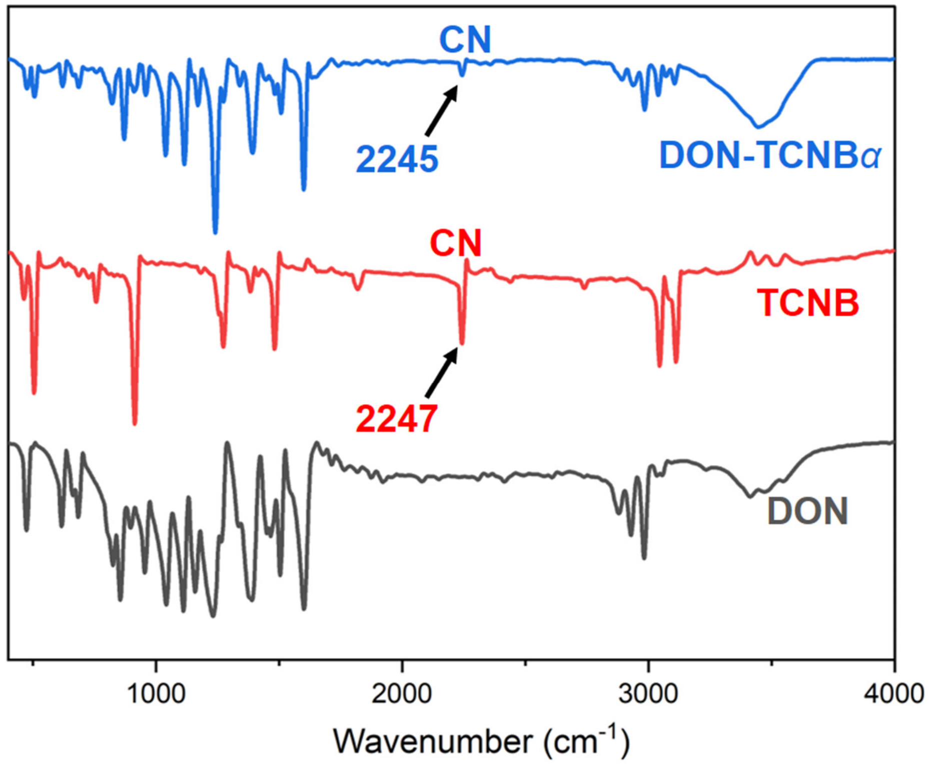

Supplementary Figure 83. FT-IR spectra of DON, TCNB and DON-TCNB*α*.

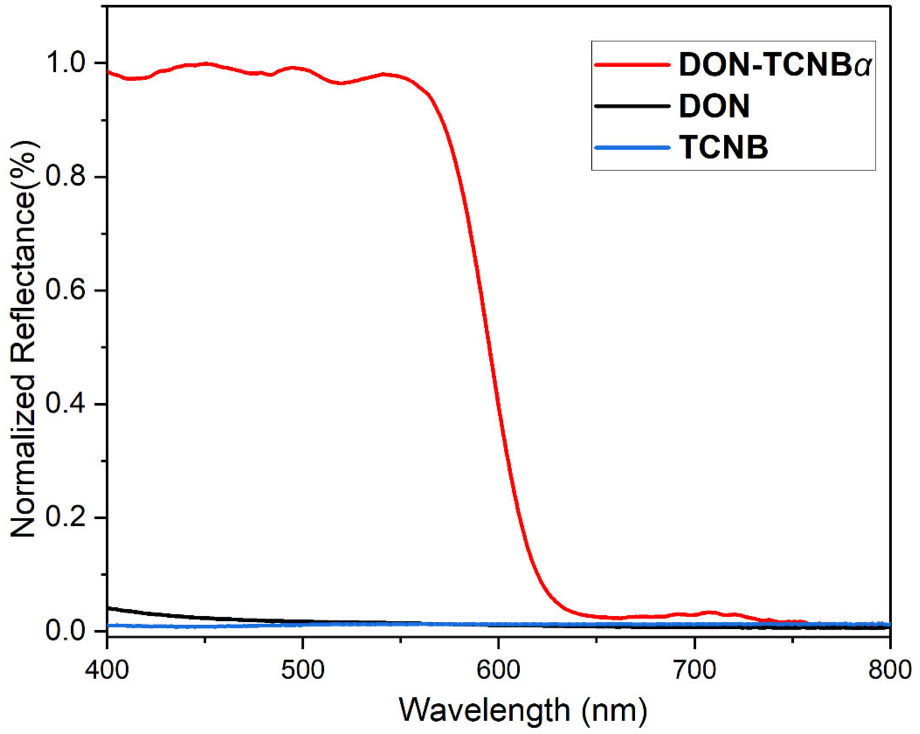

Supplementary Figure 84. Diffuse reflectance spectra and images of DON-TCNB*α*, DON and TCNB.

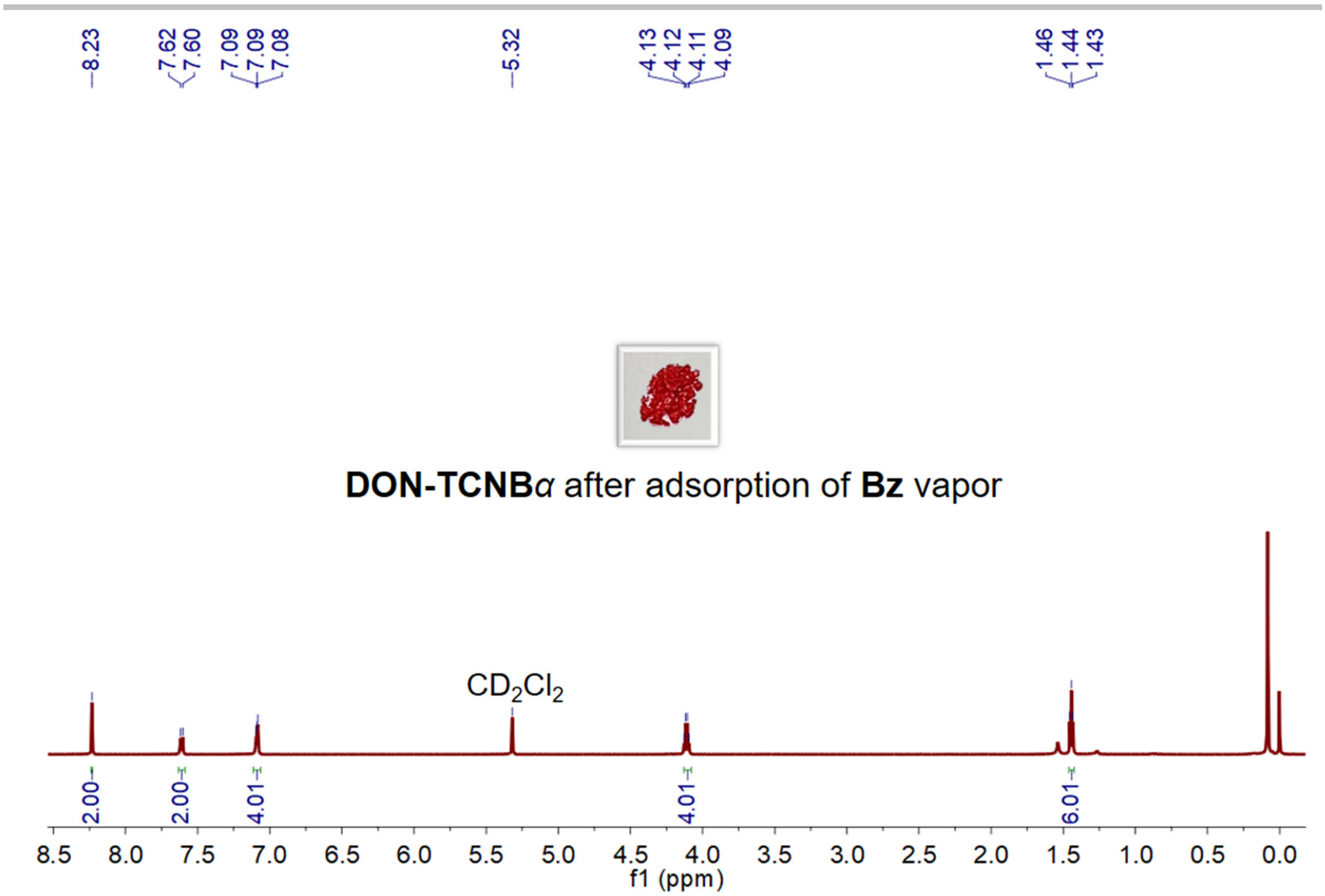

Supplementary Figure 85. ^{1}H NMR spectrum (600 MHz, CD_2Cl_2, 293 K) of DON-TCNB*α* after adsorption of Bz vapor for 24 h.

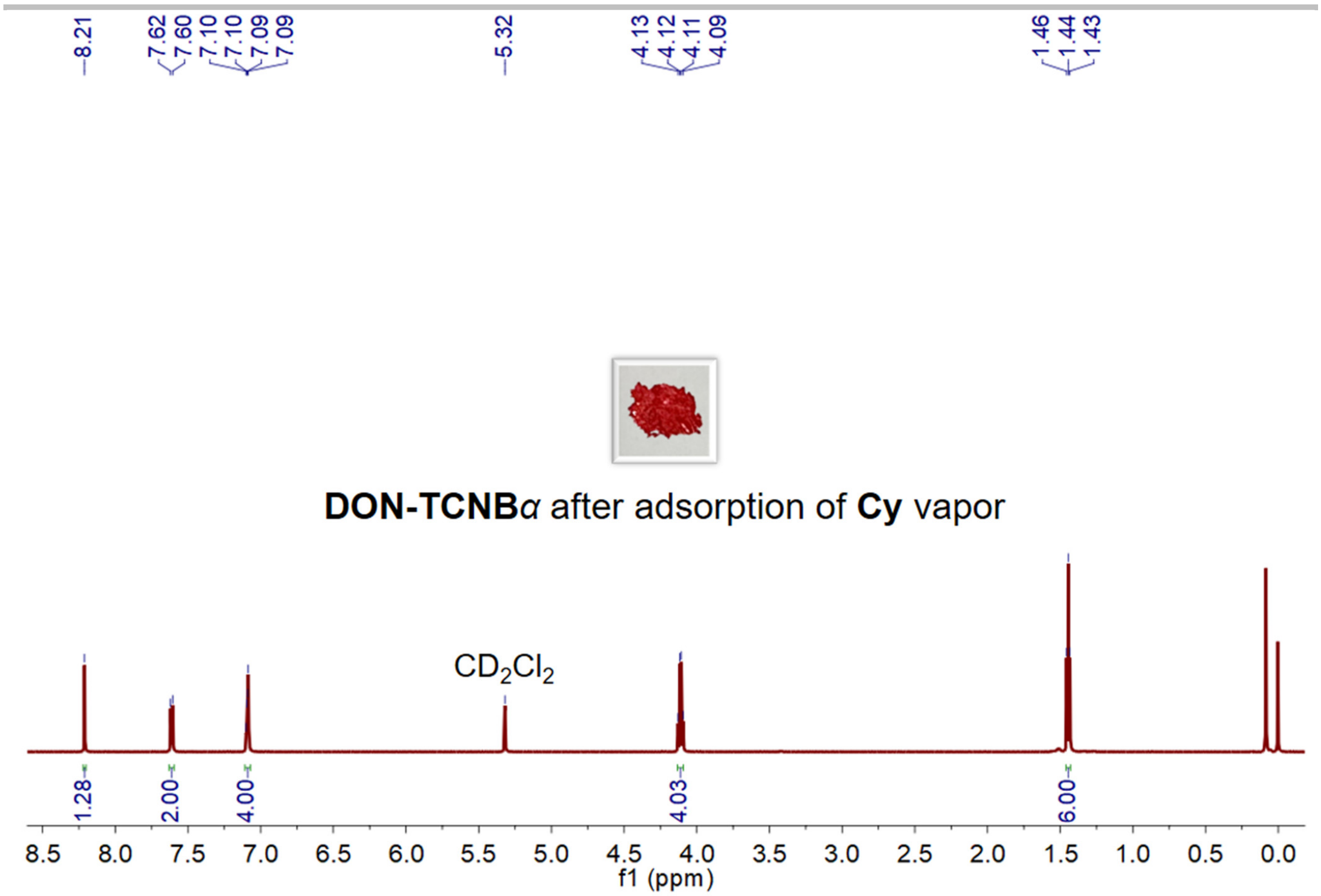

Supplementary Figure 86. ^{1}H NMR spectrum (600 MHz, CD_2Cl_2, 293 K) of DON-TCNB*α* after adsorption of Cy vapor for 24 h.

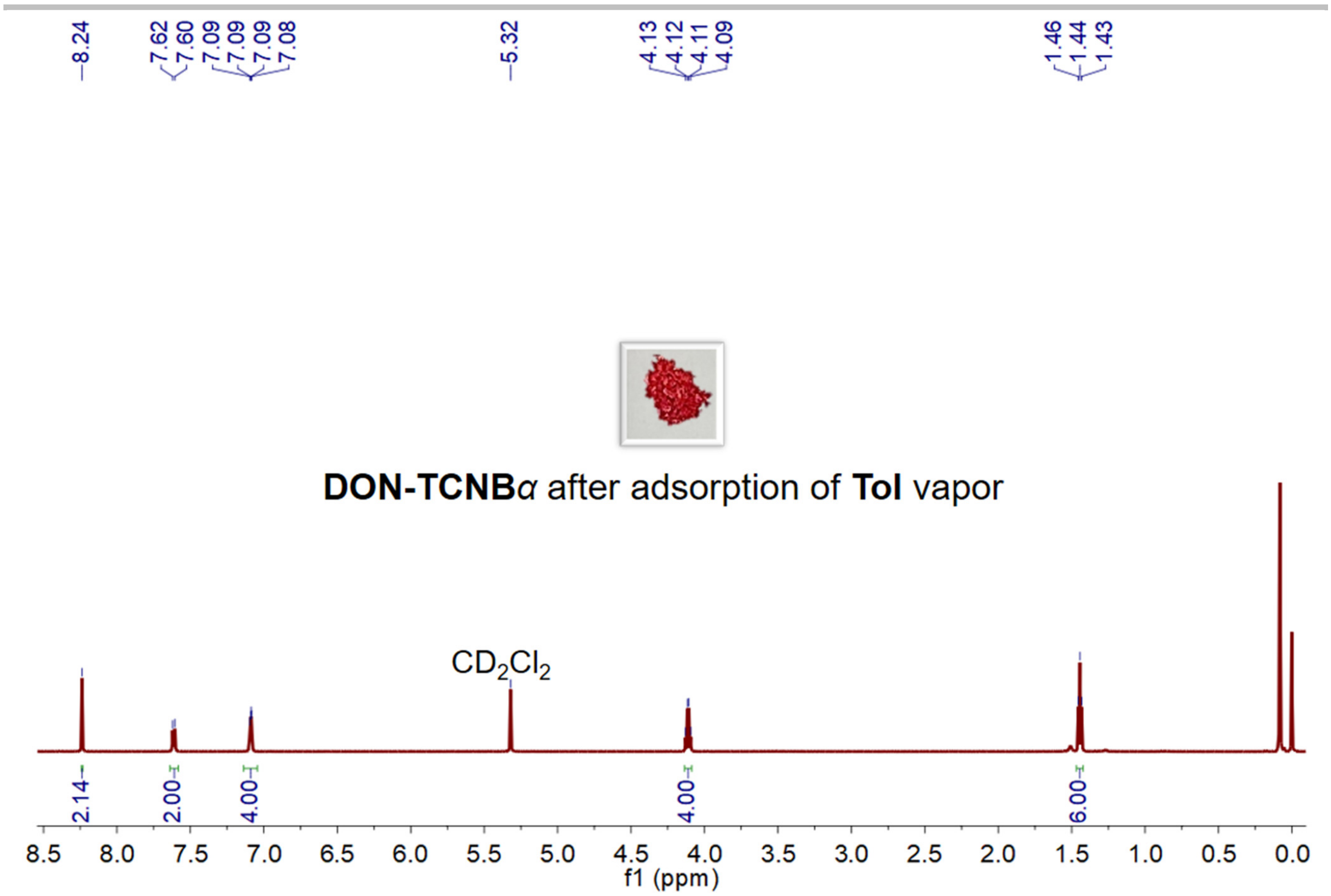

Supplementary Figure 87. ^{1}H NMR spectrum (600 MHz, CD_2Cl_2, 293 K) of DON-TCNB*α* after adsorption of Tol vapor for 24 h.

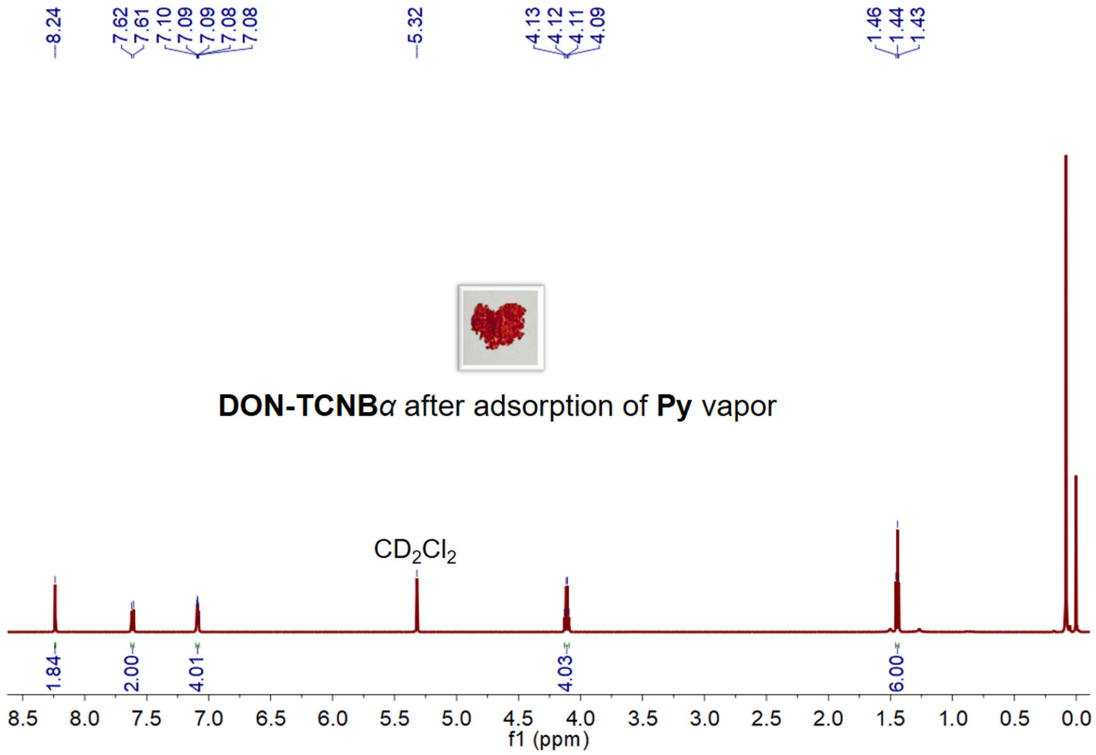

Supplementary Figure 88. ^{1}H NMR spectrum (600 MHz, CD_2Cl_2, 293 K) of DON-TCNB*α* after adsorption of Py vapor for 24 h.

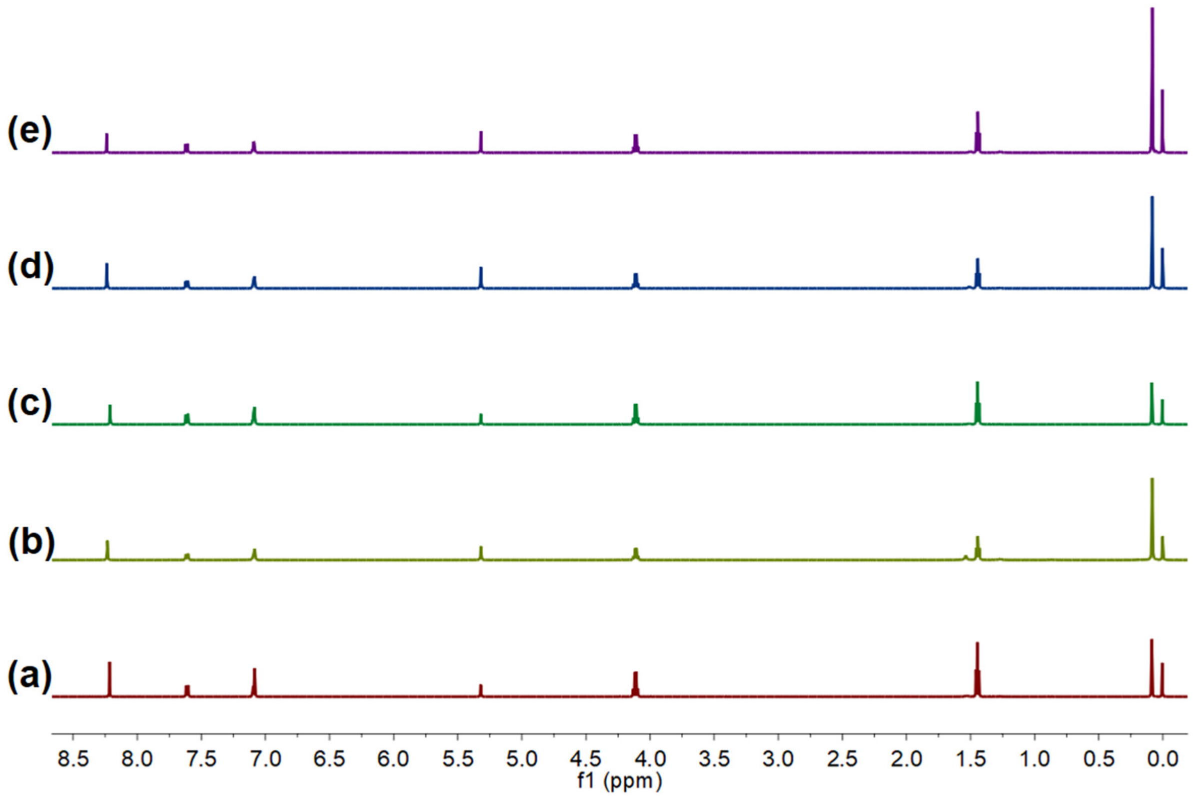

Supplementary Figure 89. ^{1}H NMR spectra (600 MHz, CD_2Cl_2, 293 K) of DON-TCNBα: (a) original DON-TCNBα; after adsorption of (b) Bz vapor; (c) Cy vapor; (d) Tol vapor and (e) Py vapor, respectively.

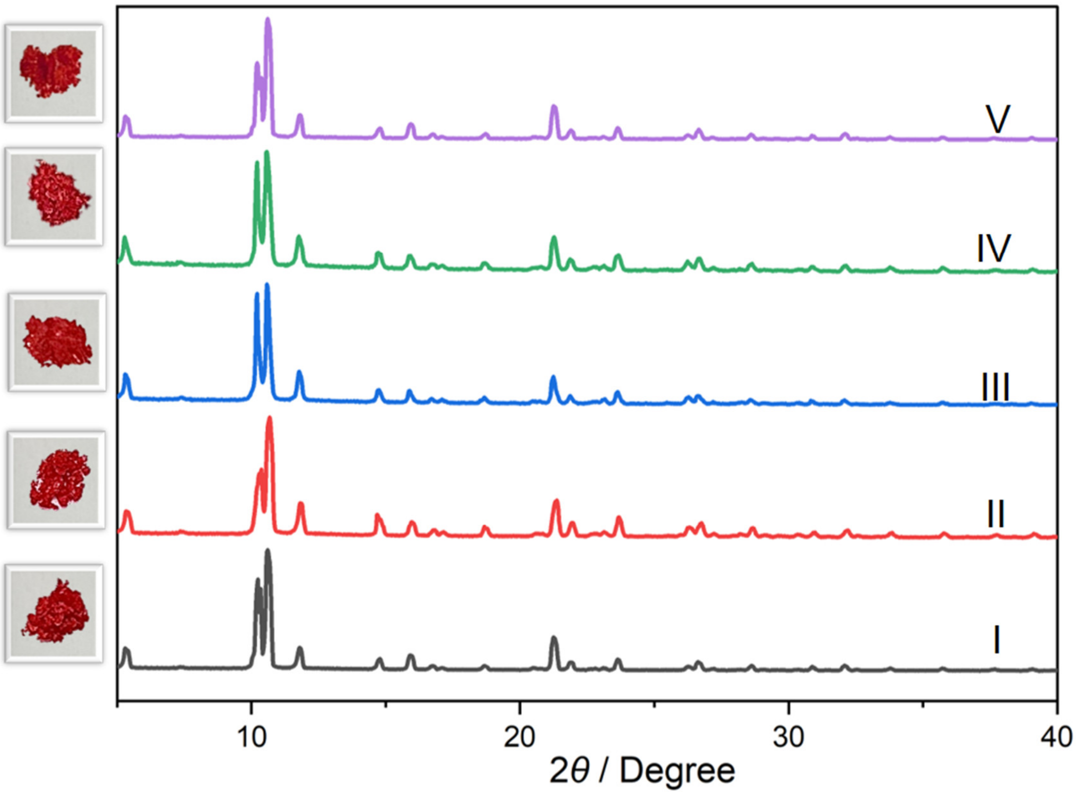

Supplementary Figure 90. PXRD patterns of DON-TCNBα: (I) original DON-TCNBα; (II) after adsorption of Bz vapor; (III) after adsorption of Cy vapor; (IV) after adsorption of Tol vapor; (V) after adsorption of Py vapor.

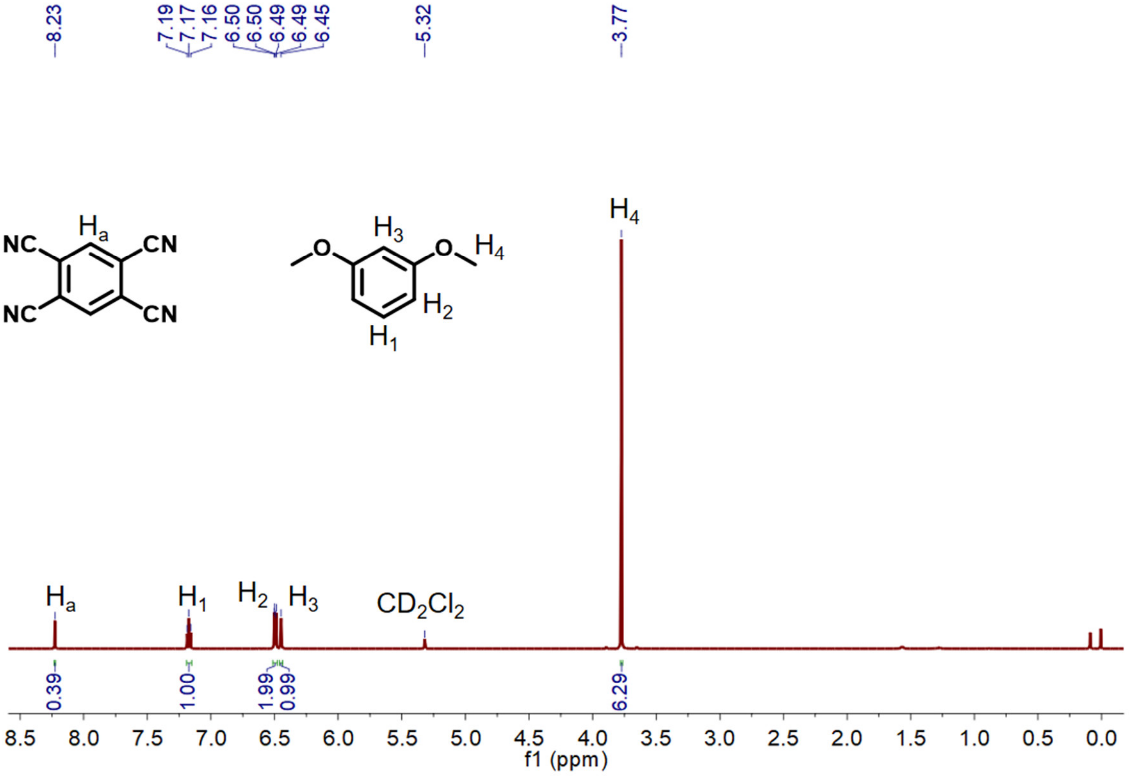

Supplementary Figure 91. ^{1}H NMR spectrum (600 MHz, CD_2Cl_2, 293 K) of DOB-TCNB*α*.

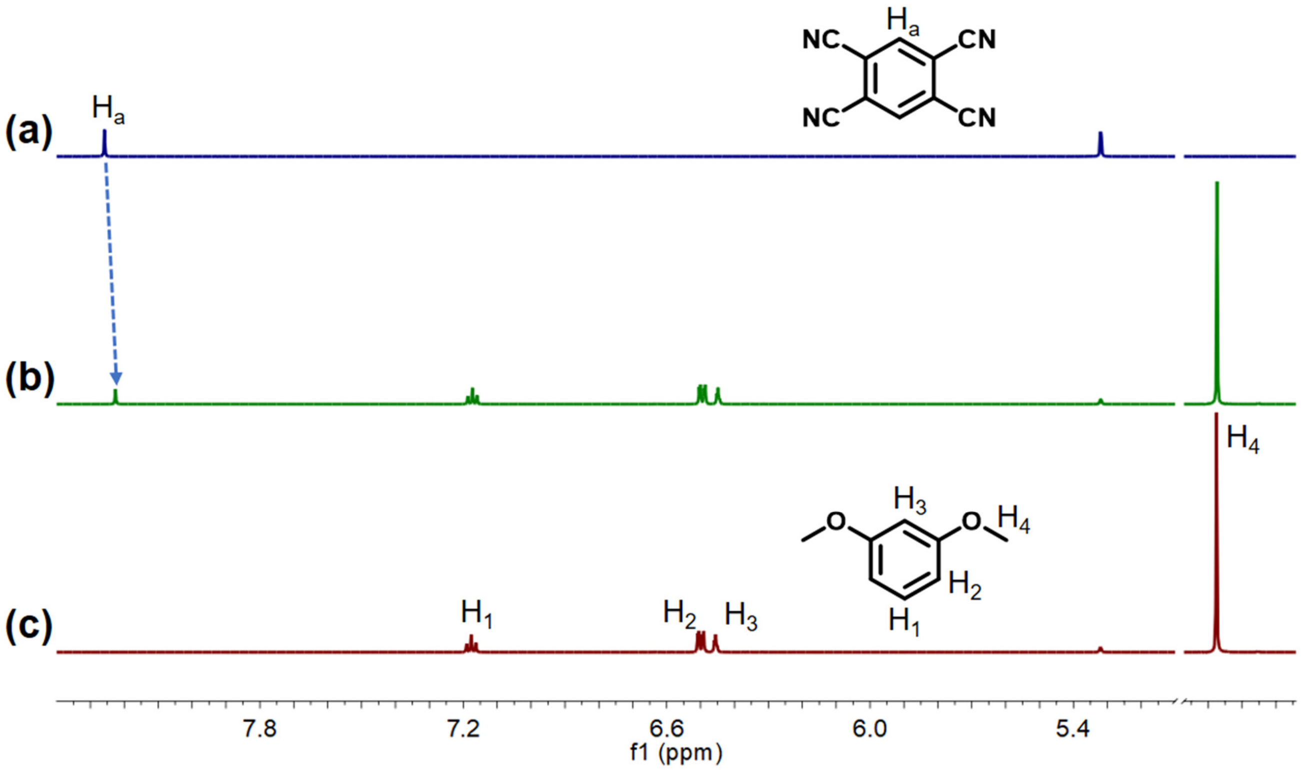

Supplementary Figure 92. Partial ^{1}H NMR spectra (500 MHz, CD_2Cl_2, 293 K) of (a) free TCNB (10.0 mM), (b) DOB (10.0 mM) and TCNB (10.0 mM), and (c) free DOB (10.0 mM).

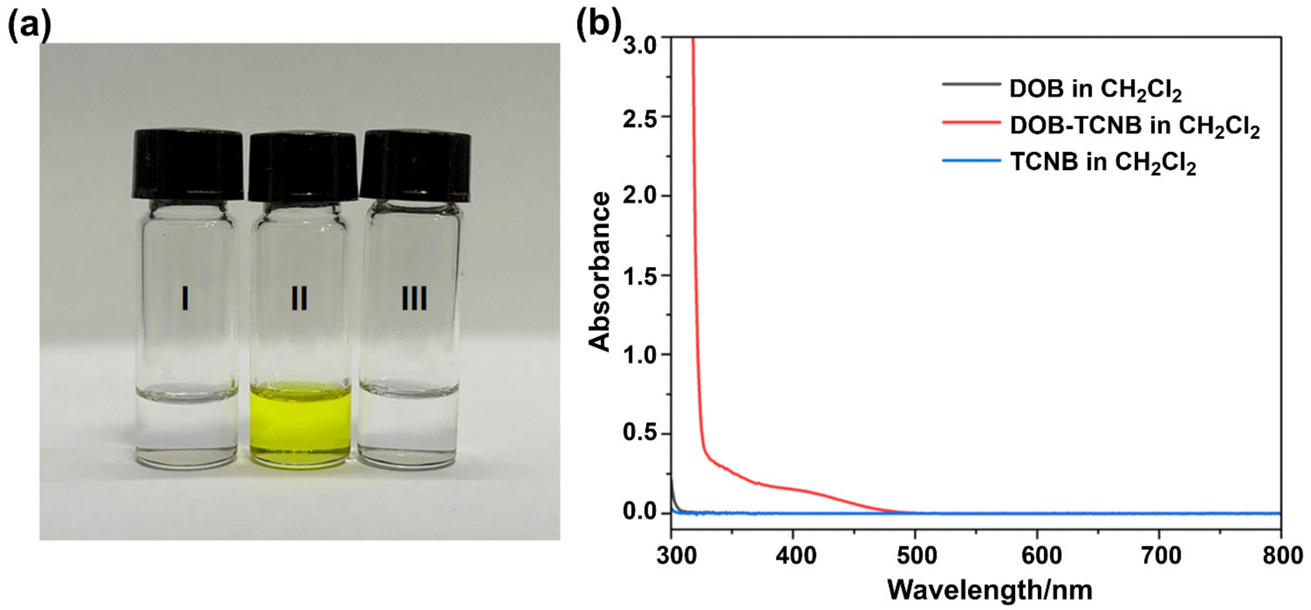

Supplementary Figure 93. (a) Images: (I) DOB (5.00 mM) in CH_2Cl_2; (II) DOB (5.00 mM) and TCNB (5.00 mM) in CH_2Cl_2; (III) TCNB (5.00 mM) in CH_2Cl_2. (b) UV-*vis* spectra: DOB (5.00 mM); DOB (5.00 mM) and TCNB (5.00 mM); TCNB (5.00 mM).

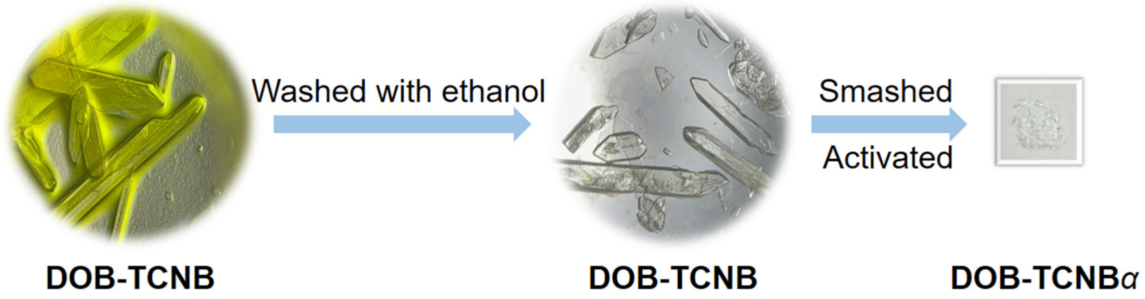

Supplementary Figure 94. Images of DOB, DOB-TCNB and DOB-TCNB*α*.

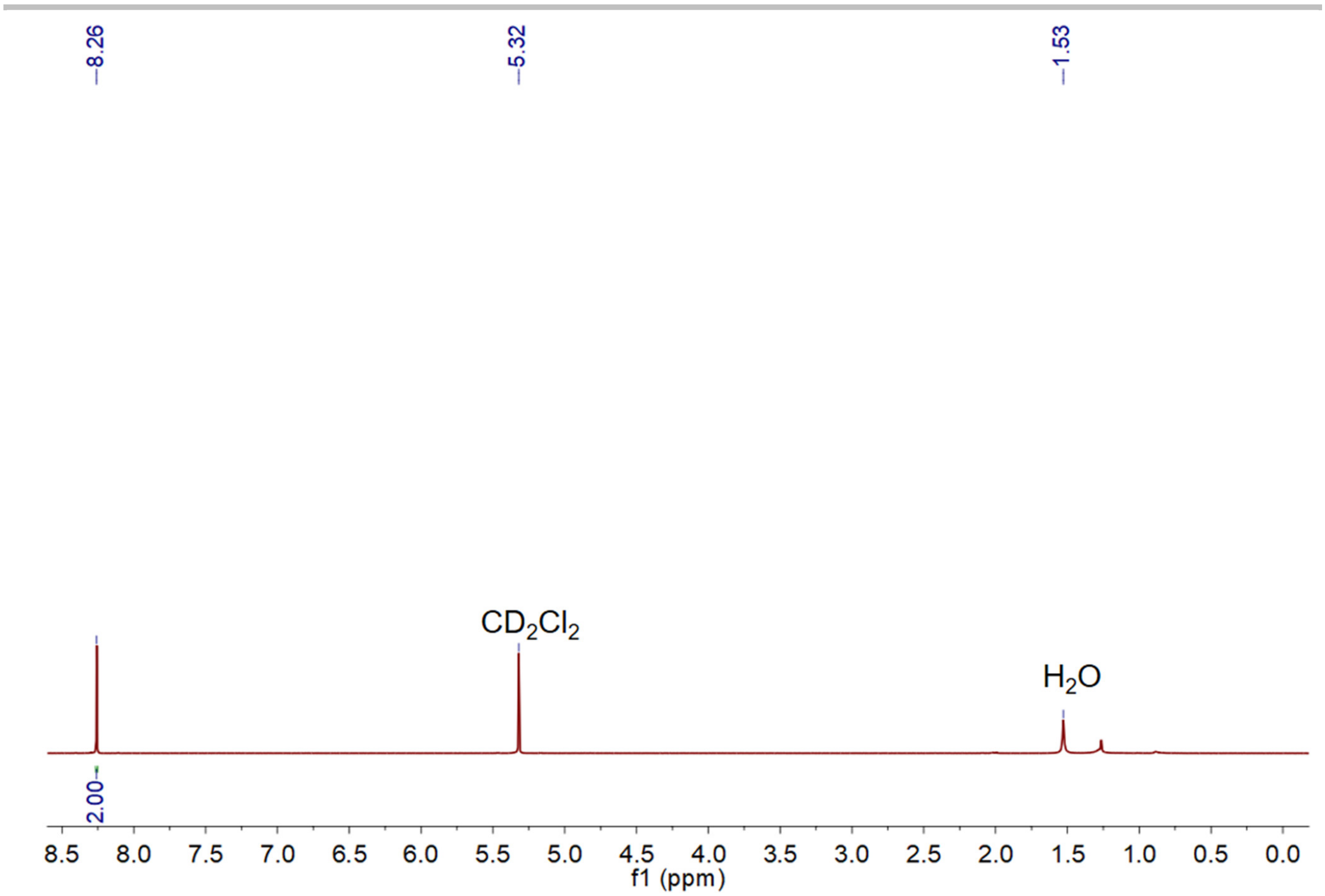

Supplementary Figure 95. ^{1}H NMR spectrum (600 MHz, CD_2Cl_2, 293 K) of DOB-TCNBα.

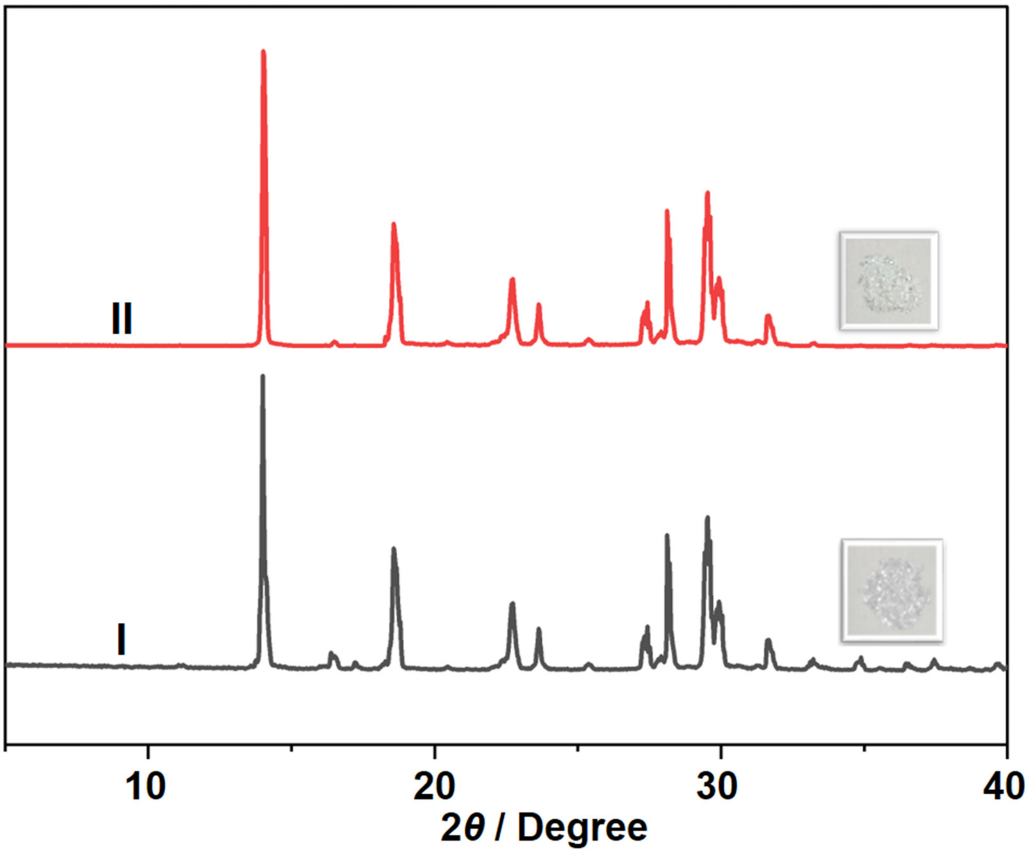

Supplementary Figure 96. PXRD patterns: (I) TCNB; (II) DOB-TCNBα.

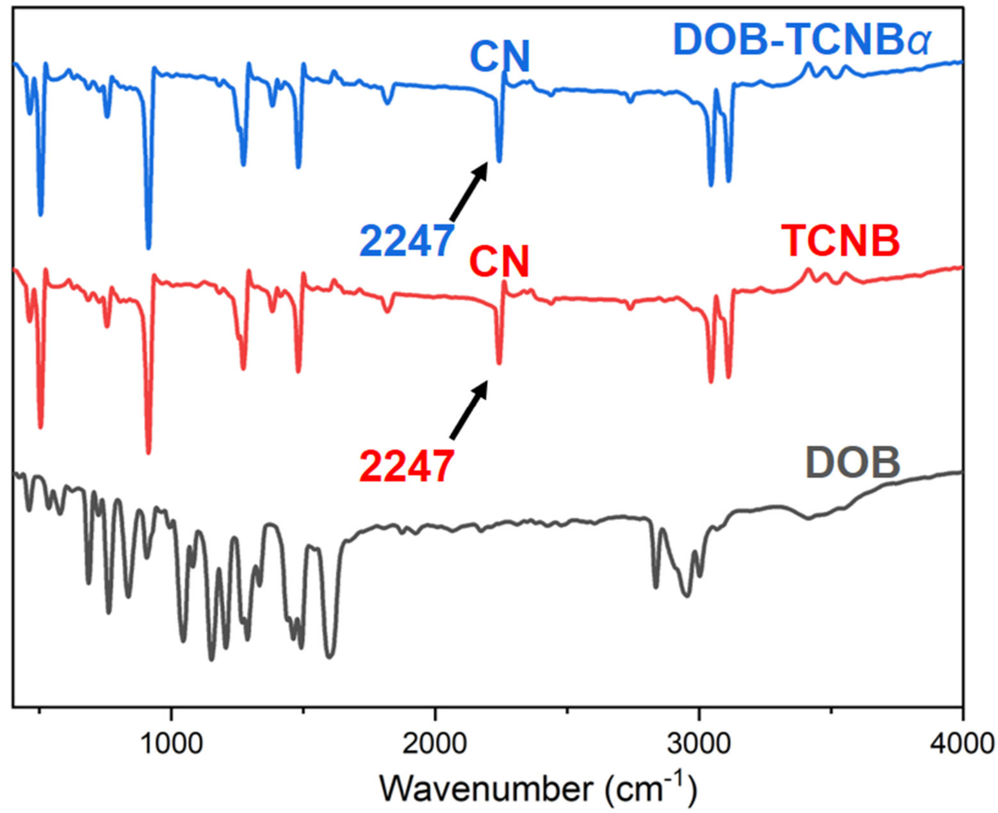

Supplementary Figure 97. FT-IR spectra of DOB, TCNB and DOB-TCNBα.

3. Supplementary Reference

1. Zhou, J., Yu, G., Li, Q., Wang, M. & Huang, F. Separation of benzene and cyclohexane by nonporous adaptive crystals of a hybrid[3]arene. *J. Am. Chem. Soc*. **142**, 2228–2232 (2020).
