# Supplementary material for: Separation of benzene and toluene associated with vapochromic behaviors by hybrid[4]arene-based co-crystals
Source: Nat Commun. 2024 Feb 10;15:1260. doi: 10.1038/s41467-024-45592-6 (PMC10858960; doi:10.1038/s41467-024-45592-6)
Supplement: Supplementary file 3 — Description of Additional Supplementary Files [file 41467_2024_45592_MOESM3_ESM.pdf]

Description of Additional Supplementary Files

File Name: Supplementary Data 1
Description: Cartesian Coordinates of the optimised structures for H-TCNB@Py.

File Name: Supplementary Data 2
Description: Cartesian Coordinates of the optimised structures for H-TCNB@Tol.
